# Stress-induced reversible cell-cycle arrest requires PRC2/PRC1-mediated control of mitophagy in *Drosophila* germline stem cells and human iPSCs

**DOI:** 10.1016/j.stemcr.2022.11.004

**Published:** 2022-12-08

**Authors:** Tommy H. Taslim, Abdiasis M. Hussein, Riya Keshri, Julien R. Ishibashi, Tung C. Chan, Bich N. Nguyen, Shuozhi Liu, Daniel Brewer, Stuart Harper, Scott Lyons, Ben Garver, Jimmy Dang, Nanditaa Balachandar, Samriddhi Jhajharia, Debra del Castillo, Julie Mathieu, Hannele Ruohola-Baker

**Affiliations:** 1 Department of Biochemistry, University of Washington, Seattle, WA, USA; 2 Institute for Stem Cell and Regenerative Medicine, University of Washington, School of Medicine, Seattle, WA, USA; 3 Department of Biotechnology, School of Bioengineering, Faculty of Engineering and Technology, SRM Institute of Science and Technology, Kattankulathur, India; 4 Department of Comparative Medicine, University of Washington, Seattle, WA, USA

**Keywords:** mitochondria, cyclin E, mitophagy, mTOR, pluripotent stem cells, epigenetic, PRC2

## Abstract

Following acute genotoxic stress, both normal and tumorous stem cells can undergo cell-cycle arrest to avoid apoptosis and later re-enter the cell cycle to regenerate daughter cells. However, the mechanism of protective, reversible proliferative arrest, “quiescence,” remains unresolved. Here, we show that mitophagy is a prerequisite for reversible quiescence in both irradiated *Drosophila* germline stem cells (GSCs) and human induced pluripotent stem cells (hiPSCs). In GSCs, mitofission (Drp1) or mitophagy (Pink1/Parkin) genes are essential to enter quiescence, whereas mitochondrial biogenesis (PGC1α) or fusion (Mfn2) genes are crucial for exiting quiescence. Furthermore, mitophagy-dependent quiescence lies downstream of mTOR- and PRC2-mediated repression and relies on the mitochondrial pool of cyclin E. Mitophagy-dependent reduction of cyclin E in GSCs and in hiPSCs during mTOR inhibition prevents the usual G1/S transition, pushing the cells toward reversible quiescence (G0). This alternative method of G1/S control may present new opportunities for therapeutic purposes.

## Highlights

• Mitophagy is required for entry into reversible quiescence in *Drosophila* GSC and hiPSC
• PRC1/2-mediated gene repression is required for injury-induced mitophagy
• Cytoplasmic cyclin E associates with the outer mitochondrial membrane
• Mitochondrial degradation reduces cytoplasmic cyclin E resulting into quiescence

## Graphical abstract

**Figure:**
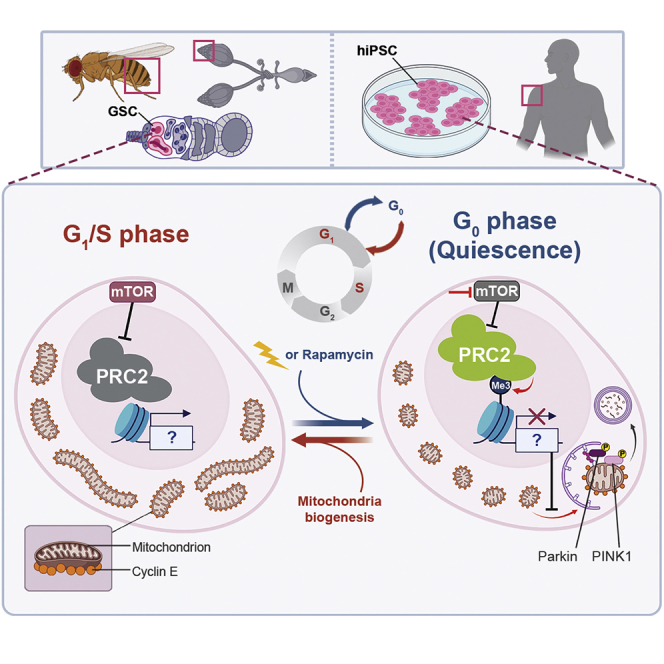

## Teaser

In this work, Ruohola-Baker and colleagues propose a novel mechanism of quiescence conserved in *Drosophila* GSCs and human iPSCs. They show that stress-induced quiescence requires PRC1/2-mediated epigenetic gene repression to induce mitophagy. Mitophagy is required to enter quiescence. Cytoplasmic cyclin E localized on the outer mitochondrial membrane is degraded during mitophagy, preventing G1/S transition, compelling the cell to enter the G0 or quiescence state.

## Introduction

Diverse types of stem cells have the capacity to exit the cell cycle upon stress, only to re-enter under the appropriate conditions; this process, coined “quiescence,” is distinct from senescence because quiescence can normally be reversed. Nutrient-sensitive mechanistic target of rapamycin (mTOR) signaling has been implicated in quiescence, with mTOR activation promoting proliferation and exit from quiescence, and mTOR repression being a hallmark of stem cells in quiescence (van Velthoven and Rando, 2019; Cho et al., 2019; Meng et al., 2018; Artoni et al., 2017) and embryonic diapause (Bulut-Karslioglu et al., 2016; Hussein et al., 2020; Arena et al., 2021), an extreme example of developmental quiescence, with some exceptions (Mathieu et al., 2019). Moreover, quiescence is associated with decreased mitochondrial metabolism and increased macroautophagy (van Velthoven and Rando, 2019; Cho et al., 2019), herein referred to as autophagy. Epigenetic remodeling is another hallmark of quiescent stem cell states (van Velthoven and Rando, 2019; Cho et al., 2019; Hussein et al., 2020; Somasundaram et al., 2020; Hu et al., 2020). However, it remains unknown whether there are overarching rules that control entering and exiting quiescence across different types of stem cells. It will be particularly important to identify the molecules that distinguish reversible quiescence from DNA damage-induced apoptosis (Artoni et al., 2017; Xing et al., 2015).

**Figure 1:**
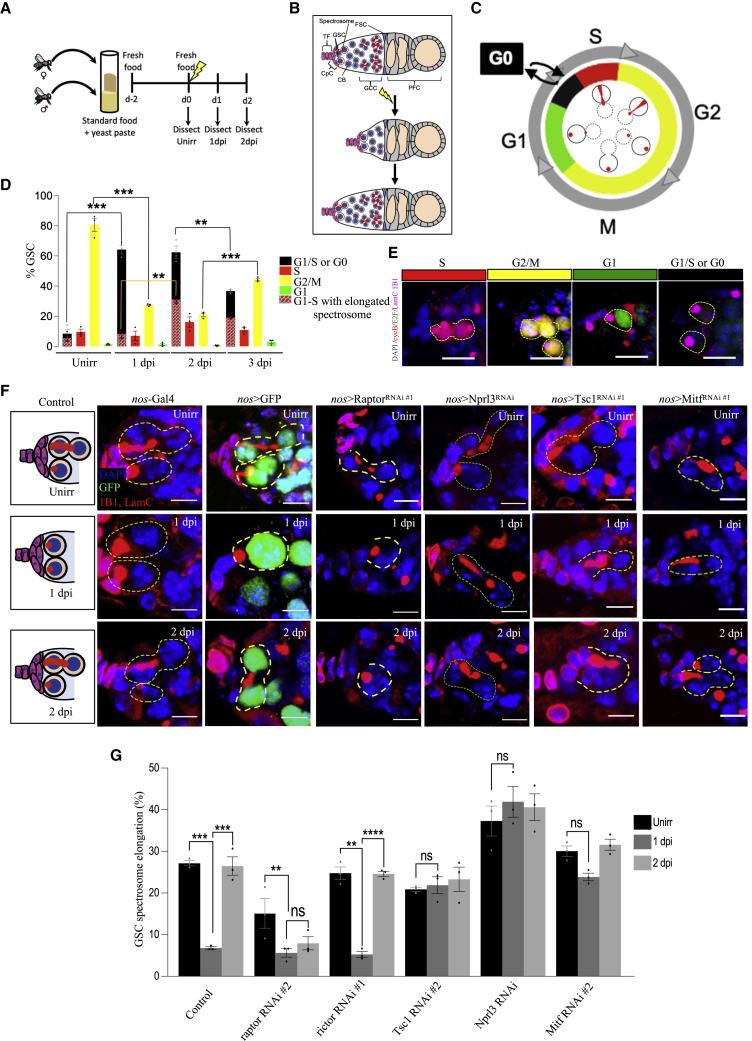
Role of mTORC1 in regulating insult-induced quiescence in female GSCs (A) Experimental setup for irradiation model. (B) Representative diagram of a germarium in the *Drosophila* ovary before and after IR. TF, terminal filament cell; CpC, cap cell; GSC, germline stem cell; CB, undifferentiated cystoblast; FSC, follicle stem cell; PFC, prefollicle cell. (C) Schematic of various cell-cycle stages of GSCs, correlated with the expression pattern of GFP-E2F1_1–230_ and mRFP-CycB_1–266_ in GSCs. (D) Percentage of cells positive for mRFP-CycB_1–266_ (red) or GFP-E2F1_1–230_ (green), dual positive mRFP-CycB_1–266_ GFP-E2F1_1–230_ (yellow), or expressing none of the fluorophore (black) under unirradiated, 1-day post-insult (1 dpi), 2 dpi, and 3 dpi conditions. Black represents G1/S or G0 phase, red represents S phase, yellow represents G2/M stage, and green represents G1 stage, while within the black bar the red dotted bar represents a percentage of cells in G1/S phase only, as these are the cells with elongated spectrosome. (E) Representative images of GSCs in the fly FUCCI line expressing GFP-E2F1_1–230_ and mRFP-CycB_1–266_. Dotted circle represents GSC (scale bar, 5 μm). (F) Representative confocal microscopy images of control (*nos*-Gal4 and *nos*>GFP) and listed UAS-RNAi KD from unirradiated, 1 dpi, and 2 dpi germaria stained with 1B1 (red, spectrosomes/fusomes), LamC (red, CpC and TF), and DAPI (blue, nuclei), as well as GFP (green, GSCs and progeny). Dotted circle represents GSC (scale bar, 5 μm). (G) Percentage of GSCs showing spectrosome elongation.∗p ≤ 0.05, ∗∗p ≤ 0.01, and ∗∗∗p ≤ 0.001.

DNA damage checkpoint recognizes irreparable DNA damage, inducing p53-dependent apoptosis (Aubrey et al., 2018; Speidel, 2010; Chakravarti et al., 2022; He et al., 2019). However, despite p53 activity (Hussein et al., 2020; Ma et al., 2016), stem cells in quiescence, including embryonic stem cells (ESCs) in diapause, resist apoptosis (Artoni et al., 2017; Hussein et al., 2020, 2022; Arena et al., 2021; Xing et al., 2015; Ma et al., 2016). Although it is not understood mechanistically how stem cells in general avoid apoptosis, in the germline stem cells (GSCs) of the adult *Drosophila* ovary at least one protective mechanism has been identified. GSCs in the somatic niche undergo self-renewing divisions to produce a cystoblast (CB) and a new GSC. The cystoblast further undergoes four incomplete cell divisions and eventually produces one oocyte connected to support cells called nurse cells; therefore, the GSCs are maintained in the somatic niche of the germaria to regenerate the oocyte pool. GSCs can survive genotoxic stress such as ionizing radiation (IR) by entering a reversible state of quiescence. In contrast, the GSC differentiating progenies undergo apoptosis and support survival of the GSCs (Xing et al., 2015). This reversible, protective GSC halt of the cell cycle allows regeneration of the germ line after insult (Artoni et al., 2017; Xing et al., 2015; Ma et al., 2016; Ishibashi et al., 2020, 2021) (Figures 1A and 1B). The stress-response transcription factor FOXO and the metabolic kinase mTOR were shown to be crucial for the entry into and exit from quiescence, respectively (Artoni et al., 2017). However, it is not yet known which mTOR targets are critical for the regulation of quiescence or how cell-cycle re-entry from GSC quiescence is so precisely timed.

In this study, we show that mTOR activity regulates the key epigenetic modifiers, PRC1/2, necessary for insult-induced mitophagy that results in quiescence. We further show that the mechanism of insult-induced quiescence relies on mitochondrial dynamics to temporally regulate a mitochondrial pool of cyclin E (CycE). Not only *Drosophila* GSCs, but also human induced pluripotent stem cells (hiPSCs) couple cell-cycle progression to mitochondrial quantity via the mitochondrial reserve of CycE.

## Results

### mTORC1 inhibition is necessary for IR-induced entry into quiescence in *Drosophila* GSCs

To dissect the GSC reversible cell-cycle block in more detail we employed the IR-induced insult paradigm and fly fluorescent ubiquitination-based cell-cycle indicator (FUCCI) to visualize distinct cell-cycle stages in GSCs (Zielke et al., 2014) (Figures 1C–1E). In the fly FUCCI line GFP-E2F1_1–230_ is degraded by ubiquitin ligase CRL4^Cdt2^ in S phase, and mRFP-CycB_1–266_ is degraded by APC/C in late mitosis/G1 (Figures 1C and 1E) (Zielke et al., 2014; Villa-Fombuena et al., 2021). Hence, FUCCI cells are green (GFP^+^/RFP^−^) in G1, red (GFP^−^/RFP^+^) in S, yellow (GFP^+^/RFP^+^) in G2/M, and black (GFP^−^/RFP^−^) in the G1-S transition (Figures 1C–1E). In the unirradiated control we observed a distribution of GSCs in (80%) G2/M, (8%) S, (1%) G1, and (8%) G1-S transition (Figures 1C and 1D), similar to previous findings (Villa-Fombuena et al., 2021). In contrast, 1 day post-insult (1 dpi) shows a significant increase in black GSCs (60%) (Figure 1D), a state of G0 quiescence. Further, 3 dpi shows a significant reduction in G0 with significant increase in G2/M (Figure 1D). At 2 dpi, when GSCs expectedly exit quiescence, we observe no significant reduction in black GSCs (Figure 1D). Upon analysis of the division marker phosphorylated serine 10 residue on histone 3 (PH3^+^) in GSCs, we found that 2.3%, 0.3%, and 1.9% of GSCs are PH3^+^ when unirradiated and at 1 and 2 dpi, respectively (Figure S1C), suggesting reversion of quiescence by 2 dpi (Figures S1B and S1C). To reconcile, we further analyzed another cell-cycle marker, GSC spectrosome elongation (Villa-Fombuena et al., 2021). The unirradiated GSCs showed an elongated spectrosome in S phase (or early G2) (Villa-Fombuena et al., 2021) (Figures 1C and 1F). At 1 dpi GSCs showed a significant reduction in elongated spectrosomes, whereas at 2 dpi the normal percentage of elongated spectrosomes was observed again, indicating the exit from quiescence (Figure 1F). Therefore, we proceeded to use elongated spectrosomes as a proxy for GSC division (Figures 1C, 1F, and 1G) (Artoni et al., 2017; Xing et al., 2015). We further categorized black GSCs in the FUCCI line into (1) unelongated, (2) elongated, or (3) partially elongated spectrosome type. When unirradiated, the rare black GSCs mainly showed partially elongated spectrosomes. At 1 dpi, black GSCs commonly have unelongated spectrosomes (75%) and partially elongated (25%) spectrosomes. Strikingly, at 2 dpi, we observed a significant increase in elongated spectrosomes, to 50% of black GSCs (Figures 1D and 1E), indicating G1-S transition (Figure 1C; Villa-Fombuena et al., 2021); hence, the GSCs appear to have mostly exited G0 or quiescence.

We sought to dissect the molecular role of the mTOR and its components to regulate quiescence. In wild-type (WT) control flies (*nos*-Gal4 driver), 27% of GSCs are in S phase with elongated spectrosomes (ESs; Figures 1F and 1G). At 1 dpi, the GSCs enter a state of quiescence (5% ES), and at 2 dpi GSCs successfully exit quiescence and resume cell division (27% ES; Figures 1F and 1G). Knockdown (KD) of the mTORC1 component raptor by RNAi moderately decreases rates of GSC division (12% ES) (Figures 1F and 1G), consistent with mTORC1 activity promoting cell division (Artoni et al., 2017). At 1 dpi the raptor KD GSCs efficiently enter IR-induced quiescence (2% ES), but strikingly, GSC division remains low at 2 dpi (3% ES), indicating a failure to exit quiescence (Figures 1F and 1G). In contrast, KD of the mTORC2 component rictor by RNAi has no appreciable effect on unirradiated rates of division (25% ES), and the GSCs properly arrest division at 1 dpi (5% ES) and resume division at 2 dpi (25% ES) (Figures 1G and S1A and Table S1), similar to WT controls, suggesting that mTORC2 might be functionally dispensable in GSC insult-induced quiescence. Altogether, these data show that mTORC1 activation is necessary for the exit from quiescence.

mTOR regulatory complexes TSC and GATOR1 are known to inhibit mTOR activity, while GATOR2 inhibits GATOR1 and can thereby activate mTORC1 (Kim and Guan, 2019; Wei et al., 2019). When the TSC component Tsc1 was knocked down by RNAi in GSCs, the rate of GSC division remained unchanged (unirradiated, 22% ES; 1 dpi, 21% ES; 2 dpi, 24% ES) (Figures 1F and 1G), suggesting that Tsc1 is required for quiescence. Similarly, when the GATOR1 component Nprl3 is knocked down, the GSCs do not block the cell cycle after insult (unirradiated, 37.5% ES; 1 dpi, 44.5% ES; 2 dpi, 43.5% ES) (Figures 1F and 1G). KD of another GATOR1 component, Nprl2, showed a similar inability to arrest cell division (Table S1), suggesting that GATOR1-mediated mTORC1 inhibition is essential for insult-induced quiescence. Intriguingly, GSCs with GATOR2 component (Nup44a and Mio) KD are comparable to WT controls (Table S1). These data are consistent with earlier findings that both TSC and GATOR1 become activated in response to the programmed DNA double-strand break during meiosis to mitigate genotoxic stress (Wei et al., 2019), and that GATOR2 is not appreciably active in GSCs (Wei et al., 2014). In conclusion, mTORC1 repression is essential to enter insult-induced quiescence in GSC.

During stress, mTORC1 inactivation results in transcription factor MITF/TFEB/TFE3 dephosphorylation, resulting in its nuclear translocation (Settembre et al., 2011). In *Drosophila* Mitf is the single identified MITF-TFE family member. Mitf can regulate V-ATPase expression that results in amino-acid-dependent activation of mTOR. mTORC1 then can phosphorylate and sequester *Drosophila* Mitf in the cytoplasm, leading to Mitf transcriptional inactivation through this feedback loop (Zhang et al., 2015). In addition, mammalian Mitf is shown to act in a multitude of other biological processes (Goding and Arnheiter, 2019). We sought to test the role of Mitf in *Drosophila* GSC IR-induced quiescence. Mitf KD abrogates insult-induced quiescence, as division rates at 1 dpi remain high (Figures 1F and 1G), suggesting that mTORC1 regulates GSC quiescence via Mitf-transcriptional targets, such as autophagy genes (Bouché et al., 2016).

### Autophagy-deficient GSCs fail to enter into quiescence

**Figure 2:**
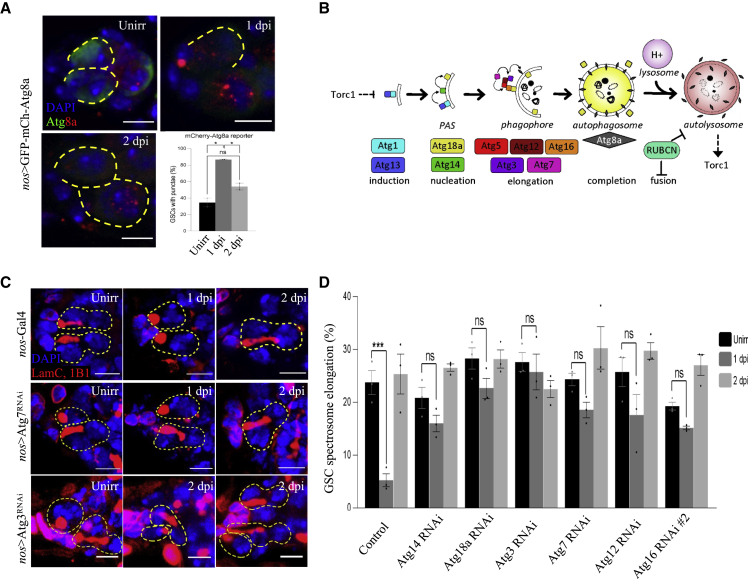
Autophagy-defective germline stem cells display impaired quiescence (A) Representative confocal microscopy images of *nos*>GFP-mCherry-Atg8a from unirradiated, 1 dpi, and 2 dpi germaria stained with GFP (green, cytoplasm/autophagosome), mCherry (red, autophagosome/autolysosome), and DAPI (blue, nuclei). Dotted circle represents GSC (scale bars, 5 μm). The graph shows quantification of puncta in *nos*>mCherry-Atg8a germaria, stained with mCherry. A portion of unirradiated GSCs (35%) contain >1 mCherry puncta. At 1 dpi, this increases sharply (87%), suggesting an acute increase in autophagic degradation. By 2 dpi, GSCs with puncta decrease (54%), concurrent with the exit from quiescence. (B) Schematic of known and hypothesized elements of interplay between autophagy and mTORC1. (C) Immunofluorescence images of GSCs with respective overexpression or knockout of core components of autophagy proteins stained with 1B1 (red, spectrosomes/fusomes), LamC (red, CpCs and TFs), and DAPI (blue, nuclei). Dotted circle represents GSC (scale bars, 5 μm). (D) Percentage of GSCs showing spectrosome elongation. ∗p ≤ 0.05 and ∗∗∗p ≤ 0.001.

We analyzed if mTORC1/Mitf-dependent autophagy regulates GSC quiescence. To characterize autophagy upon irradiation, we used the *nos*>mCherry-Atg8a (a reporter of autophagosome/autolysosome formation) line (Mauvezin et al., 2014). We observed a basal level of autophagy before irradiation, where 35% of GSCs contain mCherry-Atg8a puncta (Figures 2A and S2A–S2C). The number of GSCs with puncta increased at 1 dpi (87%) and reduced at 2 dpi (54%) (Figures 2A and S2A–S2C), indicating that autophagosomes/autolysosomes are acutely upregulated at GSC quiescence. To further characterize the rate of autophagic flux, we analyzed *nos*>GFP-mCherry-Atg8a GSCs. Unlike mCherry fluorescence, GFP is highly sensitive to pH, and therefore quenches in the low pH of the autolysosome (Mauvezin et al., 2014). Using this reporter, autophagosomes are expected to be yellow (GFP^+^/mCherry^+^), while autolysosomes are expected to be red (GFP^−^/mCherry^+^) (Figure S2A). Interestingly, we observed only red puncta, suggesting that, in GSCs, mature autophagosomes are rapidly acidified, characteristic of high autophagic flux (Figures 2A, 2B, and S2C). These data align with past results in hematopoietic stem cells suggesting that quiescence is characterized, in part, by the accumulation of autolysosomes (Liang et al., 2020).

Autophagy activation by either overexpression of Atg1, an inducer of autophagy, (Figures S2D and S2E), or KD of Rubicon, an inhibitor of autophagy (Table S1), prevented complete cell-cycle re-entry at 2 dpi, suggesting that dampening of autophagy is required to exit from quiescence. Atg1 has previously been reported to be phosphorylated by CDK1/cyclin B and is necessary for cell-cycle progression, which potentially explains the high basal division rate at 1 and 2 dpi in Atg1-overexpressing GSCs. Conversely, depletion of the core autophagy genes involved in phagophore nucleation, autophagic membrane elongation, or autophagosome maturation impaired the entrance into quiescence (Atg14, 18a, 3, 7, 12, 16; Figures 2C, 2D, and S2D and Table S1). These data align with findings that mouse ESCs arrest cell division upon chemical induction of autophagy (Suvorova et al., 2019), suggesting autophagy may be a more universal regulator of stem cell state.

### Mitophagy acts downstream of IR-induced autophagy

**Figure 3:**
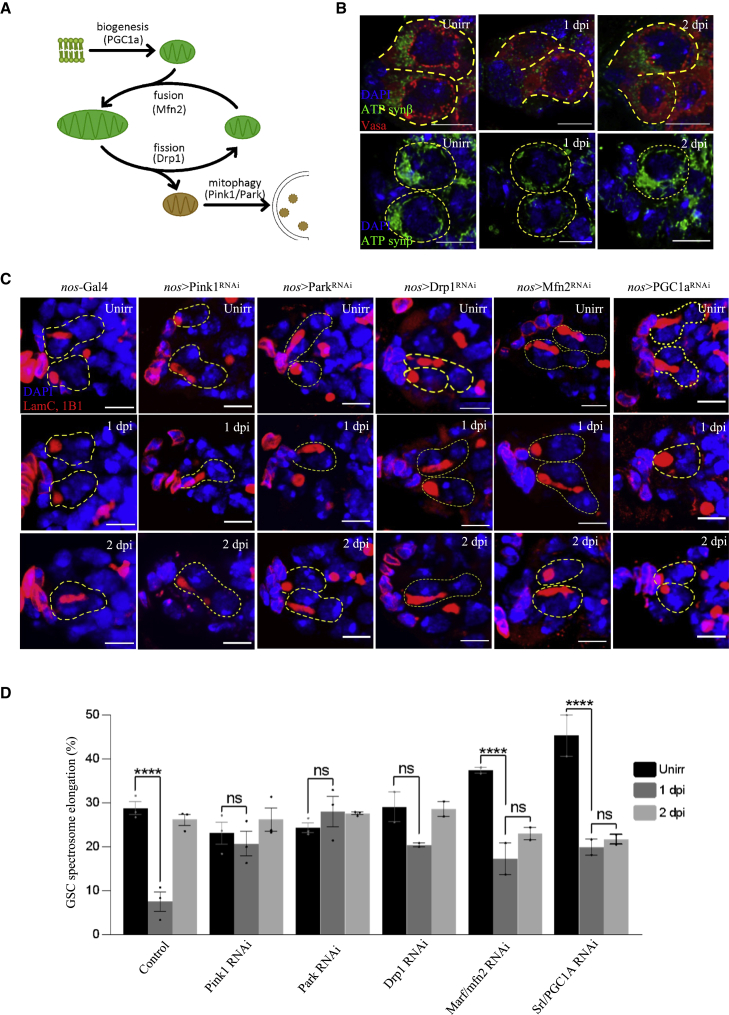
Mitochondrial remodeling events are required for proper coordination of quiescence (A) Current model of mitochondria life cycle based on current literature. (B) Representative confocal microscopy images of control GSCs from unirradiated, 1 dpi, and 2 dpi germaria stained with VASA (red), ATP synthase β subunit (ATPsynβ) (green, mitochondria), and DAPI (blue, nuclei) (top) or only ATPsynβ and DAPI (bottom). Scale bars, 5 μm. (C) Representative confocal microscopy images of RNAi KD of mitochondrial fission (Drp1) and mitophagy (Pink1/Park) and mitochondrial fusion (Mfn2) and biogenesis (PGC1α) genes from unirradiated, 1 dpi, and 2 dpi germaria stained with 1B1 (red, spectrosomes/fusomes), LamC (red, CpCs and TFs), and DAPI (blue, nuclei). Dotted circle represents GSC (scale bars, 5 μm). (D) Percentage of GSCs showing spectrosome elongation. ∗∗∗∗p ≤ 0.0001.

Mitochondrial autophagy (mitophagy) and mitochondrial remodeling are important cellular processes that, when defective, can result in disease states (Sun et al., 2016). We tested if the mTOR pathway in GSCs has the capacity to control mitophagy, as seen in another context (de la Cruz López et al., 2019). While the mitochondrial network of unirradiated WT GSCs was relatively fused and reticular, with increased mitochondrial density at the anterior end of the GSCs (Figures 3B and 5F) (Lieber et al., 2019; Cox and Spradling, 2003; Wang et al., 2016), at 1 dpi, most of the mitochondria were abnormal, 29% of GSCs with significantly decreased mitochondrial content (Figures 3B, 5A, 5F, and 5G). This decrease in mitochondrial content is dependent on autophagy, since Atg3 mutant GSCs display a normal anterior mitochondrial pattern at 1 dpi (Figures 5F, 5G, and S3M). Finally, at 2 dpi, the mitochondria returned to a more fused, anterior network, like the mitochondria of unirradiated GSCs (Figures 3B, 5A, 5F, and 5G). We show that Tsc1 RNAi mutant GSCs that lack quiescence (Figures 1F and 1G) also lack mitochondrial reduction at 1 dpi (Figures 5F, 5G, and S3L). These data reveal that GSC mitochondrial degradation and biogenesis coincide with mTOR-dependent entry into and exit from quiescence. This finding is strikingly like that in yeast, wherein proliferating yeast cells contain a tubular meshwork of mitochondria, while quiescent yeast cells have peripheral, fragmented mitochondria (Laporte et al., 2018), suggesting that mitochondrial morphology controls and/or responds to cell proliferation. We tested this hypothesis in stem cells by analyzing IR-induced quiescence in KD of the *Drosophila* mitophagy proteins, Pink1 and Parkin.

**Figure 4:**
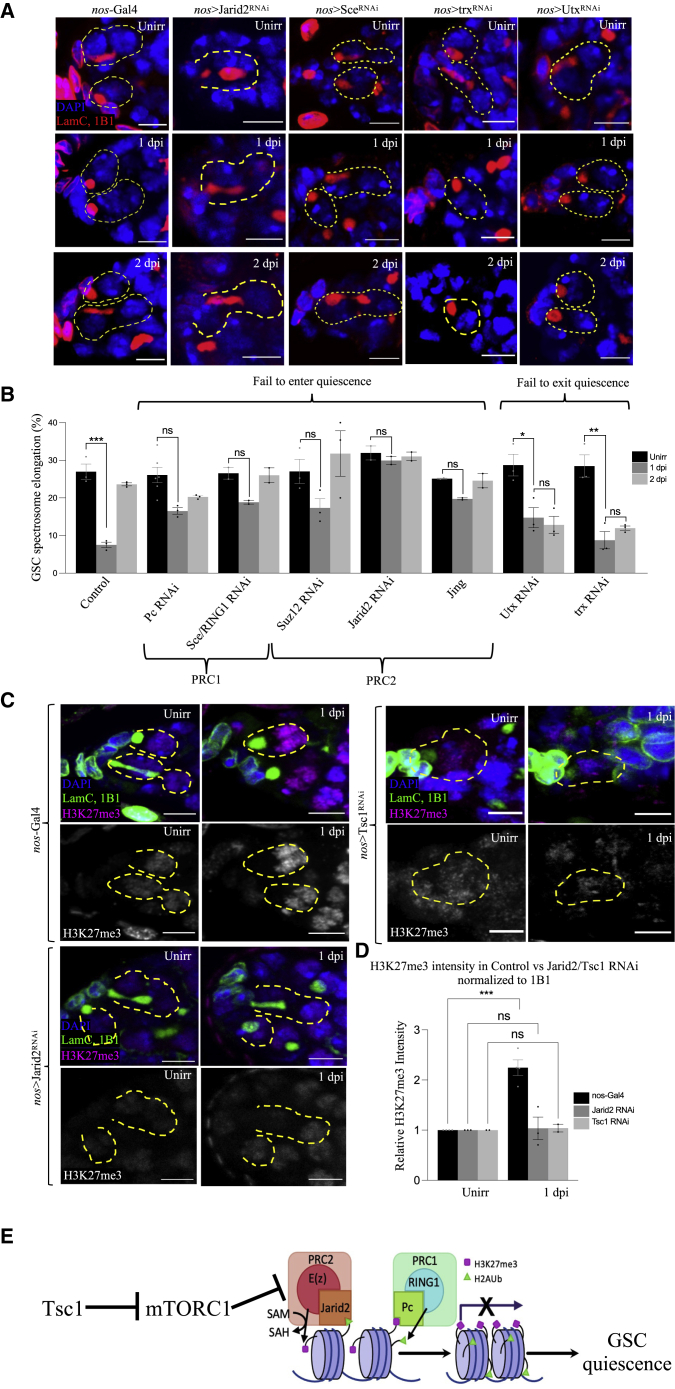
Epigenetic proteins also regulate GSC quiescence (A) Representative confocal microscopy images of RNAi KD of various epigenetic regulatory genes from unirradiated, 1 dpi, and 2 dpi germaria stained with 1B1 (red, spectrosomes/fusomes), LamC (red, CpCs and TFs), and DAPI (blue, nuclei). Dotted circle represents GSC (scale bars, 5 μm). (B) Percentage of GSCs showing spectrosome elongation. (C) Representative confocal microscopy images of control, Jarid2-RNAi, and Tsc1-RNAi GSCs from unirradiated, 1 dpi, and 2 dpi germaria stained with LamC (green, CpCs and TFs), 1B1 (green, spectrosomes/fusomes), H3K27me3 (magenta), and DAPI (blue, nuclei). Dotted circle represents GSC (scale bars, 5 μm). (D) Quantification of relative fluorescence intensity of H3K27me3 normalized to 1B1 intensity in control GSCs and Jarid2 RNAi GSCs, unirradiated and at 1 dpi. (E) Upon irradiation, the canonical roles of PRC1 and PRC2 in epigenetic regulation during quiescence are shown. PRC2 binds to DNA by Jarid2, while E(z) consumes SAM to methylate H3K27, leading to transcriptional repression. PRC1 then binds to and recognizes existing H3K27me3 marks through CBX, and then catalyzes monoubiquitination of H2A, which further represses transcription and hence leads to quiescence. ∗p ≤ 0.05, ∗∗p ≤ 0.01, and ∗∗∗p ≤ 0.001.

**Figure 5:**
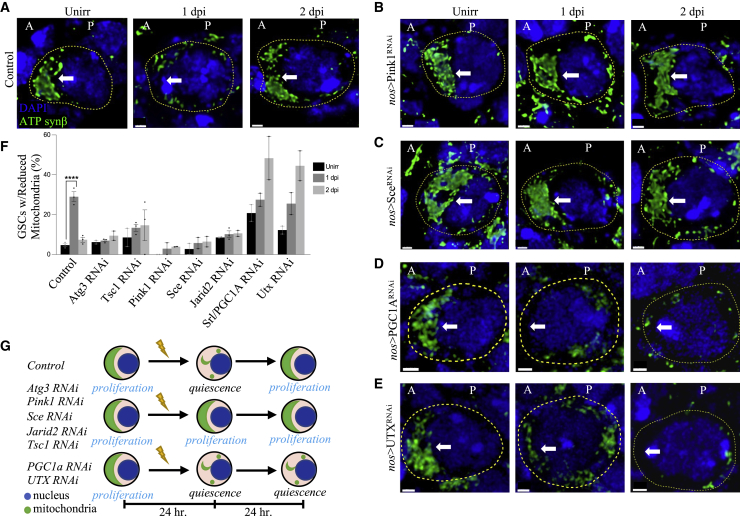
Mitophagy-dependent quiescence is mediated by PRC1 (A–E) Representative 3D reconstructed confocal microscopy images of GSCs and their respective RNAi KD line when unirradiated and at 1 and 2 dpi, stained with DAPI (blue, nuclei) and ATPsynβ (green, mitochondria) (“A” denotes anterior side, and “P” denotes the posterior side of the GSC). Arrow points to the area of interest, where mitochondria are typically clustered (scale bars, 1 μm). (F) Quantification of incidence of reduced mitochondria where clustered mitochondria are not present in GSCs. Images from Figures S3K–S3M were used to generate this quantification. (G) Proposed model by which mitochondria population acts as a checkpoint for the cell-cycle state of GSC. Blue represents nucleus and green represents mitochondria. ∗∗∗∗p ≤ 0.0001.

The Parkinson’s disease genes Pink1 and Parkin mediate mitochondrial quality control mechanisms that culminate in the clearance of depolarized or dysfunctional mitochondria (Narendra et al., 2008; Matsuda et al., 2010; Vives-Bauza and Przedborski, 2010). We performed Pink1 and Parkin RNAi KD and found that the GSCs lacking Pink1 did not undergo mitochondrial degradation observed with WT GSCs at 1 dpi (Figures 5B, 5F, and 5G), arguing that mitochondria in normal GSCs after insult undergo mitophagy that requires Pink1 kinase activity. Furthermore, while the unirradiated Pink1 and Parkin KD GSCs divide at rates comparable to those of their WT counterparts (Figures 3C and 3D), Pink1 and Parkin KD GSCs still divide at 1 dpi (21% and 28%), failing to enter quiescence (Figures 3C and 3D). Using *nos*>mCherry-Atg8a, we further showed that Pink1-mediated mitophagy was downstream of IR-induced autophagy in GSCs; although Pink1 KD GSCs lacked mitophagy at 1 dpi, they still contained a significant increase in red puncta (Figure S3N). Our findings demonstrate that Pink1 and Parkin-mediated mitophagy is required for GSC IR-induced quiescence.

We also analyzed the canonical regulators of mitochondrial dynamics in GSC quiescence, which are known to interact with the mitophagy receptors Pink1/Parkin (Poole et al., 2010; Deng et al., 2008a, 2008b). We first analyzed the GTPase Dynamin-related protein, Drp1, which forms helical oligomers to pinch around the outer mitochondrial membrane and induce fission. Drp1 KD GSCs divided normally (29%) before irradiation but exhibited defects in arresting division at 1 dpi (20.5%) (Figures 3C and 3D), which implicates the role of mitofission (Figure 3A) in the entry into quiescence. Further, depletion of Marf/Mfn2, a GTPase responsible for mitochondrial fusion, showed a higher than normal division rate in unirradiated GSCs, and a reduced division at 1 dpi (>2-fold reduction), but failed to increase division by 2 dpi (Figures 3C and 3D). Similar to Mfn2 KD, mitochondrial biogenesis regulator PGC1α/srl KD GSCs also divided more than WT controls and significantly reduced division at 1 dpi (>2-fold reduction), but failed to increase division at 2 dpi (Figures 3C and 3D). These data show that PGC1α/srl KD GSCs undergo normal mitochondrial degradation at 1 dpi but fail to return to a more normal anterior accumulation of mitochondria at 2 dpi (Figures 5D, 5F, and 5G). In line with these data, in the fly FUCCI line, the black GSCs marked for quiescence at 1 dpi showed a significant reduction in mitochondria (Figure S1F). These data show that in GSCs, mitochondrial fission and mitophagy are required for the entry into quiescence, and mitochondrial fusion and biogenesis are required for the exit from quiescence.

### Epigenetic modifiers are required for GSC entry into and exit from quiescence

The epigenome has been previously shown to be regulated by mitochondrial metabolites, and epigenomic changes have been identified in quiescent stem cell states (van Velthoven and Rando, 2019; Cho et al., 2019; Hussein et al., 2020; Somasundaram et al., 2020; Hu et al., 2020; Martínez-Reyes and Chandel, 2020; Moody et al., 2017; Levy et al., 2022). We now show that KD of the PRC1 (Pc/CBX and Sce/RING1) or PRC2 (Jarid2, Su(z)12, E(z), and Jing) components in GSCs abolishes the normal IR-induced cell-cycle block at 1 dpi, suggesting that these repressive epigenomic modifiers are required for entry into GSC quiescence (Figures 4A, 4B, and S3). Interestingly, some transcriptionally activating epigenetic modifiers were also required for quiescence entry, such as H3K10 kinase, Jil1(Deng et al., 2008a, 2008b); H3K79 methyltransferase, Gpp/DOT1L; and H3K4me2/3 methyltransferase, Set1 (Hallson et al., 2012) (Figures S3A–S3J and Table S1), suggesting that quiescence requires specific transcriptionally activating modifications. Additional enzymes responsible for recognizing DNA damage, γ-H2Av kinase and mei-41/ATR, are required for quiescence (Figures S3C and S3J and Table S1).

Conversely, we found another class of epigenetic modifiers that seem to be essential for the exit from quiescence. RNAi KD of Utx, an H3K27me3 demethylase, prevents GSCs from exiting quiescence (Figures 4A and 4B). An additional histone modifier, H3K4me1 methyltransferase Trx (Tie et al., 2014), also regulates GSC exit from quiescence (Figures 4A and 4B). Curiously, rhi, a piRNA pathway component and member of the heterochromatin protein 1 (HP1) family (Klattenhoff et al., 2009), likely regulates various aspects of GSC homeostasis and quiescence, as rhi RNAi KD causes low baseline division before irradiation and impairs quiescence entry and exit at 1 and 2 dpi, respectively (Figures S3B and S3J and Table S1). We confirmed PRC1/2 activity in WT 1 dpi GSC nuclei by detecting a significant increase in H3K27me3 intensity compared with unirradiated nuclei (Figures 4C and 4D). In contrast, KD of the PRC2 component Jarid2, or mTOR regulator Tsc1, showed no significant difference in H3K27me3 levels at 1 dpi (Figures 4C and 4D). These data show that mTOR acts upstream of PRC1/PRC2 epigenetic modifiers that are required for GSC quiescence (Figure 4E).

### Mitophagy-dependent quiescence is under dynamic epigenetic control

To test the relationship between mTOR, mitophagy, and epigenetic control of GSC IR-induced quiescence, we stained GSCs with ATPsynβ to analyze mitochondrial pattern and morphology in Tsc1, Pink1, Sce, PGC1/srl, and Utx RNAi mutants (Figures 5A–5F and S3K–S3L). As discussed above, at 1 dpi, the anterior mitochondrial network is lost in most of the WT GSCs, and the GSC ratio with dramatically reduced mitochondria is significantly increased. Using the fly FUCCI system we show that the observed mitochondrial reduction after insult occurs during the G0 stage of cell cycle (Figure S1F). The mitochondria morphology and pattern at 2 dpi are similar to those of unirradiated, representing exit from quiescence (Figures 5A, 5F, 5G, and S3K). In contrast, Tsc1 and Pink1 KD GSCs, which fail to enter quiescence after insult (Figures 1F, 1G, 3C, and 3D) showed the fewest changes in the mitochondrial network at 1 dpi (3%) (Figures 5B, 5F, S3K, and S3L), consistent with mTOR action upstream of mitophagy. Interestingly, epigenetic regulator Sce/RING1 KD GSCs, which fail to enter quiescence after insult (Figures 4A and 4B), also display low mitochondrial reduction (6%) at 1 dpi (Figures 5C, 5F, and S3K). Furthermore, Jarid2 RNAi mutants failed to detect IR-induced autophagy (Figure S3N). In contrast, PGC1 and Utx RNAi mutants continued to display segmented and highly reduced mitochondria at 2 dpi (Figures 5D–5F). These data suggest that PRC1/PRC2 and H3K27me3 demethylation are required for mitochondrial regulated quiescence and that the PRC1/PRC2-based epigenetic modifications act upstream of the mitophagy (Figure 5G).

### Cyclin E localized to the mitochondria is degraded upon genotoxic or chemical insult in GSCs and hiPSCs

Since female *Drosophila* GSC division does not rely heavily on mitochondrial ATP (Teixeira et al., 2015), we tested the hypothesis that the mitochondria play a more direct role in cell-cycle progression. CycE, a G1-S regulator, has been reported to be targeted for degradation by ubiquitination by Parkin (Staropoli et al., 2003), the E3 ubiquitin ligase required for mitophagy and GSC quiescence (Figures 3C and 3D). Furthermore, Parkin mutations are associated with increased CycE in both cancer and Parkinson’s neurons (Veeriah et al., 2010). This Parkin ubiquitination activity on CycE is regulated by a serine/threonine kinase, Pink1 (Ejma et al., 2020). While Parkin is involved in mitophagy and ubiquitin-dependent degradation of CycE, the connection between them remains unclear. Since recent work has observed some CycE co-localization with the mitochondria in both flies and mammals (Parker et al., 2015; Spurlock et al., 2020), we tested the hypothesis that Pink1-activated Parkin will be localized to mitochondria, where it initiates CycE degradation and mitophagy, resulting in quiescence.

**Figure 6:**
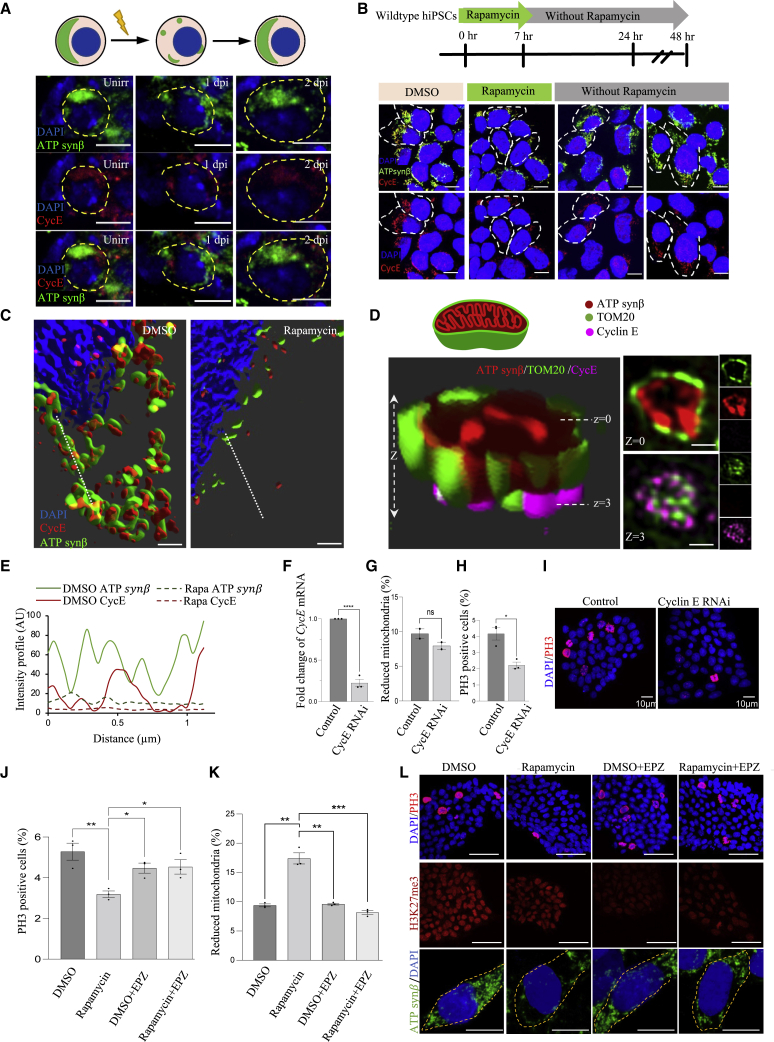
Pool of cyclin E is observed on mitochondria in GSCs and hiPSCs (A) Representative confocal microscopy images of *Drosophila* GSCs from unirradiated, 1 dpi, and 2 dpi germaria stained with ATPsynβ (mitochondria, green), cyclin E (red), and DAPI (blue) with cartoon schematic above (scale bars, 5 μm). (B) Model shows the experimental design. Representative confocal microscopy images of WT hiPSCs treated with either vehicle control (DMSO) or rapamycin (2 μM) and pulse-chase stained with ATPsynβ (mitochondria, green), cyclin E (red), and DAPI (blue) (scale bars, 10 μm). hiPSCs shows reduction in both mitochondrial and cyclin E density compared with vehicle control after 7 h of 2 μM rapamycin treatment. Both mitochondria and cyclin E densities increase after 24 and 48 h post rapamycin treatment. Gray dashes represent the boundary of the cells of interest. (C) Representative 3D-reconstructed OMX super-resolution microscopy images of WTC cells treated with vehicle control (DMSO) or 2 μM rapamycin for 24 h and stained with ATPsynβ (green), cyclin E (red), and DAPI (blue). The white dotted line was used to quantify the intensity profile in (E) (scale bars, 0.5 μm). (D) A super-resolution 3D reconstruction of a mitochondrion in Tom20-GFP-expressing WTC, stained for ATPsynβ (red), GFP (green), and cyclin E (magenta). On the right, the two sections z = 0 and z = 3 show the mid-section and bottom of the mitochondrion (scale bars, 0.5 μm). (E) Intensity profile of ATPsynβ (green) and CycE (red) in DMSO (solid line) and rapamycin (dotted line). The images in (C) were used to quantify the plot profile. (F) Fold change of CycE analyzed in qPCR after knocking down CycE with siRNA for 72 h. (G) Percentage of cells with reduced mitochondria in control or CycE siRNA for 72 h, n = 3, number of cells analyzed per condition >500. (H) Percentage of cells positive for PH3 in control or CycE siRNA for 72 h, n = 3, number of cells analyzed per condition >500. (I) Representative confocal microscopy images of wild-type hiPSCs transfected with either control or cyclin E siRNA for 72 h. DAPI is blue, PH3 is red (scale bars, 10 μm). (J) Percentage of cells positive for PH3 in DMSO, 2 μM rapamycin, EPZ-6438, and rapamycin + EPZ-6438 for 24 h, n = 3, number of cells analyzed per condition >1,000. (K) Percentage of cells with reduced mitochondria in DMSO, 2 μM rapamycin, EPZ-6438, and rapamycin + EPZ-6438 for 24 h, n = 3, number of cells analyzed per condition >500. (L) Representative confocal images of cells treated with DMSO, 2 μM rapamycin, EPZ-6438, and rapamycin + EPZ-6438 for 24 h. Cells were stained for DAPI (blue), PH3 (red, scale bar, 50 μm), ATPsynβ (green, scale bar, 10 μm), and H3K27Me3 (red,scale bars, 50 μm). (B–E) wild type, WTC. (F–L) control, WTC-Tom20. ∗p ≤ 0.05, ∗∗p ≤ 0.01, ∗∗∗p ≤ 0.001, and ∗∗∗∗p ≤ 0.0001.

In *Drosophila*, unirradiated GSCs show striking co-localization of CycE with the anteriorly localized, fused mitochondrial network (Figure 6A; Lieber et al., 2019; Cox and Spradling, 2003). However, at 1 dpi, the GSCs show a dramatic loss of quantity and anterior localization of both CycE and mitochondria, while at 2 dpi, CycE is once again observed co-localizing with the mitochondria, which are fused and anteriorly localized (Figures 6A and S3P). These data confirm that the mitochondria likely harbor a pool of CycE in GSCs and support the hypothesis that the mitochondrial content dictates cell-cycle progression after insult by regulating the CycE availability for G1-S transition.

To further understand whether the mTOR-regulated, p21 independent, irradiation-induced *Drosophila* GSC quiescence is conserved in mammalian stem cells, we irradiated hiPSCs and analyzed if the process resulted in mTOR inhibition (Qi et al., 2009; Zhan et al., 2019). Previous work has shown that phosphorylation and nuclear exit of the transcription factor TFE3 indicate mTOR activity in hiPSCs (Figure S4G; Mathieu et al., 2019). We therefore analyzed TFE3 nuclear localization in hiPSCs before and after irradiation (0.52 Gy of gamma-irradiation). As expected, TFE3 was mainly observed in the cytoplasm in control hiPSCs (Figures S4B and S4C). However, irradiation induced a 6-fold increase in nuclear TFE3 localization (1 dpi), suggesting that irradiation inhibits mTOR (Figure S4B). Concomitantly, irradiation induced a significant reduction in mitochondria in 80% of the hiPSCs (Figure S4A). These data suggest that irradiation inactivates mTOR and induces mitophagy also in hiPSC. Rapamycin treatment (mTOR inhibition) has previously been shown to induce quiescence (a diapause-like state) in pluripotent stem cells (Hussein et al., 2020). We, therefore, treated the hiPSCs with rapamycin as a surrogate of irradiation-induced mTORC1 inhibition and analyzed the effect of mitochondrial dynamics on CycE levels in quiescent hiPSC. We confirmed that rapamycin inhibits mTORC1 in hiPSCs (reduced pS6 within 7 h of treatment; Figure S4E). We found a stronger, but reversible inhibition of mTORC1 if rapamycin treatment was extended to 24 h (pS6 signal returns in 3 days after mTORC1 reactivation (Figures S4E and S4F). Twenty-four hours of rapamycin treatment also promoted significant nuclear localization of TFE3, confirming rapamycin efficiency in our experimental setup (Figure S4G). Importantly, rapamycin treatment resulted in a reversible reduction of epigenetic H4K16Ac marks (Figures S4E and S4F), indicating reduced cellular transcription previously seen in ESC diapause/quiescence (Hussein et al., 2020). These data suggest that hiPSCs can enter reversible quiescence after mTOR inhibition by rapamycin. Importantly, we observed a dramatic co-localization of CycE and mitochondria also in hiPSCs (Figures S4D, S4H, and S4I). The rapamycin-dependent co-localization of CycE and mitochondria was also reversible: while 7 h rapamycin treatment drastically decreased the patterns, after 24–48 h recovery, the mitochondria and CycE co-localization was similar to that of the vehicle controls (Figure 6B). These data confirm that mTORC1 inhibition in hiPSCs degrades both mitochondria and CycE, in a reversible manner.

Using super-resolution microscopy, we observed distinct co-localization of CycE with an outer mitochondrial membrane import receptor subunit, Tom20 (in the same mitochondrial region), but not with ATPsynβ, suggesting that the mitochondrial pool of CycE resides on the outer mitochondrial membrane (OMM) (Figure 6D). Upon rapamycin treatment (2 μM, 24 h) the mitochondria degraded, with a concomitant reduction in OMM CycE levels (Figures 6C and 6D). The reduction of mitochondrial CycE is proteasome dependent, since it can be reversed by treatment with a proteasome inhibitor (MG132; however, this does not rule out lysosomal degradation) (Figure S4M). To further test if CycE is critical for quiescence in iPSCs, we knocked down CycE (72 h; Figure 6F) and observed a significant decrease in PH3-positive cells, without altering mitochondrial reduction (Figures 6G–6I). This further implies that a reduced level of CycE is directly associated with stem cell entry into quiescence in hiPSCs.

### Epigenetic control of hiPSC quiescence

When we treated hiPSCs with rapamycin to inhibit mTOR, and EPZ-6438 to inhibit EZH2, a component of the PRC2 complex, we confirmed PRC2 inhibition by a reduction in H3K27me3 (Figures 6L and S4K). In rapamycin treatment we observed a significant reduction in the number of PH3^+^ cells, showing that rapamycin can induce a quiescence-like cell-cycle block in hiPSCs (Figures 6J and 6L). Further, we found that co-treatment of rapamycin with EPZ-6438 (Figure S4J) prevented hiPSCs from entering mTOR inhibition-dependent quiescence (Figures 6J and 6L). These data suggest that PRC2-mediated gene repression is essential for quiescence in hiPSCs and acts downstream of mTOR regulation. The control hiPSCs showed a significant increase in mitochondrial reduction in rapamycin-treated samples, while co-treatment of rapamycin and EPZ-6438 showed a lack of mitochondrial reduction (Figures 6K and 6L). This suggests that the state of quiescence induced through mTORC1 inhibition in hiPSCs is mediated by repressive histone marks, H3K27me3, which are upstream of mitochondrial degradation/fragmentation.

### PINK1-mediated mitophagy dictates quiescence through cyclin E in hiPSCs

**Figure 7:**
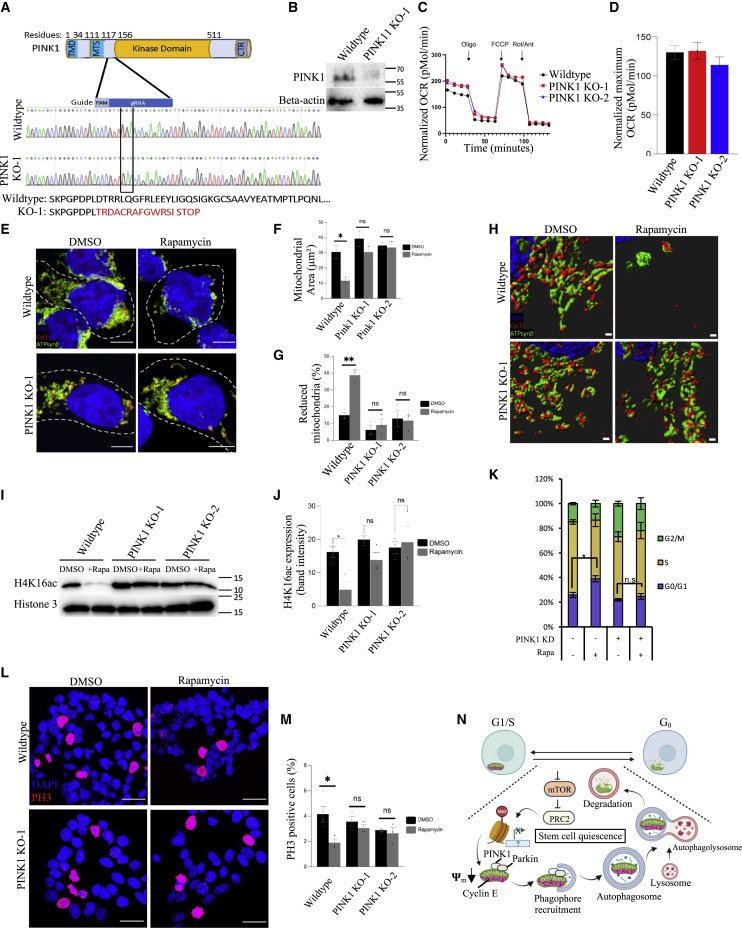
Mitophagy in hiPSCs controls both mitochondrial cyclin E and cell cycle (A) Pink1 structure with guide RNA location indicated and DNA sequencing chromatogram comparing wild-type Pink1 to mutant Pink1, showing a mixed pool of mutants and a loss of the wild-type sequence. (B) Western blot showing Pink1 protein knocked out. (C and D) Representative trace of OCR changes in response to oligomycin, FCCP, and rotenone/antimycin is shown under a MitoStress protocol (C). Pink1 KO cells show similar mitochondrial respiration pattern compared with wild-type Pink1 (D). (E) Representative confocal microscopy images of wild-type or Pink1 mutant cells treated with either vehicle control (DMSO) or rapamycin (2 μM) and stained with DAPI (blue), CycE (red), and ATPsynβ (mitochondria, green) (scale bars, 5 μm). (F and G) Quantification of (F) area of mitochondria and (G) mitochondrial degradation in wild type vs. Pink1 mutant treated with either vehicle control (DMSO) or rapamycin (2 μM). Following are the total numbers of cells quantified (n) for wild-type and Pink1-KO cells for DMSO- and rapamycin-treated cells, respectively: wild type (without cyclin E stain), 618, 518; wild type (with cyclin E stain), 512, 414; Pink1-KO cells (without cyclin E stain), 616, 701; wild type (with cyclin E stain), 502, 569. (H) Representative 3D reconstructed OMX super-resolution microscopy images of wild-type or Pink1 mutant cells stained with DAPI (blue), CycE (red), and ATPsynβ (mitochondria, green) (scale bars, 0.2 μm). (I) Representative western blot analysis of wild type, Pink1 KO-1, and Pink1 KO-2 treated with DMSO or 2 μM rapamycin for 24 h and blotted with H4K16Ac and histone 3 as a loading control. (J) H4K16Ac quantification of the western blot in (I). (K) Quantification of fluorescence-activated cell sorting (FACS) analysis of cell cycle by propidium iodide staining in wild type or Pink1KD treated with DMSO or rapamycin (2 μM). (L) Representative confocal microscopy images of wild-type or Pink1 mutant cells treated with either vehicle control (DMSO) or rapamycin (2 μM) and stained with DAPI (blue) and PH3 (proliferating cell nuclei, red) (scale bars, 15 μm). (M) Quantification of PH3 incidence in wild type vs. Pink1 mutant treated with either vehicle control (DMSO) or rapamycin (2 μM). Following are the total numbers of cells quantified (n) for wild type and Pink1-KO for DMSO- and rapamycin-treated cells, respectively: wild type, 3439, 2760; Pink1-KO, 3162, 3184. (N) Model of proposed mechanism in which mitochondria can normally stabilize CycE and promote G1-S transition, while mitophagy induction will reduce CycE—perhaps through direct ubiquitination by Parkin—and keep the stem cells in quiescence/G0. (A–J, M–L) Wild type, WTC-Tom20; (K) wild type, WTC. ∗p ≤ 0.05 and ∗∗p ≤ 0.01.

To further investigate the role of mitophagy in regulating the stability of CycE, we generated hiPSC lines with CRISPR-Cas9-induced mutations in the mitochondrial serine/threonine protein kinase PINK1. PINK1 contains an N-terminal mitochondrial localization sequence, a transmembrane sequence, a Ser/Thr kinase domain, and a C-terminal regulatory domain (Schubert et al., 2017) (Figure 7A). We engineered the CRISPR-Cas9 system to induce mutations prior to the kinase domain, thereby eliminating the PINK1 kinase activity (Figure 7A). We generated CRISPR mutants in two genetic backgrounds in which 90% of the cells generated had indels near the PAM region, resulting in dramatically reduced levels of PINK1 protein (Figures S5A and S5B). In addition to the KD, we generated two homozygous knockout lines with one base deletion that caused a frameshift and led to an early stop codon (Figures 7A, 7B, and S5A). The PINK1 knockout lines remain pluripotent and have the capacity to initiate differentiation into all three lineages similar to the WT iPSCs (Figures S5C–S5E). To characterize the metabolic profile of PINK1 knockout hiPSCs, we performed a mitostress assay to measure oxygen consumption rate (OCR) to determine the level of mitochondrial respiration using a Seahorse flux analyzer (Hussein et al., 2020; Sperber et al., 2015; Zhou et al., 2012; Miklas et al., 2019). The OCRs in response to FCCP (an uncoupling agent of mitochondrial oxidative phosphorylation) between WT and PINK1 knockout cells were indistinguishable (Figures 7C and 7D). We conclude that PINK1 KO cells are pluripotent and have a characteristic hiPSC metabolism.

We treated both control hiPSCs and PINK1 KD hiPSCs, as well as the two homozygous knockout hiPSCs, with rapamycin or vehicle for 24 h. As before (Figures S6B–S6D), control hiPSCs showed dense, fused mitochondrial networks, with only ∼10% of cells exhibiting reduced mitochondria and CycE, but after 24 h of treatment with 2 μM rapamycin, mitochondrial and CycE levels were reduced significantly (Figures 7E–7H and S6A–S6G). In contrast, PINK1 mutant hiPSCs showed little to no mitochondrial or CycE reduction, regardless of whether they were treated with DMSO or rapamycin, suggesting that PINK1/Parkin-mediated mitophagy is required for OMM CycE degradation (Figures 7E–7H and S6A–S6I).

We show that rapamycin treatment downregulates H4K16Ac levels in WT, but not in PINK1 knockout cells (Figures 7I and 7J), indicating that PINK-dependent mitophagy is required for a transcriptionally silent iPSC quiescent stage. Moreover, using the PH3^+^ mark to assess hiPSC proliferation, we found that rapamycin reduces cell division by up to 50% in control, but not in PINK1 mutant, hiPSCs (Figures 7L, 7M, S7A–S7D, and S7F). We also show that hiPSCs treated with vehicle (DMSO) for 24 h show a portion of cells in G0/G1 (25.5%), a large portion in S (58.4%), and the remainder in G2/M (14.7%) (Figures 7K and S7G). Conversely, after 24 h of rapamycin treatment, hiPSCs show significant G0/G1 arrest (38.7%), consistent with the idea that rapamycin and mTOR inhibition induces a diapause-like state of cellular quiescence in human hiPSCs. However, rapamycin-treated PINK1 mutant hiPSCs show no significant difference compared with vehicle-treated PINK1 mutant hiPSCs (Figures 7K and S7G). The inability of PINK1 mutant hiPSCs to arrest in G0 is consistent with mitophagy reducing CycE and therefore G1-S transition. These data indicate that hiPSCs, like *Drosophila* GSCs, utilize a non-canonical method of regulating the available reservoir of CycE via PINK1/PARKIN-mediated mitophagy. Together, these findings across stem cell types suggest that diverse stem cells may rely on mitochondrial count to regulate available CycE and, consequently, stem cell quiescence.

## Discussion

In this study, we show that the commencement of quiescence in irradiated *Drosophila* GSCs and hiPSCs relies on the quantity of mitochondria (Figure 7N). We show that a failure in mitochondrial degradation abolishes quiescence, allowing a continuous cell cycle, whereas a failure in mitochondrial biogenesis or fusion impairs the exit from quiescence. Our data further suggest that mitochondrial quantity is controlled by the metabolic sensor mTORC1-dependent epigenetic regulation by PRC1/2. Predominantly, our research suggests that mitochondria mechanistically regulate the cell cycle by serving as a harbor for OMM CycE.

We found that the mTOR-responsive transcription factor Mitf and its downstream targets regulating autophagy are required for quiescence. Although autophagy has been shown to be necessary for stem cell quiescence in some contexts (García-Prat et al., 2016; Ho et al., 2017; Wang et al., 2018) and for proliferation in other contexts (Nagy et al., 2018; Salemi et al., 2012), our data unequivocally show that, in female *Drosophila* GSCs, autophagy is required for quiescence. Further, we report that the mitophagy effectors Pink1 and Parkin, as well as the mitofission protein Drp1, are each necessary for quiescence. Conversely, we found that the mitochondrial biogenesis transcription factor PGC1α and mitofusion protein Mfn2 are required to properly exit from quiescence. In conclusion, the mitochondrial mass tightly regulates reversible quiescence. Given the cross talk between mitophagy and mitochondrial dynamics (Lieber et al., 2019; Deng et al., 2008a, 2008b; Rana et al., 2017; Gong et al., 2015), it will be interesting to dissect whether mitophagy and mitochondrial fission are independent during quiescence, or if they always function in concert. Importantly, in human hiPSCs, we found that rapamycin-induced quiescence requires mitophagy and its regulator PINK1. In contrast, mTORC1 inhibition in mouse embryonic fibroblasts shows increased mitofusion (Morita et al., 2017). Hence, we conclude that mTORC-inhibition-dependent mitophagy might be a unique character of stem cells.

We show that mitophagy reduced mitochondrial CycE and increased G0 cell-cycle arrest in both GSCs and hiPSCs. Our finding that CycE decorates the OMM in hiPSCs raises the question of how CycE is trafficked between the OMM and the nucleus. We have identified a distinct mechanism whereby mitophagy destabilizes the critical G1/S cell cycle regulator, CycE, halting the cell cycle. While the CycE canonical regulator Dacapo/p21 can control DNA-damage-induced p53-dependent checkpoint in G0, p21/Dacapo does not appear to play a role in the GSC insult-induced quiescence (Artoni et al., 2017; Yu et al., 2009). We therefore argue that in stem cells, unexpectedly, the mitochondria quantity may be the primary regulator for CycE and act as a checkpoint for entering and exiting quiescence. It will be important to interrogate which stem cell types can utilize this alternative method of G1/S control, and whether this phenomenon can be leveraged for therapeutic purposes. perhaps utilizing AI-based protein design approach (Cao et al., 2022; Anishchenko et al., 2021).

In satellite stem cells, PcG promotes stemness and self-renewal (Margueron and Reinberg, 2011; Caretti et al., 2004; Bracken et al., 2006; Bianchi et al., 2020). In hematopoietic stem and progenitor cells, the PRC1 component Ring1 is required for self-renewal (Eskeland et al., 2010). Here, we show a novel role for the PcG proteins Pc/CBX8 and Sce/Ring1 of PRC1 as regulators of reversible, injury-induced GSC cell-cycle block. In line with our data, the Pc homolog Cbx7 in African killifish has been recently shown to be required for maintaining diapause (Hu et al., 2020). Furthermore, recent findings suggest that cancer-related irradiation and chemotherapy may lead a class of cancer cells (cancer stem cells, CSCs) to enter a protective, reversible diapause-like state (Dhimolea et al., 2021; Rehman et al., 2021; Hussein et al., 2022). Hence, targeting the PRC1/2 complex (Levy et al., 2022) might force cells to exit diapause-like quiescence in the normal, as well as the cancer, state, guiding CSCs to a proliferative stage amenable to conventional chemotherapies. Future work will be needed to examine whether insult-induced quiescence in GSCs requires Jarid2 via a Polycomb repressive element (PRE)-dependent mechanism like traditional *Drosophila* PcG proteins or via a PRE-independent mechanism like the default mammalian method (DeLuca et al., 2020; Herz et al., 2012; Landeira et al., 2010).

The cornerstone of stem cell quiescence in this study is PINK1/PARKIN-mediated mitophagy, which acts downstream of mTOR-dependent PRC2 complex activity (Figure 7N). It is plausible that the CycE pool associated with mitochondria gets directly ubiquitinated by Parkin, leading to an overall decrease in Cdk2/CycE activity essential for G1/S transition, resulting in a reversible G0 cell-cycle halt (Figure 7N). Future research will reveal whether manipulating mTORC1 signaling and mitochondria quantity could restore regenerative capacity to non-regenerative adult tissues like the heart (Miklas et al., 2022). Furthermore, it will be important to dissect in detail if and how mitochondrial dynamics play a role in GSC age-related senescence, as mitochondria have been shown to play a critical role in senescence in many cell types (Rana et al., 2017; Davalli et al., 2016; Scheckhuber et al., 2007; Chaudhari and Kipreos, 2017; Byrne et al., 2019; Pauklin and Vallier, 2014; Tai et al., 2017). It will be important to investigate if similar epigenetic regulation of mitochondrial dynamics controls insult-induced quiescence in other stem cell types and whether senescence in aging stem cells can be reversed by altering mitochondrial dynamics.

## Experimental procedures

### Resource availability

#### Corresponding author

Further information and requests for resources and reagents should be directed to and will be fulfilled by the corresponding author, Hannele Ruohola-Baker (hannele@uw.edu).

#### Materials availability

All unique/stable reagents generated in this study are available from the corresponding author without restriction.

#### Data and code availability

Description of sample size and raw data are provided in the supplemental information. For further information, please contact the corresponding author.

### Fly stocks and culture conditions

Flies were cultured at 25°C on cornmeal-yeast-agar medium supplemented with wet yeast (Artoni et al., 2017; Xing et al., 2015). Information regarding fly stocks is given in the supplemental experimental procedures.

### hiPSC culture conditions

The hiPSCs were cultured on Matrigel growth factor-reduced basement membrane matrix (Corning) in mTeSR medium (STEMCELL Technologies). The cells were treated with rapamycin (200 nM–2 μM, Fisher Scientific) or DMSO for indicated periods and thereafter tested for reversion in the absence of rapamycin. To analyze the proteasome function, hiPSCs were treated with 100 nM MG132 (proteasomal inhibitor, Thermo Scientific; cat. no. 508339) for 24 h. Information regarding cell lines is given in the supplemental experimental procedures.

### Ionizing radiation treatment in *Drosophila* and hiPSCs

Prior to gamma-irradiation exposure, 2- to 4-day-old flies (5–6 M:15–18 F) were kept on cornmeal-yeast-agar medium augmented with wet yeast for 48 h at 25°C. On the day of irradiation, two-thirds of the females and all males were transferred to empty plastic vials and treated with 50 Gy of gamma-irradiation. More details are provided in the supplemental experimental procedures. For irradiation in hiPSCs, we plated 5 × 10^4^ hiPSCs in Matrigel-coated coverslip in 35 mm culture dishes. After 48 h, the dishes were treated with 0.52 Gy of gamma-irradiation. One day post-insult, the cells were fixed in paraformaldehyde and stained.

### OCR measurement using Seahorse cellular flux assay

The Seahorse XF96 extracellular flux analyzer was used to assess mitochondrial function as described (Sperber et al., 2015; Zhou et al., 2012; Miklas et al., 2019). Details are given in the supplemental experimental procedures.

### *Drosophila* and hiPSC immunofluorescence analysis

Fly samples and iPSCs were fixed and stained with different antibodies. Details regrading the immunofluorescence is given in the supplemental experimental procedures.

### hiPSC gene knockout and knockdown

Guide RNAs (gRNAs) targeting exon 2 of PINK1 (Table S2) were designed using CRISPOR.org (Concordet and Haeussler, 2018) and inserted into a lentiCRISPR v.2 plasmid containing two expression cassettes, hSpCas9 and the chimeric guide RNA (Shalem et al., 2014; Sanjana et al., 2014) (a gift from Feng Zhang; Addgene plasmid 52961), as done before (Arena et al., 2021). The vector was digested using *BsmB*I, and a pair of annealed oligos were cloned into the single guide RNA scaffold. Sequences for the gRNAs and primers used for sequencing can be found in Tables S2 and S3, respectively.

For CycE KD, a million hiPSCs cells were seeded on a Matrigel-coated dish with mTeSR medium with a mixture of CycE siRNA (Dharmacon ON-target Plus SMARTpool siRNA) (Table S4) and RNAi Max (Invitrogen; 13778-075) prepared in Opti-MEM (Gibco; 51985).

### hiPSC transduction and selection

hiPSCs were transduced with lentiCRISPR v.2/gRNA lentiviral particles targeting PINK1 with 4 μg/mL polybrene (Sigma Aldrich). Details are provided in the supplemental experimental procedures.

### Trilineage differentiation

We differentiated the WT and PINK1 KO hiPSC lines using a STEMdiff Trilineage Differentiation Kit (STEMCELL Technologies; 05231, 05232, 05233). We performed qPCR for lineage-specific markers using a Human Pluripotent Stem Cell Trilineage Differentiation qPCR array kit (STEMCELL Technologies; 07515) and analyzed their differentiation efficiency using the software www.stemcell.com/qPCRanalysis.

### Data and resources availability

Description of sample size, raw data, and additional information on experimental procedures are provided in the supplemental information. For further information, please contact the corresponding author.

## Author contributions

Conceptualization, T.H.T., A.M.H., R.K., J.R.I., J.M., and H.R-B.; data curation, formal analysis, validation, and investigation, T.H.T., A.M.H., R.K., J.R.I., T.C.C., B.N.N., S. Liu, D.B., S.H., S. Lyons, B.G., J.D., N.B., S.J., D.D.C., and J.M.; methodology, T.H.T., A.M.H., R.K., J.R.I., and T.C.C.; visualization, T.H.T., A.M.H., R.K., J.R.I., S. Lyons, S.H., J.M., N.B., and S.J.; writing – original draft, T.H.T., J.R.I., and H.RB., writing – review & editing, T.H.T., A.M.H., R.K., J.R.I., S. Liu, J.M., and H.R-B.; resources, J.M. and D.D.C.; funding acquisition, H.R.B.; project administration, H.R.B.

## Supplemental information

Document S1. Experimental procedures, Figures S1–S7, and Tables S2–S4

Table S1. Excel file compiling all IR paradigm source data relevant to the experiments Data are organized into sheets that they represent in the main and supplemental figures, followed by genotype, time points and replicate number.

Document S2. Article plus supplemental information

## Footnotes

Supplemental information can be found online at https://doi.org/10.1016/j.stemcr.2022.11.004.
