# Supplementary material for: Stress-induced reversible cell-cycle arrest requires PRC2/PRC1-mediated control of mitophagy in Drosophila germline stem cells and human iPSCs
Source: Stem Cell Reports. 2022 Dec 8;18(1):269–88. doi: 10.1016/j.stemcr.2022.11.004 (PMC9860083; doi:10.1016/j.stemcr.2022.11.004)
Supplement: Document S1. Experimental procedures, Figures S1–S7, and Tables S2–S4 [file mmc1.pdf]

Stem Cell Reports, Volume *18*

Supplemental Information

Stress-induced reversible cell-cycle arrest requires PRC2/PRC1-mediated control of mitophagy in *Drosophila* germline stem cells and human iPSCs

Tommy H. Taslim, Abdiasis M. Hussein, Riya Keshri, Julien R. Ishibashi, Tung C. Chan, Bich N. Nguyen, Shuozhi Liu, Daniel Brewer, Stuart Harper, Scott Lyons, Ben Garver, Jimmy Dang, Nanditaa Balachandar, Samriddhi Jhajharia, Debra del Castillo, Julie Mathieu, and Hannele Ruohola-Baker

SUPPLEMENTAL EXPERIMENTAL PROCEDURES:

Fly stocks and culture conditions:

The following stocks were obtained from the Bloomington *Drosophila* Stock Center at Indiana University: w^{1118} (RRID:BDSC_3605), UAS-Dcr2, w^{1118}; nos-Gal4 (RRID:BDSC_25751), w*;;nos-Gal4 (RRID:BDSC_25394), w*; UAS-GFP (RRID:BDSC_6874), UAS-Tsc1$^{RNAi\ \#1}$ (RRID:BDSC_35144), UAS-Tsc1$^{RNAi\#2}$ (RRID:BDSC_54034), (RRID:BDSC_33914), UAS-Cnc^{RNAi} (RRID:BDSC_40854), UAS-Raptor$^{RNAi\#1}$ (RRID:BDSC_41912), UAS-Raptor$^{RNAi\#2}$ (RRID:BDSC_34814),UAS-Nprl3^{RNAi} (RRID:BDSC_55384), UAS-Mitf$^{RNAi\#1}$ (RRID:BDSC_44561), UAS-Mitf$^{RNAi\#2}$ (RRID:BDSC_43998), UAS-Nup44^{RNAi} (RRID:BDSC_39357), UAS-Mio^{RNAi} (RRID:BDSC_57745), UAS-Rictor^{RNAi} (RRID:BDSC_36699), UAS-Rictor^{RNAi} (RRID:BDSC_36584), UAS-Nprl2^{RNAi} RRID:BDSC_57538), UASp-mCherry-Atg8a (RRID:BDSC_37750), UASp-GFP-mCherry-Atg8a (RRID:BDSC_37749), UAS-Atg3^{RNAi} RRID:BDSC_34359), UAS-Atg7^{RNAi} (RRID:BDSC_34369), UAS-Atg5^{RNAi} (RRID:BDSC_34899), UAS-Atg12^{RNAi} (RRID:BDSC_34675), UAS-Atg18a^{RNAi} (RRID:BDSC_34714), UAS-Atg14^{RNAi} (RRID:BDSC_40858), UAS-Atg13^{RNAi} (RRID:BDSC_40861), UAS-Atg1^{RNAi} (RRID:BDSC_44034), UAS-Atg16$^{RNAi\#1}$ (RRID:BDSC_58244), UAS-Atg1 (OE-1) (RRID:BDSC_51654), UAS-Atg1B (OE-2) (RRID:BDSC_51655), UAS-ref(2)P^{RNAi} (RRID:BDSC_36111), UAS-RUBCN^{RNAi} (RRID:BDSC_43276), UAS-Atg16$^{RNAi\#2}$ (RRID:BDSC_34358), UAS-Pink1^{RNAi} (RRID:BDSC_38262), UAS-Park^{RNAi} (RRID:BDSC_37509), UAS-Drp1^{RNAi} (RRID:BDSC_51483), UAS-Marf^{RNAi} (RRID:BDSC_55189), UAS-Srl^{RNAi} (RRID:BDSC_33914), UAS-Su(z)12^{RNAi} (RRID:BDSC_33402), UAS-Kdm2^{RNAi} (RRID:BDSC_33699), UAS-trx^{RNAi} (RRID:BDSC_33703), UAS-Pc^{RNAi} (RRID:BDSC_33964), UAS-Gcn5^{RNAi} (RRID:BDSC_33981), UAS-rhi^{RNAi} (RRID:BDSC_34071), UAS-Utx^{RNAi} (RRID:BDSC_34076), UAS-HDAC1^{RNAi} (RRID:BDSC_34846), UAS-Set1^{RNAi} (RRID:BDSC_40931), UAS-mei-41^{RNAi} (RRID:BDSC_41934), UAS-Mt2^{RNAi} (RRID:BDSC_42906), UAS-gpp^{RNAi} (RRID:BDSC_42919), UAS-JIL-1^{RNAi} (RRID:BDSC_57293), UAS-mof^{RNAi} (RRID:BDSC_58281), UAS-Jarid2^{RNAi} (RRID:BDSC_40855), UAS-Jing^{RNAi} (RRID:BDSC_35750), UAS-E(z)^{RNAi} (RRID:BDSC_36068), UAS-Sce^{RNAi} (RRID:BDSC_67924), w^{1118}; UAS-GFP-E2F1$_{1-230}$ UAS-mRFP1-CycB$_{1-266}$ (RRID:BDSC_55110) and w^{1118};UAS-GFP-E2F1$_{1-230}$ UAS-mRFP-NLS-CycB$_{1-266}$ (RRID:BDSC_55111).

hiPSC culture conditions

The human induced pluripotent stem cells (hiPSC) line WTC-11 (Coriell Institute, GM25256), was derived in the Conklin laboratory (Kreitzer et al., 2013), the EGFP-TOM20 WTC hiPSC line was generated by the Allen Institute for Cell Science (Coriell Institute, AICS-0011) and expanded in the Ellison Stem Cell Core at the University of Washington (UW-AICS-0011).

Ionizing radiation treatment in *Drosophila* and hiPSCs:

A Cs-137 Mark I Irradiator was used to administer the proper irradiation dosage, according to instructed dosage chart. The remaining 1/3 of the females were not irradiated and were dissected within 1h of the others receiving irradiation treatment. After irradiation, the flies (with fresh, unirradiated males) were flipped onto a new vial of Standard Diet augmented with wet yeast at 25°C. ½ of the remaining females were dissected at 1-day post-insult (1 dpi). The remaining females were dissected at 2-days post-insult (2 dpi) (Figure 1A).

Drosophila and hiPSCs immunofluroscence analysis:

Germarium in the Drosophila ovary (Figure-1B) consist of Terminal filament (TF) cells (magenta, cuboidal) comprise the anterior tip of the ovary and connect to the Cap cells (CpCs) (magenta, planar);

Germline stem cells (GSCs) contain anterior spectrosomes (red, circular) which elongate (red, linear) during division; undifferentiated cystoblasts (CBs) contain spectrosomes (red, circular) or early fusomes (red, linear) but have no junctions to the CpCs; germ cell cysts (GCCs) are identified by presence of a branched fusome (red, branched); all GSCs and progeny contain nuclei (blue, circular); follicle stem cells (FSCs) (cerulean) can be identified by their distinct triangular morphology; mature GCCs are encapsulated by the prefollicle cells (PFCs) (lime green, planar) and proceed through oogenesis. Upon irradiation GSCs enter quiescence, shown by the absence of elongated spectrosomes and GSCs progenitor cells die. Later, GSCs exit quiescence and resume division, shown by the return of elongated spectrosomes and recovers the lost progenies.

Fly samples were dissected in cold PBS and fixed in 4% paraformaldehyde for 15 min at room temperature within 30 min of dissection. Samples were then rinsed in PBT (PBS containing 0.2% Triton X-100), blocked in PBTB (PBT containing 0.2% BSA, 5% normal goat serum) for at least one hour at room temperature. Samples were stored for up to 72 hours at 4℃ in PBTB. Samples were incubated with primary antibodies for 24 hours at 4℃. After PBT washes, fluorophore-conjugated secondary antibodies were utilized for 1.5–2 hours at room temperature in the dark. Samples were then washed with DAPI, diluted with PBT to 2 μg/ml, for 15 minutes to visualize nuclei, followed by two PBT washes. The samples were mounted in mounting medium (21 mL of Glycerol, 2.4ml of 10x PBS and 0.468g of N-Propyl Gallate) and analyzed on Leica SPE5 confocal and Leica SP8 confocal laser-scanning microscope. Primary and secondary antibodies are listed in the supplemental experimental procedures.

For hiPSCs, cells were washed with PBS (twice, 5 min each) and fixed in 4% paraformaldehyde in PBS for 15 min and blocked for 1 h in 3% BSA+0.1% Triton X-100. The cells were then incubated in primary antibody overnight at 4 °C, washed with PBS (thrice, 5 min each), incubated with the secondary antibody in 3% BSA+0.1% TritonX100 for 2 h at room temperature, washed in PBS (thrice,10 min each) and stained with 2 μg/ml DAPI diluted with 1X PBS for 15 min. Mounting media was composed of 21 ml of Glycerol, 2.4ml of 10X PBS and 0.468g of N-Propyl Gallate. Analysis was done on Leica SPE5 Confocal microscope using a ×63 objective and Leica Software. Antibodies used are listed in supplemental experimental procedures.

**DROSOPHILA ANTIBODIES**	**DILUTION**	**MANUFACTURER**
mouse anti-adducin	1:20	RRID:AB_528070
mouse anti-Lamin C	1:20	RRID:AB_528339
mouse anti-ATPsynβ	1:500	RRID:AB_301438
mouse anti-Fasciclin III	1:50	RRID:AB_528238
rat anti-cyclin E	1:200	A gift from Helena Richardson
rabbit anti-GFP antibody	1:500	RRID:AB_221569
mouse anti-mCherry antibody	1:500	RRID:AB_11133266
rabbit anti-mCherry antibody	1:250	RRID:AB_2650480
rat anti-Vasa antibody	1:20	RRID:AB_760351
rabbit anti-Dcp-1	1:100	RRID:AB_2721060
rabbit anti-DsRed	1:500	RRID:AB_10013483
chicken anti-GFP	1:2000	RRID:AB_300798
mouse anti-1B1	1:20	RRID:AB_528070
rabbit anti-H3K27me3	1:250	RRID:AB_ 2561020
anti-rabbit Alexa 488	1:250	RRID:AB_221544
anti-rabbit Alexa 568	1:250	RRID:AB_143157
anti-mouse Alexa 488	1:250	RRID:AB_2534069
anti-mouse Alexa 568	1:250	RRID:AB_2535773
anti-rat Alexa 568	1:250	RRID:AB_2534121
anti-chicken Alexa 488	1:250	RRID:AB_2762843
anti-mouse Alexa 647	1:250	RRID:AB_2535805
anti-rabbit Alexa 647	1:250	RRID:AB_2535812

hiPSC ANTIBODIES	DILUTION	MANUFACTURER
Anti-Oct-4	1:100	Santa Cruz
Anti-ATP synthase β	1:500	Abcam, Ab14730
Anti-cyclin E human	1:100	Santa Cruz, sc-481
Anti-TFE3	1:400	Sigma Prestige HPA023881
anti-HADHA	1:250	Abcam, ab54477
anti-Phospho-Histone 3 (Ser10)	1:250	Millipore Sigma, 06-570
GFP-Booster Alexa Fluor 488	1:300	ChromoTek, gb2AF488
Alexa Fluor 568 Phalloidin	1:300	Invitrogen, Cat: A12380
Alexa 488 or 568 or 647-conjugated secondary antibody	1:250	Molecular Probes

Categorization of reduced mitochondria

hiPSCs are considered containing reduced mitochondria when (1) few interconnected network of mitochondria tubules, but small independent mitochondria, is observed, (2) small mitochondria fragments scattered around the periphery of the nuclei, and (3) have significantly lower mitochondria: nuclei area compared to hiPSCs with undegraded mitochondria. hiPSCs are considered containing normal mitochondria when (1) extensive interconnected network of mitochondria tubules is observed, (2) network of mitochondria tubules form elongated polarized clusters extended from the nuclei, and (3) have significantly higher mitochondria: nuclei area compared to hiPSCs with the fragmented mitochondria.
GSCs are considered containing reduced mitochondria when (1) few interconnected network of mitochondria tubules, but small independent mitochondria, is observed, (2) small mitochondria fragments scattered around the periphery of the nuclei, and (3) have significantly lower mitochondria: nuclei area compared to GSCs with non- reduced mitochondria. GSCs are considered containing non-reduced mitochondria when (1) extensive interconnected network of mitochondria tubules is observed, (2) network of mitochondria tubules form elongated polarized clusters extended from the nuclei towards the anterior end of the germarium, and (3) have significantly higher mitochondria: nuclei area compared to GSCs with the fragmented mitochondria.

Mitochondria Area Analysis

Fixed number of confocal image slices showing only the mitochondria in the middle section of the cell were compressed using ImageJ Z-project feature. Area of interest (containing only the mitochondria of one cell) was selected using crop function. With an additional ImageJ plugin, Versatile Wand Tool, and using a fixed value for Value of Tolerance for all quantification, area of mitochondria was selected and measured.

H3K27me3 fluorescence intensity quantification

The mean pixel intensity of the H3K27me3 immunofluorescence staining was measured using ImageJ analysis software. Germline Stem Cells were identified by characteristic Adducin staining adjacent to cap cells in each germarium. Regions of Interest (ROI) were drawn around non-dividing GSC nuclei and mean pixel intensity in each ROI was measured inside each ROI. Mean pixel intensity was compared across control, 1 dpi and 2 dpi GSCs.

Image Deconvolution and 3D Imaging

Images taken from SPE5 confocal laser-scanning microscope were deconvoluted using Leica LIGHTNING software. 3D hiPSCs images were taken using GE DeltaVision OMX SR super-resolution microscope. Deconvoluted confocal images and OMX images were analyzed with Imaris (Bitplane)

program to construct respective 3D models in Maximum Intensity Projection (MIP) mode for *Drosophila* GSCs, and Normal Shading for hiPSCs.

Viral production

HEK 293FT cells were plated one day before transfection. On the day of transfection, the lentiCRISPRv2 plasmid containing the gRNA was combined with packaging vectors psPAX2 (a gift from Didier Trono, Addgene plasmid # 12260) and pMD2.G (a gift from Didier Trono Addgene plasmid # 12259) in the presence of 1 µg/µl of polyethylenimine (PEI) per 1 µg of DNA. Medium was changed 24 hours later, and the lentiviruses were harvested 48 and 72 h after transfection. Viral particles were concentrated using PEG-it (System Biosciences, Inc).

hiPSCs transduction and selection:

After hiPSCs were transduced with lentiCRISPR-v2/gRNA lentiviral particles, the media was changed the next day. 48h after infection, cells were selected with puromycin (1µg/ml) for two days and genomic DNA was extracted using DNAzol reagent (Invitrogen) and quantified using Nanodrop ND-1000. Genomic regions flanking the CRISPR target sites were PCR amplified with designed primers (Supplemental Table 3) using GoTaq DNA polymerase (Promega) and sent for Sanger sequencing to determine the insertion and deletion errors generated by CRISPR-Cas9 system in exon 2 PINK1 gene. The editing efficiency and KO score were determined using the Synthego ICE analysis tool. The mutant lines were further analyzed.

Western Blot Analysis

$5x10^4$ hiPSCs were plated on 35mm Matrigel coated plates and lysed in lysis buffer containing 20 mM Tris-HCl pH 7.5, 150 mM NaCl, 15% glycerol, 1% Triton x-100, 1 M ß-glycerolphosphate, 0.5 M NaF, 0.1 M sodium pyrophosphate, Sodium orthovanadate, PMSF, and 2% sodium dodecyl sulfate (SDS). Twenty-five units of Benzonase® Nuclease (EMD Chemicals, Gibbstown, NJ) was added to the lysis buffer right before use. The protein samples were combined with the 4× Laemmli sample buffer, heated (95°C, 5 min), run on SDS-PAGE (protean TGX pre-casted 4–20% gradient gel; Bio-rad) and transferred to nitrocellulose membrane (Bio-Rad) by semi-dry transfer (Bio-Rad). Membranes were blocked for 1 h with 5% milk or BSA and incubated in primary antibodies overnight at 4°C.The membranes were then incubated with secondary antibodies (1:10000, goat anti-rabbit or goat anti-mouse IgG HRP conjugate (Bio-Rad) for 1 hour and detected using the Immobilon-luminol reagent assay (EMP Millipore).

PRIMARY ANTIBODY	DILUTION	CATALOGUE NO.	MANUFACTURER
Beta-Actin	1:10000	4970	Cell Signalling
p-mTOR(Ser 2448)	1:1000	5536	Cell Signalling
mTOR	1:1000	2972	Cell SIgnalling
pS6	1:1000	2215	Cell Signalling
S6	1:1000	2117	Cell Signalling
Histone 4 (lys16) Acetylation (H4K16Ac)	1:1000	07-329	Millipore Sigma
PINK1 D8G3	1:1000	6946	Cell signalling

OCR Measurement Using Seahorse Cellular Flux Assay

The plates were pre-treated with 1:30 diluted Matrigel reduced growth factor (Corning). hiPSCs (WT and PINK1 KO) were seeded onto 96-well Seahorse plates at $3X10^4$ cells/well. Cells were cultured in DMEM with glucose overnight. Culture media were exchanged for base media (unbuffered DMEM

(Sigma D5030) supplemented with sodium pyruvate (Gibco, 1 mM) and with 25 mM glucose (for Mitostress assay) 1 hour prior to the assay. Substrates and selective inhibitors were injected during the measurements to achieve final concentrations of 4-(tri-fluoromethoxy) phenylhydrazone (FCCP, 1 μM), oligomycin (2.5 μM), antimycin (2.5 μM) and rotenone (2.5 μM). The OCR values were normalized to the number of cells present in each well, quantified by the hoechst staining (HO33342; Sigma-Aldrich) as measured using fluorescence at 355 nm excitation and 460 nm emission. Maximal OCR is defined as the change in OCR in response to FCCP compared to OCR after the addition of oligomycin.

Cell cycle Analysis via flow cytometry

hiPSCs, either WT, or PINK1 mutants, were seeded in 35 mm dishes at a density of $1.0x10^5$ cells. After 48 h of culture, cells were treated with 2 μM rapamycin or DMSO and 24 hours later, cells were harvested, and cell suspensions were pelleted and washed twice with PBS at 300 xg for 5 minutes. Cells were resuspended in 2% FBS in PBS and cells were fixed with 5 mL cold 70% ethanol for at least 24 hours (-20°C) and then centrifuged 10 minutes at 500 x g at 4°C. Cells were washed twice with 3 ml PBS at 400 xg for 5 minutes at 4°C and 500 ul of PI staining buffer was added (1X PBS, 50 μg/ml PI (Calbiochem), 2μg/ml Rnase A (Sigma) and 0.1% Igepal (Sigma)) for 3 hours at 4°C. Cell cycle analysis was performed using the FACS Canto II flow-cytometer (BD Biosciences). Data analysis was performed using the FlowJo software (Tree Star, Ashland, OR, USA).

Statistical analysis
All data are presented as the mean of $n \geq 3$ experiments with the standard error of the mean (SEM) indicated by error bars, unless otherwise indicated. Statistical significance was determined using chi-squared test (between timepoints) or Student's t-test (between conditions). Only p values of 0.05 or lower were considered statistically significant ($p > 0.05$ [ns, not significant], $p \leq 0.05$ [*], $p \leq 0.01$ [**], $p \leq 0.001$ [***], $p \leq 0.0001$ [****]). For analysis of quiescence, only fold changes of ≥1.6 were evaluated for significance. Data were compiled and analyzed with Excel for Mac (2020; Microsoft, Seattle, WA, USA).

SUPPLEMENTARY FIGURE LEGENDS:

SUPPLEMENTAL FIGURE 1: Role of mTORC1 in regulating insult-induced quiescence in female GSCs
(A) Representative confocal microscopy images of Rictor or Nrf2 RNAi KD from unirradiated, 1dpi, and 2dpi germaria stained with 1B1 (red, spectrosomes/fusomes), LamC (red, Cpc and TF) and DAPI (blue, nuclei). Dotted circle represents GSC (Scale bar 5μm). **(B)** Representative confocal microscopy images of control, Jarid2 and Pink1 RNAi KD from unirradiated, 1dpi, and 2dpi germaria stained with 1B1 (green, spectrosomes/fusomes), LamC (green, Cpc and TF), PH3 (red, proliferating cell nuclei) and DAPI (blue, nuclei). Dotted circle represents GSC (Scale bar 5μm). (**C-D**) Quantification of PH3 positive GSCs of control (**C**) Jarid2 RNAi KD (**D**) and Pink1 RNAi KD (**E**) from the images in S1**B**. (**F**) Quantification of GSCs with reduced mitochondria. Injury-induced mitochondrial fragmentation is observed in the G_0 stage of GSC cell cycle (n=203 for each).

SUPPLEMENTAL FIGURE 2: Autophagy-defective germline stem cells display impaired quiescence
(A) Schematic diagram of how UASp-EGFP-mCherry-Atg8a tandem fusion can be used to study autophagic flux. **(B)** Representative confocal microscropy images of nos>mCherry-Atg8a from unirradiated, 1 dpi, and 2 dpi germaria stained with mCherry (red, autophagosome/autolysosome), VASA (cyan) and DAPI (blue, nuclei). Dotted circle represents GSC (Scale bar 5 μm). **(C)** Bar graph depicting the proportion of punctae+ GSCs/total GSCs in nos>GFP-mCherry-Atg8a. **(D)** Immunofluorescence images of GSCs with respective core autophagy component RNAi KD. Stained with 1B1 (red, spectrosomes/fusomes), LamC (red, Cpc and TF) and DAPI (blue, nuclei).

Dotted circle represents GSC (Scale bar 5 μm). **(E)** Percentage of dividing GSC of control and Atg1 OE from unirradiated, 1 dpi, and 2 dpi germaria.

SUPPLEMENTAL FIGURE 3: Epigenetic proteins regulate GSC quiescence

(A-I) Representative confocal microscopy images of epigenetic genes RNAi KD from unirradiated, 1dpi, and 2dpi germaria stained with 1B1 (red, spectrosomes/fusomes), LamC (red, Cpc and TF) and DAPI (blue, nuclei). Dotted circle represents GSC (Scale bar 5μm). **(J)** A graph quantification of GSC division. Control GSCs divide at baseline level when unirradiated (28%), which sharply decreases at 1dpi (11%), and recovers to near baseline at 2dpi (23%). Epigenetic regulators, JIL1, Mof/KAT8, Mei-41/ ATR, Set1, Gpp/DOT1L are required for entry into quiescence. (n=3 for control. n=2 for JIL-1, mof, and mei-41/ATR). Following is the total number of GSCs quantified (n) for each condition in the order from unirradiated, 1dpi and to 2dpi time point: Control: 261, 119,90. JIL-1 RNAi: 135,128,103. Mof RNAi: 140,151,140. Mei-41 RNAi: 184,196,105. **(K)** Representative confocal microscopy images of GSCs with respective RNAi KD from unirradiated, 1dpi, and 2dpi germaria, which were used for fragmentation quantification on Fig. **5F**. stained with DAPI (blue, nuclei) and ATPsynβ (green, mitochondria) throughout the experimental timepoints (Scale bar 5μm). **(L-M)** Representative 3D reconstructed confocal microscopy images of (**L**) Tsc1-KD and (**M**) Atg3-KD GSC lines from unirradiated, 1dpi, and 2dpi time points stained with DAPI (blue, nuclei) and ATPsynβ (green, mitochondria). ('A' denotes anterior side, and 'P' denotes the posterior side of the GSC). Arrow points to the area of interest, where mitochondria are typically clustered (Scale bar 1μm). (**N**) Bar graph depicting the proportion of punctae+ GSCs/total GSCs in nos>-mCherry-Atg8a Pink1 RNAi and Jarid2 RNAi (left panel) and their respective representative confocal microscopy images (right panel). (Scale bar 5μm) (**O**) A graph quantification of the percentage of dividing GSCs of E(z) RNAi KD unirradiated, 1dpi, and 2dpi germaria. Following is the total number of GSCs quantified (n) for each condition in the order from unirradiated, 1dpi and to 2dpi time point: 165,27,38. **(P)** Representative confocal microscopy images of control GSCs from unirradiated, 1dpi, and 2dpi germaria stained with Dapi, cyclin E and ATPsynβ. **(Q)** Left panel: Top - GSC with reduced mitochondria; Bottom - GSC with non-reduced mitochondria. Right panel: Quantification of GSC reduced mitochondria area. (n=2 for both). Following is the the total number of GSCs quantified (n) for each condition. n= 51 for GSCs with reduced mitochondria, n=87 for GSCs without reduced mitochondria. **(R)** Summary model of Drosophila GSC showing an initial pules of FOXO activating Tsc1 which inhibits mTORC1, which in turn derepresses fission, mitophagy, and PRC1. Ultimately, mitochondrial fragmentation and degradation seem to constitute a "checkpoint" of sorts before entering quiescence. Then, in order to exit quiescence, H3K27me3 demethylase needs to reactivate specific genes, likely those involved in mitochondrial fusion and biogenesis.

SUPPLEMENTAL FIGURE 4: Mitochondrial degradation is required for iPSCs to enter quiescence

(A-C) Quantification of (**A**) mitochondrial reduction (**B**) and nuclear TFE3 in unirradiated and irradiated wildtype hiPSCs and **(C)** their respective representative confocal microscopy images. Scale bar- $50\mu m$. **(D)** WTCT cells treated with 2μM rapamycin for 3 hours, 7 hours or 24 hours, stained with ATP synβ (mitochondria, red), Cyclin E (green) and DAPI (blue) (Scale bar 5μm). **(E-F)** Western blot analysis for pulse chase experiment of wildtype hiPSC treated with either vehicle control (DMSO) or rapamycin (2 μM) for 7 hours **(E)** or 24 hours **(F)** followed by reversion (normal hiPSCs growth media) for another 96 hours. pmTOR, pS6 and H4K16Ac level is seen to be low by 7 or 24 hours post 2μM rapamycin treatment and their levels increase by 96 hours post reversion. **(G)** representative confocal images of hiPSCs treated with DMSO and Rapamycin(2μM) for 24 hours; stained for DAPI (blue) and TFE3 (red) (Scale bar 10 μm); a graph showing rapamycin treatment promotes significant nuclear localisation of TFE3 in hiPSCs. **(H)** Raw images of Figure 6C representative 3D-reconstructed OMX super resolution microscopy images of WTC cells treated with vehicle control (DMSO) or 2μM rapamycin for 24hrs. Stained with ATPsynβ (green), cyclin E (red) and DAPI (blue) and the white dotted line is used to quantify intensity profile in (D) (Scale bar 1 μm). **(I)** Representative high magnification 3D reconstructed OMX super resolution microscopy images of wildtype cells treated with DMSO vehicle or 2μM rapamycin for 24 hours, stained with ATPsynβ (mitochondria, green), Cyclin E (red) and DAPI (blue) (Scale bar 0.1μm). **(J)** Representative western blot analysis of Wildtype

treated with DMSO, 2μM rapamycin, EPZ-6438 and rapamycin+ EPZ-6438 for 24hrs and blotted with pmOTR and Beta-actin as a loading control. **(K)** Quantification of H3K27me3 intensity fold change in DMSO, 2μM rapamycin, EPZ-6438 and rapamycin + EPZ-6438 (images from Fig 6L were used for this quantification) for 24hrs, n=3. **(L)** Relative fluorescence of CycE and ATP synβ of Wildtype cells treated with vehicle (DMSO) or rapamycin for 7 hours (quantified from Fig. 6B). **(M)** Representative confocal microscopy images of CycE staining in wildtype hiPSC shows accumulation of CycE in DMSO, which is reduced upon rapamycin treatment and not rapamycin with MG132 (100nM, proteasomeme inhibitor). Scale bar =10μm.; a graph showing cyclin E intensity over DAPI area in DMSO, rapamycin and rapa with MG132 treated condition.Fig. A-F,H,L: Wildtype/control= WTC-Tom20. Fig. G: Wildtype= WTC**.**

Supplemental Figure 5: Mutant pool in PINK1 CRISPR/Cas9 knockout cells
(**A**) Pink1 structure with guide RNA location indicated and DNA sequencing chromatogram comparing wildtype (WT) PINK1 to a PINK1 knockout clone, showing the deletion in guanine, which creates an early stop codon, and another mutant PINK1 which shows a mixed pool of mutants and a loss of the wildtype sequence. **(B)** Western Blot showing lysates from Control wildtype WTC, PINK1 mutant WTC and PINK1 mutant WTC Tom-20, showing PINK1 protein knocked down in both mutant pools when compared to control. **(C)** Representative confocal microscopy images of wildtype and PINK1 knockout cells stained with DAPI and the pluripotent marker, Oct4. **(D-E)** Box plot data of 384-well qPCR array using wildtype and PINK1 knockout cells differentiated into all three lineages. Both wildtype (**D**) and PINK1 knockout cells (**E**) show their differentiation capacity into all three germ layers (endoderm, mesoderm and ectoderm). Fig. A-D: wildtype =WTC-Tom20.

SUPPLEMENTAL FIGURE 6: iPSCs require mitophagy to regulate Cyclin E
(**A-B, D, G-H**) Representative confocal microscopy images of control and/or Pink1-KD iPSCs, treated with either DMSO or 2μM rapamycin for 24 hours. (**A-B**) Deconvoluted images taken from SP8 confocal microscope. (**A**) Wildtype and Pink1-KD WTC cells stained with ATPsynβ (mitochondria, green), Cyclin E (red) and DAPI (blue) (Scale bar 10μm). (**B**) High magnification of Wildtype and Pink1-KD WTC cells stained with ATPsynβ (mitochondria, green), Cyclin E (red) and DAPI (blue) (Scale bar 5μm). (**C**) Quantification of cells without a dense cluster of mitochondria in wildtype vs Pink1-KD WTC treated with control DMSO or 2μM rapamycin for 24 hours, based on just the mitochondria (left panel) or mitochondria that co-localizes with cyclin E (right panel). Each group shows wildtype WTC increase in cells without dense cluster of mitochondria when treated with rapamycin vs DMSO, whereas Pink1-KD WTC cells maintains their dense cluster of mitochondria when treated with rapamycin. Black bar shows quantification for DMSO treated cells and grey bar for 2μM rapamycin treated cells. Data is from duplicate experiments. Following are the total number of cells quantified (n) for WT and Pink1-KO WTCT for DMSO and Rapamycin treated cells respectively: WT (without Cyclin E stain):1090,1022. WT (with cyclin E stain): 566, 533. Pink1-KO WTCT (without Cyclin E stain):725,754. WT (with cyclin E stain): 605,741. (**D**) Low magnification images (left panel) of control and Pink1-KD WTCT cells stained with ATPsynβ (mitochondria, green), Cyclin E (red) and DAPI (blue) (Scale bar 15μm). High magnification images (right manel) of wildtype WTCT or Pink1-KD mutant WTCT treated with either vehicle control (DMSO) or rapamycin (2μM) stained with DAPI (blue), CycE (red) and ATPsynβ (mitochondria, green) (Scale bar 5μm). (**E**) Representative 3D reconstructed OMX super resolution microscopy images of wildtype WTC or Pink1 mutant WTC stained with DAPI (blue), CycE (red), and ATPsynβ (mitochondria, green) (Scale bar 0.2μm). (**F**) Quantification of mitochondrial degradation in WTCT vs Pink1-KD mutant treated with either vehicle control (DMSO) or rapamycin (2μM), based on just mitochondria (left panel) or mitochondria that co-localizes with cyclin E (right panel), suggesting that Pink1 mutants can't degrade their mitochondria in response to rapamycin. Following are the total number of cells quantified (n) for WT and Pink1-KO WTCT for DMSO and Rapamycin treated cells respectively: WT (without Cyclin E stain):618,518. WT (with cyclin E stain): 512,414. Pink1-KO WTCT (without Cyclin E stain):616,701. WT (with cyclin E stain): 502,569. (**G-H**) Images of wildtype and Pink1 homozygous null mutant clone 1 and 2 treated with either vehicle control (DMSO) or rapamycin (2μM) stained with DAPI (blue), CycE (red) and ATPsynβ (mitochondria, green) (Scale bar 5μm). The image with merged color ATPsynβ and CycE is

used in the main figure **7C**. Grey dashes represents the boundary of the cell of interest. (**I**) Representative 3D reconstructed OMX super resolution microscopy images of Pink1 homozygous null mutant clone 2 stained with DAPI (blue), CycE (red), and ATPsynβ (mitochondria, green) (Scale bar 0.3μm). Fig. A-C,E: wildtype =WTC. Fig. C-D,F-G: wildtype= WTC-Tom20.

SUPPLEMENTAL FIGURE 7: iPSCs require mitophagy to regulate cell cycle halt.
(**A**) Wildtype and PINK1-KD WTC cells stained with PH3 (mitosis marker, red), and DAPI (blue) (Scale bar 15μm). (**B**) Quantification of PH3 positive cells in wildtype vs PINK1-KD WTC treated with DMSO control vs 2μM rapamycin for 24 hours. Wildtype WTC shows decrease in positive PH3 cells by half when treated with rapamycin compared to DMSO control, whereas PINK1-KD WTC shows slightly higher PH3 positive cells when treated with rapamycin compared to wildtype WTC cells. Data is from triplicate experiments. Following are the total number of cells quantified (n) for WT and PINK1-KO WTCT for DMSO and Rapamycin treated cells respectively: Control WTCT: 3994, 3849. PINK1-KO WTCT: 3957, 3981. (**C**) Representative confocal microscopy images of wildtype WTCT or PINK1-KD mutant WTCT treated with either vehicle control (DMSO) or rapamycin (2μM) stained with DAPI (blue), PH3 (proliferating cell nuclei, red) (Scale bar 15μm). (**D**) Quantification of PH3 incidence in wildtype WTCT vs PINK1-KD mutant treated with either vehicle control (DMSO) or rapamycin (2μM), suggesting that PINK1 mutants fail to halt cell cycle progression as efficiently as wildtype. Following are the total number of cells quantified (n) for WT and PINK1-KO WTCT for DMSO and Rapamycin treated cells respectively: Control WTCT:3439,2760. PINK1-KO WTCT: 3162,3184. (**E**) Schematic of wildtype hiPSCs with or without reduced mitochondria (left panel) and its quantification (right panel). (**F**) Representative confocal microscopy images of PINK1 homozygous null mutant clone 2 treated with either vehicle control (DMSO) or rapamycin (2μM) stained with DAPI (blue), PH3 (proliferating cell nuclei, red) (Scale bar 25μm). (**G**) Representative FACS cell cycle analysis traces from wildtype WTC or PINK1 mutant WTC treated with vehicle (DMSO) or rapamycin (2μM) for 24 hours. Fig. A-B,G: wildtype =WTC. Fig. C-E: wildtype= WTC-Tom20.

SUPPLEMENTAL TABLES AND LEGENDS

SUPPLEMENTAL TABLE 1: Excel file compiling all IR paradigm source data relevant to the experiments.
Data are organized into sheets that they represent in the main and supplemental figures, followed by genotype, time points and replicate number.

SUPPLEMENTAL TABLE 2: Guide RNAs for PINK1

Gene Name	Guide RNA	Exon	Forward Oligo	Reverse Oligo
PINK1	GCAAGCGTCTC GTGTCCAAC	2	CACC**GCAAGCGTCTC GTGTCCAAC**	AAAC**GTTGGACACGAGACGC TTGC**

SUPPLEMENTAL TABLE 3: Primers for PINK1 gene

Oligo Name	Primer Sequence (Forward)	Primer Sequence (Reverse)
PINK1	GGCTGAGCAGTAGAACCTGG	CAGGCACCTTTCCTGTGGAT

SUPPLEMENTAL TABLE 4: siRNA for Cyclin E

Cyclin E siRNA	Cyclin E siRNA Pool Sequence
siRNA # 1	GUACUGAGCUGGGCAAAUA
siRNA # 2	UGUCCUGGCUGAAUGUAUA
siRNA # 3	GGACAAUAAUGCAGUCUGU
siRNA # 4	GGAGGUGUGUGAAGUCUAU

Details of number of GSCs analysed in main figures(1-5):

FIGURE 1: (G) Following are the total number of GSCs quantified (n) for each condition in order of Unirradiated, 1dpi and 2dpi time point: Control: 271, 276, 187. Raptor RNAi: 135,66, 60. Rictor RNAi 342,281,220. Tsc1RNAi:120,95,67), Nprl3 RNAi: 156, 152, 128. Mitf RNAi: 353, 328, 265.

FIGURE 2: (D) . Following are the total number of GSCs quantified (n) for each condition in order of Unirradiated, 1dpi and 2dpi time point: Control: 215,202,228. Atg1 OE 270,119,156. Atg14 RNAi: 274,214,235. Atg18a RNAi: 302,169,197. Atg3 RNAi: 196,164,221. Atg12 RNAi: 363,216,302. Atg16: 505,253,281.

FIGURE 3: (D) . Following are the total number of GSCs quantified (n) for each condition in order of Unirradiated, 1dpi and 2dpi time point: Control: 265,297,225. Pink1 RNAi: 379,214,202. Park RNAi: 394,202,264. Drp1 RNAi:235,205,157. Marf/Mfn2 RNAi: 203,193,186. Srl/PGC1a RNAi: 246,203,152.

FIGURE 4: (B) Following is the total number of GSCs quantified (n) for each condition in the order from unirradiated, 1dpi and to 2dpi time point: Control: 264, 264,238. Pc RNAi: 304,192,197. Sce RNAi: 193,191,129. Jarid2 RNAi: 135,128,103. Trx RNAi: 291,275,221. Utx RNAi: 277,252,232

FIGURE 5: (F) Following are the total number of GSCs quantified (n) for each condition in the order from unirradiated, 1dpi and to 2dpi time point: Control: 63,59,40. Tsc1 RNAi: 63, 56, 50. Pink1 RNAi: 68,63,47. Sce RNAi: 76,69,48. Jarid2 RNAi: 70,100,106. Srl/PGC1a RNAi: 64,59,51. Utx RNAi: 67,64,52.
