# Supplementary material for: Stress-induced reversible cell-cycle arrest requires PRC2/PRC1-mediated control of mitophagy in Drosophila germline stem cells and human iPSCs
Source: Stem Cell Reports. 2022 Dec 8;18(1):269–88. doi: 10.1016/j.stemcr.2022.11.004 (PMC9860083; doi:10.1016/j.stemcr.2022.11.004)
Supplement: Document S2. Article plus supplemental information [file mmc3.pdf]

Stem Cell Reports
Article

OPEN ACCESS

Stress-induced reversible cell-cycle arrest requires PRC2/PRC1-mediated control of mitophagy in *Drosophila* germline stem cells and human iPSCs

Tommy H. Taslim,[1,2,5] Abdiasis M. Hussein,[1,2,5] Riya Keshri,[1,2,5] Julien R. Ishibashi,[1,2,5] Tung C. Chan,[1,2] Bich N. Nguyen,[1,2] Shuozhi Liu,[1,2] Daniel Brewer,[1,2] Stuart Harper,[1,2] Scott Lyons,[1,2] Ben Garver,[1,2] Jimmy Dang,[1,2] Nanditaa Balachandar,[1,2,3] Samriddhi Jhajharia,[1,2,3] Debra del Castillo,[1,2] Julie Mathieu,[1,2,4] and Hannele Ruohola-Baker[1,2,*]
[1]Department of Biochemistry, University of Washington, Seattle, WA, USA
[2]Institute for Stem Cell and Regenerative Medicine, University of Washington, School of Medicine, Seattle, WA, USA
[3]Department of Biotechnology, School of Bioengineering, Faculty of Engineering and Technology, SRM Institute of Science and Technology, Kattankulathur, India
[4]Department of Comparative Medicine, University of Washington, Seattle, WA, USA
[5]These authors contributed equally
*Correspondence: hannele@uw.edu
https://doi.org/10.1016/j.stemcr.2022.11.004

SUMMARY

Following acute genotoxic stress, both normal and tumorous stem cells can undergo cell-cycle arrest to avoid apoptosis and later re-enter the cell cycle to regenerate daughter cells. However, the mechanism of protective, reversible proliferative arrest, "quiescence," remains unresolved. Here, we show that mitophagy is a prerequisite for reversible quiescence in both irradiated *Drosophila* germline stem cells (GSCs) and human induced pluripotent stem cells (hiPSCs). In GSCs, mitofission (Drp1) or mitophagy (Pink1/Parkin) genes are essential to enter quiescence, whereas mitochondrial biogenesis (PGC1α) or fusion (Mfn2) genes are crucial for exiting quiescence. Furthermore, mitophagy-dependent quiescence lies downstream of mTOR- and PRC2-mediated repression and relies on the mitochondrial pool of cyclin E. Mitophagy-dependent reduction of cyclin E in GSCs and in hiPSCs during mTOR inhibition prevents the usual G1/S transition, pushing the cells toward reversible quiescence (G0). This alternative method of G1/S control may present new opportunities for therapeutic purposes.

INTRODUCTION

Diverse types of stem cells have the capacity to exit the cell cycle upon stress, only to re-enter under the appropriate conditions; this process, coined "quiescence," is distinct from senescence because quiescence can normally be reversed. Nutrient-sensitive mechanistic target of rapamycin (mTOR) signaling has been implicated in quiescence, with mTOR activation promoting proliferation and exit from quiescence, and mTOR repression being a hallmark of stem cells in quiescence (van Velthoven and Rando, 2019; Cho et al., 2019; Meng et al., 2018; Artoni et al., 2017) and embryonic diapause (Bulut-Karslioglu et al., 2016; Hussein et al., 2020; Arena et al., 2021), an extreme example of developmental quiescence, with some exceptions (Mathieu et al., 2019). Moreover, quiescence is associated with decreased mitochondrial metabolism and increased macroautophagy (van Velthoven and Rando, 2019; Cho et al., 2019), herein referred to as autophagy. Epigenetic remodeling is another hallmark of quiescent stem cell states (van Velthoven and Rando, 2019; Cho et al., 2019; Hussein et al., 2020; Somasundaram et al., 2020; Hu et al., 2020). However, it remains unknown whether there are overarching rules that control entering and exiting quiescence across different types of stem cells. It will be particularly important to identify the molecules that distinguish reversible quiescence from DNA damage-induced apoptosis (Artoni et al., 2017; Xing et al., 2015).

DNA damage checkpoint recognizes irreparable DNA damage, inducing p53-dependent apoptosis (Aubrey et al., 2018; Speidel, 2010; Chakravarti et al., 2022; He et al., 2019). However, despite p53 activity (Hussein et al., 2020; Ma et al., 2016), stem cells in quiescence, including embryonic stem cells (ESCs) in diapause, resist apoptosis (Artoni et al., 2017; Hussein et al., 2020, 2022; Arena et al., 2021; Xing et al., 2015; Ma et al., 2016). Although it is not understood mechanistically how stem cells in general avoid apoptosis, in the germline stem cells (GSCs) of the adult *Drosophila* ovary at least one protective mechanism has been identified. GSCs in the somatic niche undergo self-renewing divisions to produce a cystoblast (CB) and a new GSC. The cystoblast further undergoes four incomplete cell divisions and eventually produces one oocyte connected to support cells called nurse cells; therefore, the GSCs are maintained in the somatic niche of the germaria to regenerate the oocyte pool. GSCs can survive genotoxic stress such as ionizing radiation (IR) by entering a reversible state of quiescence. In contrast, the GSC differentiating progenies undergo apoptosis and support survival of the GSCs (Xing et al., 2015). This reversible, protective GSC halt of the cell cycle allows regeneration of the germ line after insult (Artoni et al., 2017; Xing et al.,

This is an open access article under the CC BY license (http://creativecommons.org/licenses/by/4.0/).

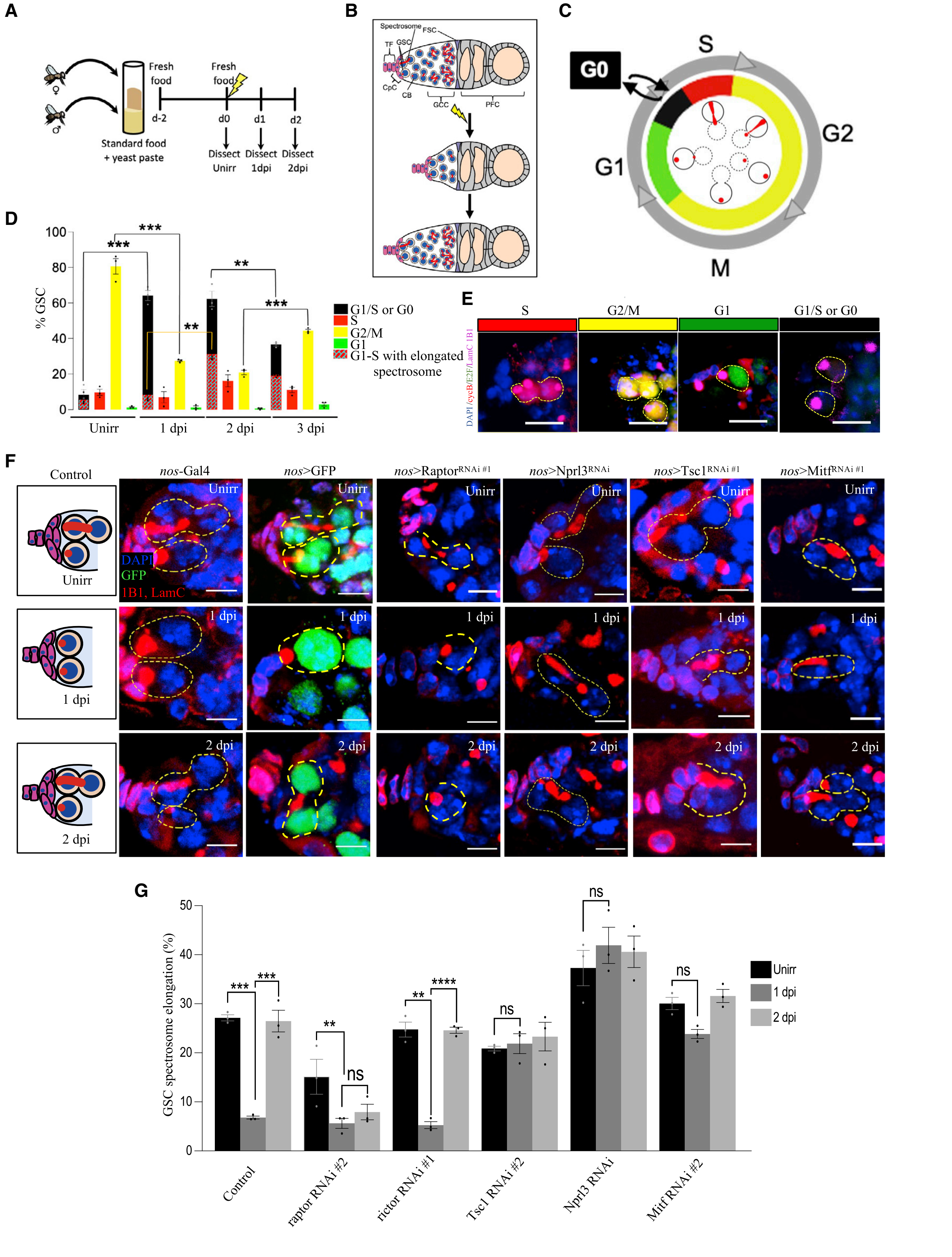

(legend on next page)

2015; Ma et al., 2016; Ishibashi et al., 2020, 2021) (Figures 1A and 1B). The stress-response transcription factor FOXO and the metabolic kinase mTOR were shown to be crucial for the entry into and exit from quiescence, respectively (Artoni et al., 2017). However, it is not yet known which mTOR targets are critical for the regulation of quiescence or how cell-cycle re-entry from GSC quiescence is so precisely timed.

In this study, we show that mTOR activity regulates the key epigenetic modifiers, PRC1/2, necessary for insult-induced mitophagy that results in quiescence. We further show that the mechanism of insult-induced quiescence relies on mitochondrial dynamics to temporally regulate a mitochondrial pool of cyclin E (CycE). Not only *Drosophila* GSCs, but also human induced pluripotent stem cells (hiPSCs) couple cell-cycle progression to mitochondrial quantity via the mitochondrial reserve of CycE.

RESULTS

mTORC1 inhibition is necessary for IR-induced entry into quiescence in *Drosophila* GSCs

To dissect the GSC reversible cell-cycle block in more detail we employed the IR-induced insult paradigm and fly fluorescent ubiquitination-based cell-cycle indicator (FUCCI) to visualize distinct cell-cycle stages in GSCs (Zielke et al., 2014) (Figures 1C–1E). In the fly FUCCI line GFP-$E2F1_{1-230}$ is degraded by ubiquitin ligase $CRL4^{Cdt2}$ in S phase, and mRFP-$CycB_{1-266}$ is degraded by APC/C in late mitosis/G1 (Figures 1C and 1E) (Zielke et al., 2014; Villa-Fombuena et al., 2021). Hence, FUCCI cells are green (GFP^+/RFP^-) in G1, red (GFP^-/RFP^+) in S, yellow (GFP^+/RFP^+) in G2/M, and black (GFP^-/RFP^-) in the G1-S transition (Figures 1C–1E). In the unirradiated control we observed a distribution of GSCs in (80%) G2/M, (8%) S, (1%) G1, and (8%) G1-S transition (Figures 1C and 1D), similar to previous findings (Villa-Fombuena et al., 2021). In contrast, 1 day post-insult (1 dpi) shows a significant increase in black GSCs (60%) (Figure 1D), a state of G0 quiescence. Further, 3 dpi shows a significant reduction in G0 with significant increase in G2/M (Figure 1D). At 2 dpi, when GSCs expectedly exit quiescence, we observe no significant reduction in black GSCs (Figure 1D). Upon analysis of the division marker phosphorylated serine 10 residue on histone 3 ($PH3^+$) in GSCs, we found that 2.3%, 0.3%, and 1.9% of GSCs are $PH3^+$ when unirradiated and at 1 and 2 dpi, respectively (Figure S1C), suggesting reversion of quiescence by 2 dpi (Figures S1B and S1C). To reconcile, we further analyzed another cell-cycle marker, GSC spectrosome elongation (Villa-Fombuena et al., 2021). The unirradiated GSCs showed an elongated spectrosome in S phase (or early G2) (Villa-Fombuena et al., 2021) (Figures 1C and 1F). At 1 dpi GSCs showed a significant reduction in elongated spectrosomes, whereas at 2 dpi the normal percentage of elongated spectrosomes was observed again, indicating the exit from quiescence (Figure 1F). Therefore, we proceeded to use elongated spectrosomes as a proxy for GSC division (Figures 1C, 1F, and 1G) (Artoni et al., 2017; Xing et al., 2015). We further categorized black GSCs in the FUCCI line into (1) unelongated, (2) elongated, or (3) partially elongated spectrosome type. When unirradiated, the rare black GSCs mainly showed partially elongated spectrosomes. At 1 dpi, black GSCs commonly have unelongated spectrosomes (75%) and partially elongated (25%) spectrosomes. Strikingly, at 2 dpi, we observed a significant increase in elongated spectrosomes, to 50% of black GSCs (Figures 1D and 1E), indicating G1-S transition (Figure 1C; Villa-Fombuena et al., 2021); hence, the GSCs appear to have mostly exited G0 or quiescence.

We sought to dissect the molecular role of the mTOR and its components to regulate quiescence. In wild-type (WT) control flies (*nos*-Gal4 driver), 27% of GSCs are in S phase

Figure 1. Role of mTORC1 in regulating insult-induced quiescence in female GSCs
(A) Experimental setup for irradiation model.
(B) Representative diagram of a germarium in the *Drosophila* ovary before and after IR. TF, terminal filament cell; CpC, cap cell; GSC, germline stem cell; CB, undifferentiated cystoblast; FSC, follicle stem cell; PFC, prefollicle cell.
(C) Schematic of various cell-cycle stages of GSCs, correlated with the expression pattern of GFP-$E2F1_{1-230}$ and mRFP-$CycB_{1-266}$ in GSCs.
(D) Percentage of cells positive for mRFP-$CycB_{1-266}$ (red) or GFP-$E2F1_{1-230}$ (green), dual positive mRFP-$CycB_{1-266}$ GFP-$E2F1_{1-230}$ (yellow), or expressing none of the fluorophore (black) under unirradiated, 1-day post-insult (1 dpi), 2 dpi, and 3 dpi conditions. Black represents G1/S or G0 phase, red represents S phase, yellow represents G2/M stage, and green represents G1 stage, while within the black bar the red dotted bar represents a percentage of cells in G1/S phase only, as these are the cells with elongated spectrosome.
(E) Representative images of GSCs in the fly FUCCI line expressing GFP-$E2F1_{1-230}$ and mRFP-$CycB_{1-266}$. Dotted circle represents GSC (scale bar, 5 μm).
(F) Representative confocal microscopy images of control (*nos*-Gal4 and *nos*>GFP) and listed UAS-RNAi KD from unirradiated, 1 dpi, and 2 dpi germaria stained with 1B1 (red, spectrosomes/fusomes), LamC (red, CpC and TF), and DAPI (blue, nuclei), as well as GFP (green, GSCs and progeny). Dotted circle represents GSC (scale bar, 5 μm).
(G) Percentage of GSCs showing spectrosome elongation.$^*p \leq 0.05$, $^{**}p \leq 0.01$, and $^{***}p \leq 0.001$.

with elongated spectrosomes (ESs; Figures 1F and 1G). At 1 dpi, the GSCs enter a state of quiescence (5% ES), and at 2 dpi GSCs successfully exit quiescence and resume cell division (27% ES; Figures 1F and 1G). Knockdown (KD) of the mTORC1 component raptor by RNAi moderately decreases rates of GSC division (12% ES) (Figures 1F and 1G), consistent with mTORC1 activity promoting cell division (Artoni et al., 2017). At 1 dpi the raptor KD GSCs efficiently enter IR-induced quiescence (2% ES), but strikingly, GSC division remains low at 2 dpi (3% ES), indicating a failure to exit quiescence (Figures 1F and 1G). In contrast, KD of the mTORC2 component rictor by RNAi has no appreciable effect on unirradiated rates of division (25% ES), and the GSCs properly arrest division at 1 dpi (5% ES) and resume division at 2 dpi (25% ES) (Figures 1G and S1A and Table S1), similar to WT controls, suggesting that mTORC2 might be functionally dispensable in GSC insult-induced quiescence. Altogether, these data show that mTORC1 activation is necessary for the exit from quiescence.

mTOR regulatory complexes TSC and GATOR1 are known to inhibit mTOR activity, while GATOR2 inhibits GATOR1 and can thereby activate mTORC1 (Kim and Guan, 2019; Wei et al., 2019). When the TSC component Tsc1 was knocked down by RNAi in GSCs, the rate of GSC division remained unchanged (unirradiated, 22% ES; 1 dpi, 21% ES; 2 dpi, 24% ES) (Figures 1F and 1G), suggesting that Tsc1 is required for quiescence. Similarly, when the GATOR1 component Nprl3 is knocked down, the GSCs do not block the cell cycle after insult (unirradiated, 37.5% ES; 1 dpi, 44.5% ES; 2 dpi, 43.5% ES) (Figures 1F and 1G). KD of another GATOR1 component, Nprl2, showed a similar inability to arrest cell division (Table S1), suggesting that GATOR1-mediated mTORC1 inhibition is essential for insult-induced quiescence. Intriguingly, GSCs with GATOR2 component (Nup44a and Mio) KD are comparable to WT controls (Table S1). These data are consistent with earlier findings that both TSC and GATOR1 become activated in response to the programmed DNA double-strand break during meiosis to mitigate genotoxic stress (Wei et al., 2019), and that GATOR2 is not appreciably active in GSCs (Wei et al., 2014). In conclusion, mTORC1 repression is essential to enter insult-induced quiescence in GSC.

During stress, mTORC1 inactivation results in transcription factor MITF/TFEB/TFE3 dephosphorylation, resulting in its nuclear translocation (Settembre et al., 2011). In *Drosophila* Mitf is the single identified MITF-TFE family member. Mitf can regulate V-ATPase expression that results in amino-acid-dependent activation of mTOR. mTORC1 then can phosphorylate and sequester *Drosophila* Mitf in the cytoplasm, leading to Mitf transcriptional inactivation through this feedback loop (Zhang et al., 2015). In addition, mammalian Mitf is shown to act in a multitude of other biological processes (Goding and Arnheiter, 2019). We sought to test the role of Mitf in *Drosophila* GSC IR-induced quiescence. Mitf KD abrogates insult-induced quiescence, as division rates at 1 dpi remain high (Figures 1F and 1G), suggesting that mTORC1 regulates GSC quiescence via Mitf-transcriptional targets, such as autophagy genes (Bouché et al., 2016).

Autophagy-deficient GSCs fail to enter into quiescence

We analyzed if mTORC1/Mitf-dependent autophagy regulates GSC quiescence. To characterize autophagy upon irradiation, we used the *nos*>mCherry-Atg8a (a reporter of autophagosome/autolysosome formation) line (Mauvezin et al., 2014). We observed a basal level of autophagy before irradiation, where 35% of GSCs contain mCherry-Atg8a puncta (Figures 2A and S2A–S2C). The number of GSCs with puncta increased at 1 dpi (87%) and reduced at 2 dpi (54%) (Figures 2A and S2A–S2C), indicating that autophagosomes/autolysosomes are acutely upregulated at GSC quiescence. To further characterize the rate of autophagic flux, we analyzed *nos*>GFP-mCherry-Atg8a GSCs. Unlike mCherry fluorescence, GFP is highly sensitive to pH, and therefore quenches in the low pH of the autolysosome (Mauvezin et al., 2014). Using this reporter, autophagosomes are expected to be yellow ($GFP^{+}/mCherry^{+}$), while autolysosomes are expected to be red ($GFP^{-}/mCherry^{+}$) (Figure S2A). Interestingly, we observed only red puncta, suggesting that, in GSCs, mature autophagosomes are rapidly acidified, characteristic of high autophagic flux (Figures 2A, 2B, and S2C). These data align with past results in hematopoietic stem cells suggesting that quiescence is characterized, in part, by the accumulation of autolysosomes (Liang et al., 2020).

Autophagy activation by either overexpression of Atg1, an inducer of autophagy, (Figures S2D and S2E), or KD of Rubicon, an inhibitor of autophagy (Table S1), prevented complete cell-cycle re-entry at 2 dpi, suggesting that dampening of autophagy is required to exit from quiescence. Atg1 has previously been reported to be phosphorylated by CDK1/cyclin B and is necessary for cell-cycle progression, which potentially explains the high basal division rate at 1 and 2 dpi in Atg1-overexpressing GSCs. Conversely, depletion of the core autophagy genes involved in phagophore nucleation, autophagic membrane elongation, or autophagosome maturation impaired the entrance into quiescence (Atg14, 18a, 3, 7, 12, 16; Figures 2C, 2D, and S2D and Table S1). These data align with findings that mouse ESCs arrest cell division upon chemical induction of autophagy (Suvorova et al., 2019), suggesting autophagy may be a more universal regulator of stem cell state.

Mitophagy acts downstream of IR-induced autophagy

Mitochondrial autophagy (mitophagy) and mitochondrial remodeling are important cellular processes that, when

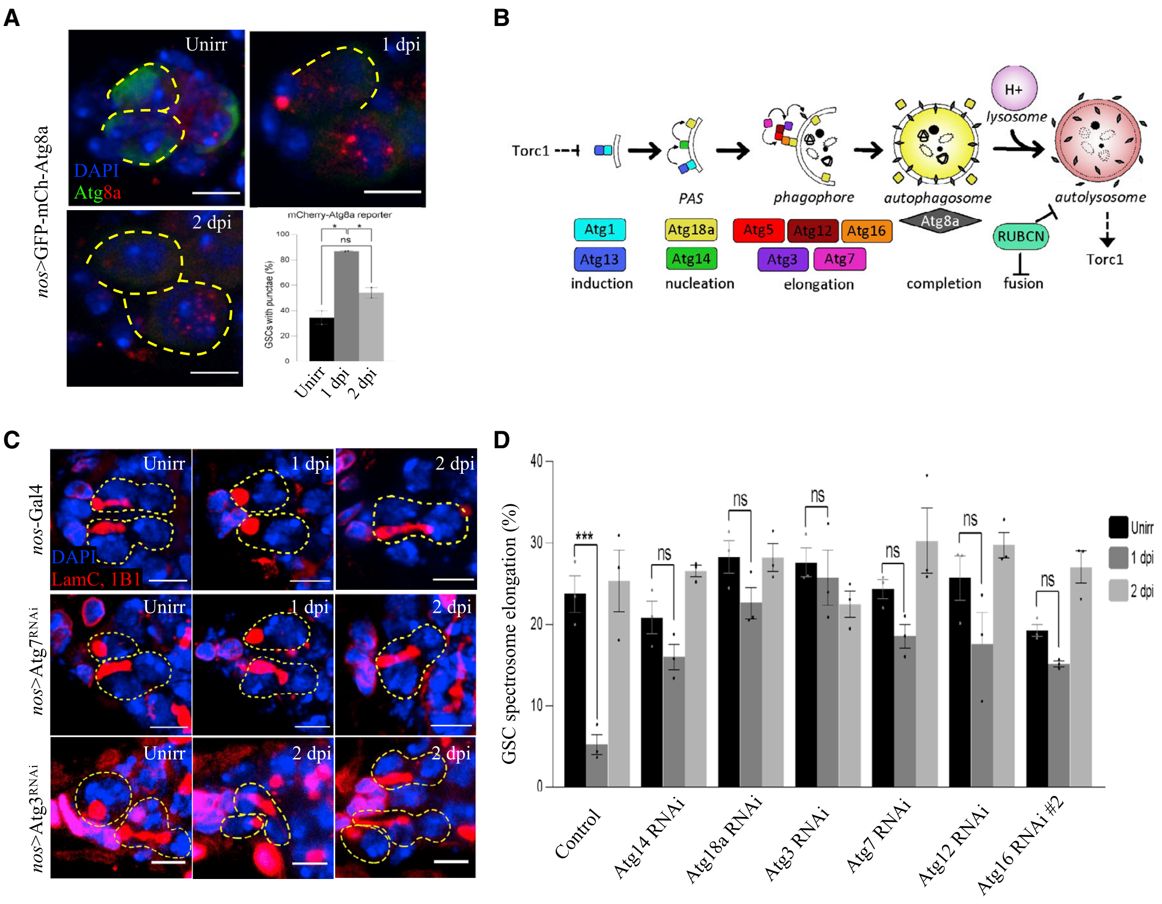

Figure 2. Autophagy-defective germline stem cells display impaired quiescence

(A) Representative confocal microscopy images of *nos*>GFP-mCherry-Atg8a from unirradiated, 1 dpi, and 2 dpi germaria stained with GFP (green, cytoplasm/autophagosome), mCherry (red, autophagosome/autolysosome), and DAPI (blue, nuclei). Dotted circle represents GSC (scale bars, 5 μm). The graph shows quantification of puncta in *nos*>mCherry-Atg8a germaria, stained with mCherry. A portion of unirradiated GSCs (35%) contain >1 mCherry puncta. At 1 dpi, this increases sharply (87%), suggesting an acute increase in autophagic degradation. By 2 dpi, GSCs with puncta decrease (54%), concurrent with the exit from quiescence.

(B) Schematic of known and hypothesized elements of interplay between autophagy and mTORC1.

(C) Immunofluorescence images of GSCs with respective overexpression or knockout of core components of autophagy proteins stained with 1B1 (red, spectrosomes/fusomes), LamC (red, CpCs and TFs), and DAPI (blue, nuclei). Dotted circle represents GSC (scale bars, 5 μm).

(D) Percentage of GSCs showing spectrosome elongation. $^*p \leq 0.05$ and $^{***}p \leq 0.001$.

defective, can result in disease states (Sun et al., 2016). We tested if the mTOR pathway in GSCs has the capacity to control mitophagy, as seen in another context (de la Cruz López et al., 2019). While the mitochondrial network of unirradiated WT GSCs was relatively fused and reticular, with increased mitochondrial density at the anterior end of the GSCs (Figures 3B and 5F) (Lieber et al., 2019; Cox and Spradling, 2003; Wang et al., 2016), at 1 dpi, most of the mitochondria were abnormal, 29% of GSCs with significantly decreased mitochondrial content (Figures 3B, 5A, 5F, and 5G). This decrease in mitochondrial content is dependent on autophagy, since Atg3 mutant GSCs display a normal anterior mitochondrial pattern at 1 dpi (Figures 5F, 5G, and S3M). Finally, at 2 dpi, the mitochondria returned to a more fused, anterior network, like the mitochondria of unirradiated GSCs (Figures 3B, 5A, 5F, and 5G). We show that Tsc1 RNAi mutant GSCs that lack quiescence (Figures 1F and 1G) also lack mitochondrial reduction at 1 dpi (Figures 5F, 5G, and S3L). These data reveal that GSC mitochondrial degradation and biogenesis coincide with mTOR-dependent entry into and exit from quiescence. This finding is strikingly like that in yeast, wherein proliferating yeast cells contain a tubular meshwork of mitochondria, while quiescent yeast cells have peripheral, fragmented mitochondria (Laporte et al., 2018), suggesting that mitochondrial morphology controls and/or responds to cell

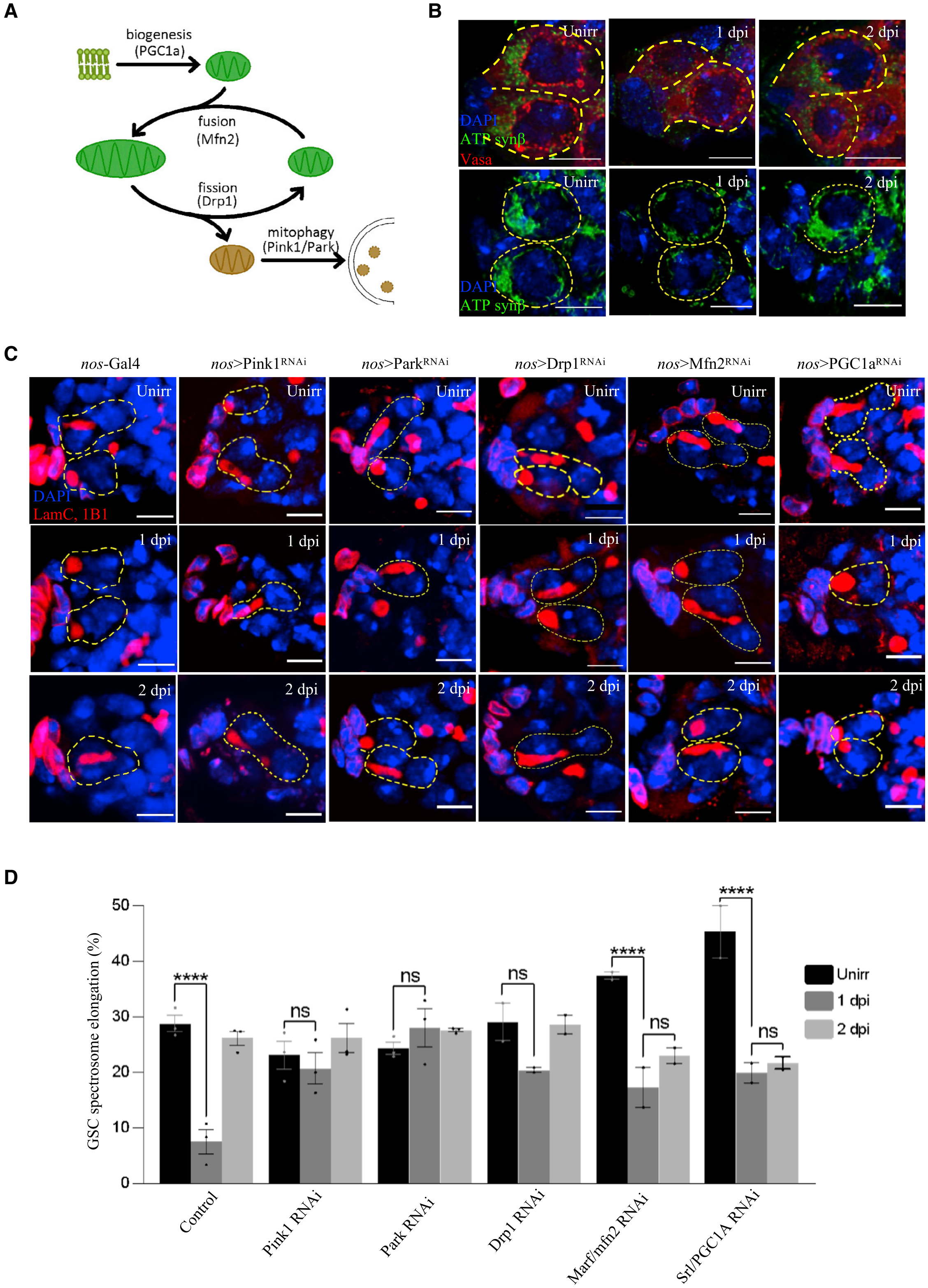

(legend on next page)

proliferation. We tested this hypothesis in stem cells by analyzing IR-induced quiescence in KD of the *Drosophila* mitophagy proteins, Pink1 and Parkin.

The Parkinson's disease genes Pink1 and Parkin mediate mitochondrial quality control mechanisms that culminate in the clearance of depolarized or dysfunctional mitochondria (Narendra et al., 2008; Matsuda et al., 2010; Vives-Bauza and Przedborski, 2010). We performed Pink1 and Parkin RNAi KD and found that the GSCs lacking Pink1 did not undergo mitochondrial degradation observed with WT GSCs at 1 dpi (Figures 5B, 5F, and 5G), arguing that mitochondria in normal GSCs after insult undergo mitophagy that requires Pink1 kinase activity. Furthermore, while the unirradiated Pink1 and Parkin KD GSCs divide at rates comparable to those of their WT counterparts (Figures 3C and 3D), Pink1 and Parkin KD GSCs still divide at 1 dpi (21% and 28%), failing to enter quiescence (Figures 3C and 3D). Using *nos*>mCherry-Atg8a, we further showed that Pink1-mediated mitophagy was downstream of IR-induced autophagy in GSCs; although Pink1 KD GSCs lacked mitophagy at 1 dpi, they still contained a significant increase in red puncta (Figure S3N). Our findings demonstrate that Pink1 and Parkin-mediated mitophagy is required for GSC IR-induced quiescence.

We also analyzed the canonical regulators of mitochondrial dynamics in GSC quiescence, which are known to interact with the mitophagy receptors Pink1/Parkin (Poole et al., 2010; Deng et al., 2008a, 2008b). We first analyzed the GTPase Dynamin-related protein, Drp1, which forms helical oligomers to pinch around the outer mitochondrial membrane and induce fission. Drp1 KD GSCs divided normally (29%) before irradiation but exhibited defects in arresting division at 1 dpi (20.5%) (Figures 3C and 3D), which implicates the role of mitofission (Figure 3A) in the entry into quiescence. Further, depletion of Marf/Mfn2, a GTPase responsible for mitochondrial fusion, showed a higher than normal division rate in unirradiated GSCs, and a reduced division at 1 dpi (>2-fold reduction), but failed to increase division by 2 dpi (Figures 3C and 3D). Similar to Mfn2 KD, mitochondrial biogenesis regulator PGC1α/srl KD GSCs also divided more than WT controls and significantly reduced division at 1 dpi (>2-fold reduction), but failed to increase division at 2 dpi (Figures 3C and 3D). These data show that PGC1α/srl KD GSCs undergo normal mitochondrial degradation at 1 dpi but fail to return to a more normal anterior accumulation of mitochondria at 2 dpi (Figures 5D, 5F, and 5G). In line with these data, in the fly FUCCI line, the black GSCs marked for quiescence at 1 dpi showed a significant reduction in mitochondria (Figure S1F). These data show that in GSCs, mitochondrial fission and mitophagy are required for the entry into quiescence, and mitochondrial fusion and biogenesis are required for the exit from quiescence.

Epigenetic modifiers are required for GSC entry into and exit from quiescence

The epigenome has been previously shown to be regulated by mitochondrial metabolites, and epigenomic changes have been identified in quiescent stem cell states (van Velthoven and Rando, 2019; Cho et al., 2019; Hussein et al., 2020; Somasundaram et al., 2020; Hu et al., 2020; Martínez-Reyes and Chandel, 2020; Moody et al., 2017; Levy et al., 2022). We now show that KD of the PRC1 (Pc/CBX and Sce/RING1) or PRC2 (Jarid2, Su(z)12, E(z), and Jing) components in GSCs abolishes the normal IR-induced cell-cycle block at 1 dpi, suggesting that these repressive epigenomic modifiers are required for entry into GSC quiescence (Figures 4A, 4B, and S3). Interestingly, some transcriptionally activating epigenetic modifiers were also required for quiescence entry, such as H3K10 kinase, Jil1(Deng et al., 2008a, 2008b); H3K79 methyltransferase, Gpp/DOT1L; and H3K4me2/3 methyltransferase, Set1 (Hallson et al., 2012) (Figures S3A–S3J and Table S1), suggesting that quiescence requires specific transcriptionally activating modifications. Additional enzymes responsible for recognizing DNA damage, γ-H2Av kinase and mei-41/ATR, are required for quiescence (Figures S3C and S3J and Table S1).

Conversely, we found another class of epigenetic modifiers that seem to be essential for the exit from quiescence. RNAi KD of Utx, an H3K27me3 demethylase, prevents GSCs from exiting quiescence (Figures 4A and 4B). An additional histone modifier, H3K4me1 methyltransferase Trx (Tie et al., 2014), also regulates GSC exit from quiescence (Figures 4A and 4B). Curiously, rhi, a piRNA pathway component and member of the heterochromatin protein 1 (HP1) family (Klattenhoff et al., 2009), likely regulates various aspects of GSC homeostasis and quiescence, as rhi RNAi KD causes low baseline division before irradiation

Figure 3. Mitochondrial remodeling events are required for proper coordination of quiescence

(A) Current model of mitochondria life cycle based on current literature.

(B) Representative confocal microscopy images of control GSCs from unirradiated, 1 dpi, and 2 dpi germaria stained with VASA (red), ATP synthase β subunit (ATPsynβ) (green, mitochondria), and DAPI (blue, nuclei) (top) or only ATPsynβ and DAPI (bottom). Scale bars, 5 μm.

(C) Representative confocal microscopy images of RNAi KD of mitochondrial fission (Drp1) and mitophagy (Pink1/Park) and mitochondrial fusion (Mfn2) and biogenesis (PGC1α) genes from unirradiated, 1 dpi, and 2 dpi germaria stained with 1B1 (red, spectrosomes/fusomes), LamC (red, CpCs and TFs), and DAPI (blue, nuclei). Dotted circle represents GSC (scale bars, 5 μm).

(D) Percentage of GSCs showing spectrosome elongation. ****p $\leq$ 0.0001.

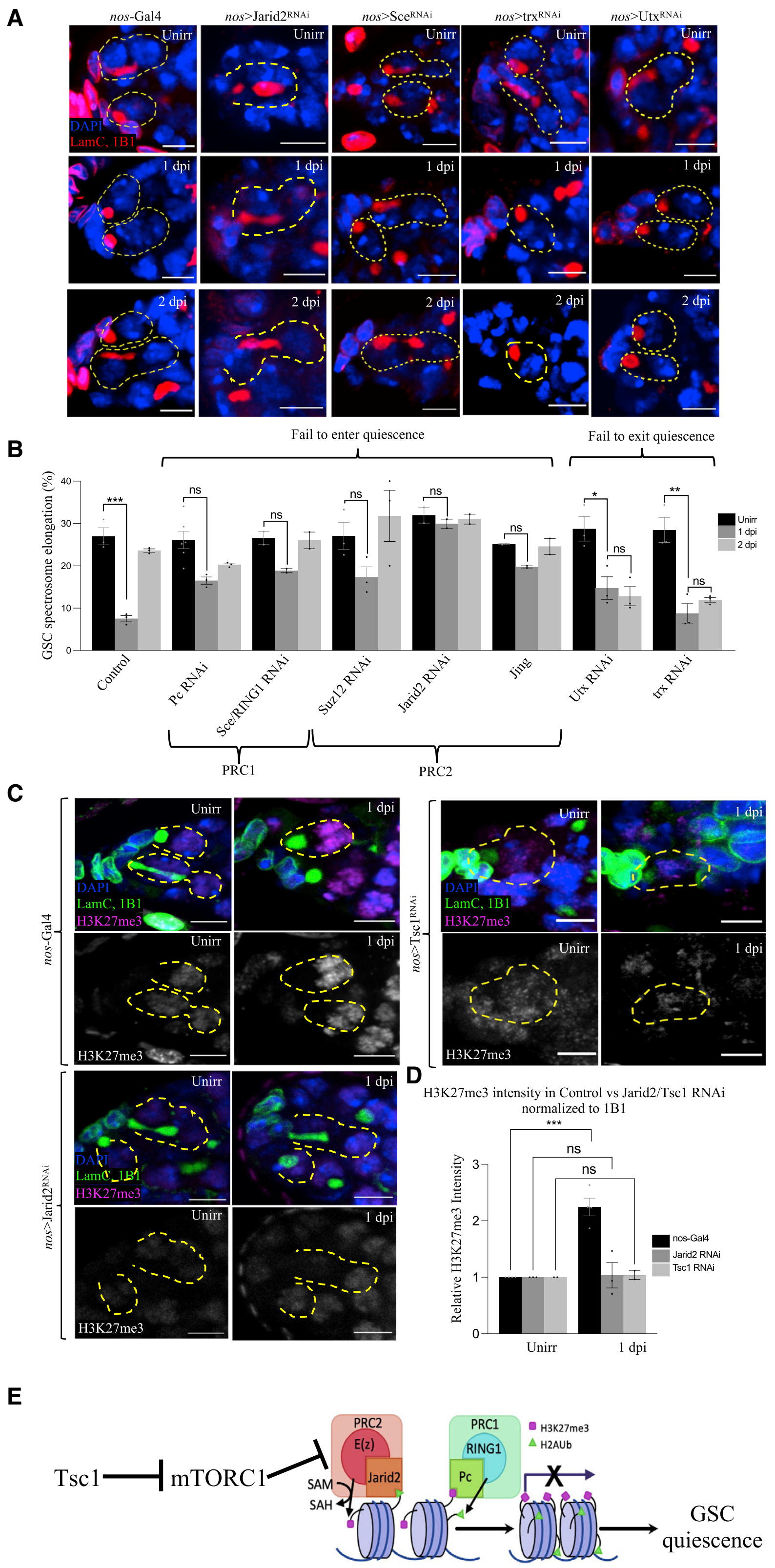

Figure 4. Epigenetic proteins also regulate GSC quiescence

(A) Representative confocal microscopy images of RNAi KD of various epigenetic regulatory genes from unirradiated, 1 dpi, and 2 dpi germaria stained with 1B1 (red, spectrosomes/fusomes), LamC (red, CpCs and TFs), and DAPI (blue, nuclei). Dotted circle represents GSC (scale bars, 5 μm).

(B) Percentage of GSCs showing spectrosome elongation.

(C) Representative confocal microscopy images of control, Jarid2-RNAi, and Tsc1-RNAi GSCs from unirradiated, 1 dpi, and 2 dpi germaria stained with LamC (green, CpCs and TFs), 1B1 (green, spectrosomes/fusomes), H3K27me3 (magenta), and DAPI (blue, nuclei). Dotted circle represents GSC (scale bars, 5 μm).

(D) Quantification of relative fluorescence intensity of H3K27me3 normalized to 1B1 intensity in control GSCs and Jarid2 RNAi GSCs, unirradiated and at 1 dpi.

(E) Upon irradiation, the canonical roles of PRC1 and PRC2 in epigenetic regulation during quiescence are shown. PRC2 binds to DNA by Jarid2, while E(z) consumes SAM to methylate H3K27, leading to transcriptional repression. PRC1 then binds to and recognizes existing H3K27me3 marks through CBX, and then catalyzes monoubiquitination of H2A, which further represses transcription and hence leads to quiescence. $^*p \leq 0.05$, $^{**}p \leq 0.01$, and $^{***}p \leq 0.001$.

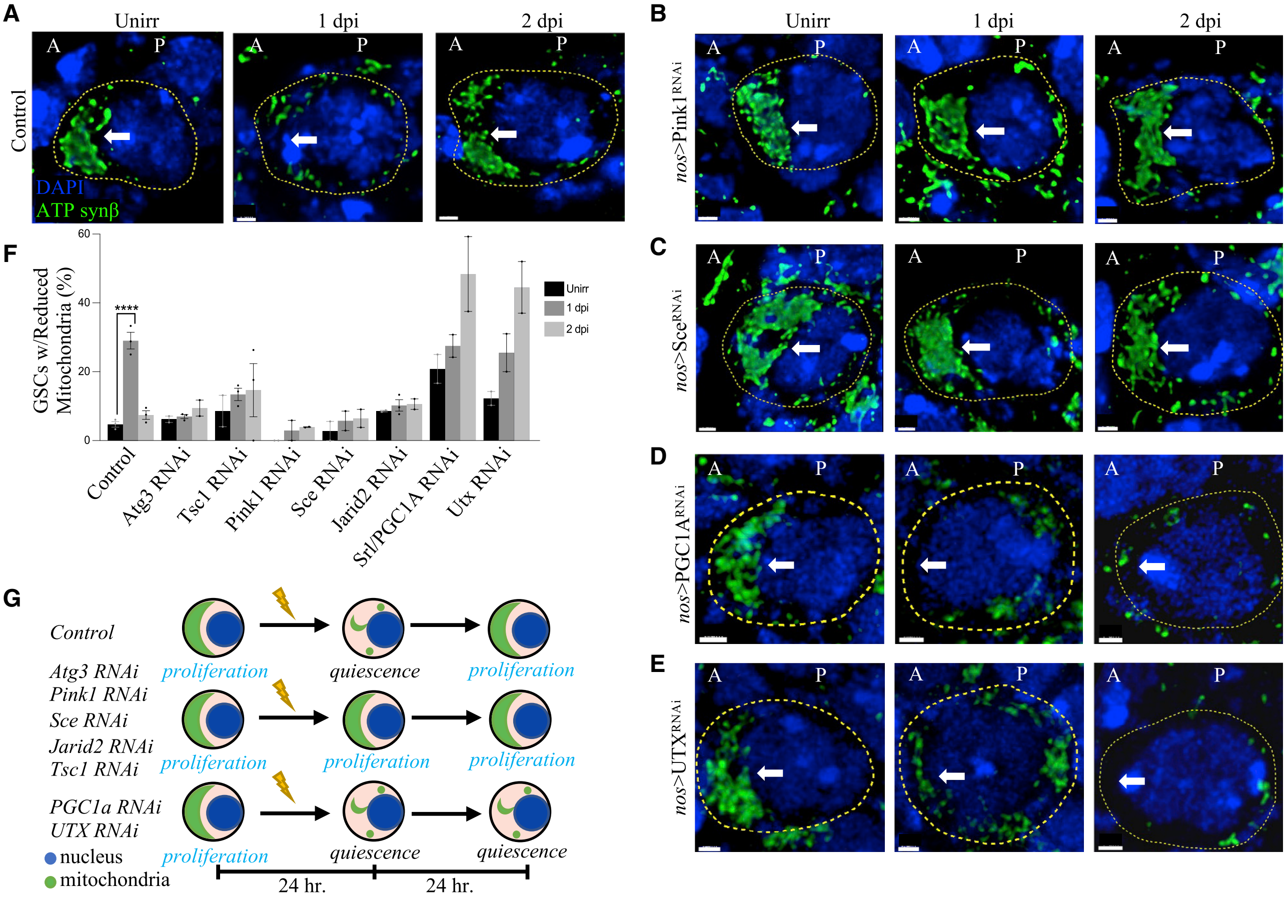

Figure 5. Mitophagy-dependent quiescence is mediated by PRC1

(A–E) Representative 3D reconstructed confocal microscopy images of GSCs and their respective RNAi KD line when unirradiated and at 1 and 2 dpi, stained with DAPI (blue, nuclei) and ATPsynβ (green, mitochondria) ("A" denotes anterior side, and "P" denotes the posterior side of the GSC). Arrow points to the area of interest, where mitochondria are typically clustered (scale bars, 1 μm).

(F) Quantification of incidence of reduced mitochondria where clustered mitochondria are not present in GSCs. Images from Figures S3K–S3M were used to generate this quantification.

(G) Proposed model by which mitochondria population acts as a checkpoint for the cell-cycle state of GSC. Blue represents nucleus and green represents mitochondria. ****$p \leq 0.0001$.

and impairs quiescence entry and exit at 1 and 2 dpi, respectively (Figures S3B and S3J and Table S1). We confirmed PRC1/2 activity in WT 1 dpi GSC nuclei by detecting a significant increase in H3K27me3 intensity compared with unirradiated nuclei (Figures 4C and 4D). In contrast, KD of the PRC2 component Jarid2, or mTOR regulator Tsc1, showed no significant difference in H3K27me3 levels at 1 dpi (Figures 4C and 4D). These data show that mTOR acts upstream of PRC1/PRC2 epigenetic modifiers that are required for GSC quiescence (Figure 4E).

Mitophagy-dependent quiescence is under dynamic epigenetic control

To test the relationship between mTOR, mitophagy, and epigenetic control of GSC IR-induced quiescence, we stained GSCs with ATPsynβ to analyze mitochondrial pattern and morphology in Tsc1, Pink1, Sce, PGC1/srl, and Utx RNAi mutants (Figures 5A–5F and S3K–S3L). As discussed above, at 1 dpi, the anterior mitochondrial network is lost in most of the WT GSCs, and the GSC ratio with dramatically reduced mitochondria is significantly increased. Using the fly FUCCI system we show that the observed mitochondrial reduction after insult occurs during the G0 stage of cell cycle (Figure S1F). The mitochondria morphology and pattern at 2 dpi are similar to those of unirradiated, representing exit from quiescence (Figures 5A, 5F, 5G, and S3K). In contrast, Tsc1 and Pink1 KD GSCs, which fail to enter quiescence after insult (Figures 1F, 1G, 3C, and 3D) showed the fewest changes in the mitochondrial network at 1 dpi (3%) (Figures 5B,

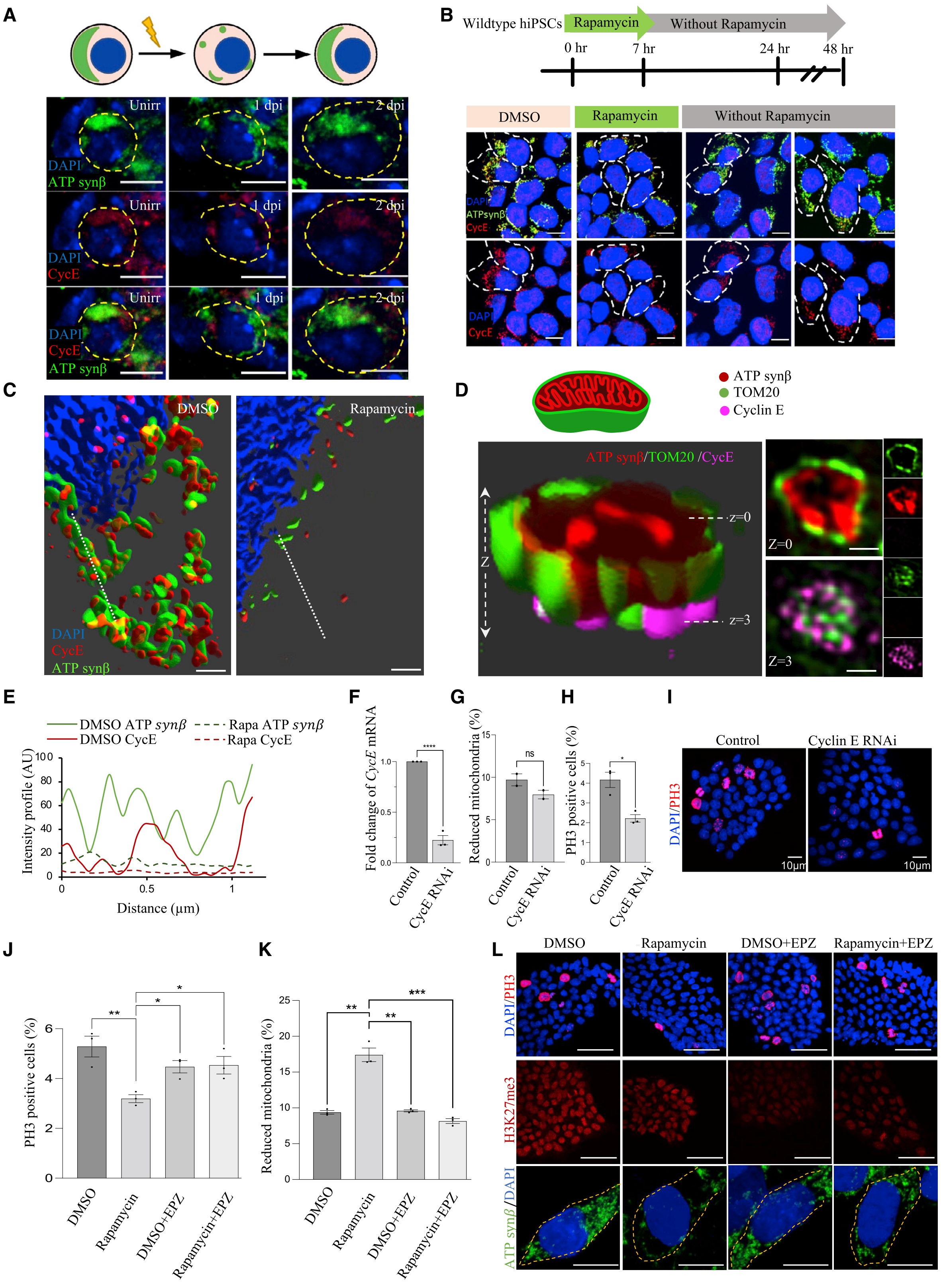

(legend on next page)

5F, S3K, and S3L), consistent with mTOR action upstream of mitophagy. Interestingly, epigenetic regulator Sce/RING1 KD GSCs, which fail to enter quiescence after insult (Figures 4A and 4B), also display low mitochondrial reduction (6%) at 1 dpi (Figures 5C, 5F, and S3K). Furthermore, Jarid2 RNAi mutants failed to detect IR-induced autophagy (Figure S3N). In contrast, PGC1 and Utx RNAi mutants continued to display segmented and highly reduced mitochondria at 2 dpi (Figures 5D–5F). These data suggest that PRC1/PRC2 and H3K27me3 demethylation are required for mitochondrial regulated quiescence and that the PRC1/PRC2-based epigenetic modifications act upstream of the mitophagy (Figure 5G).

Cyclin E localized to the mitochondria is degraded upon genotoxic or chemical insult in GSCs and hiPSCs

Since female *Drosophila* GSC division does not rely heavily on mitochondrial ATP (Teixeira et al., 2015), we tested the hypothesis that the mitochondria play a more direct role in cell-cycle progression. CycE, a G1-S regulator, has been reported to be targeted for degradation by ubiquitination by Parkin (Staropoli et al., 2003), the E3 ubiquitin ligase required for mitophagy and GSC quiescence (Figures 3C and 3D). Furthermore, Parkin mutations are associated with increased CycE in both cancer and Parkinson's neurons (Veeriah et al., 2010). This Parkin ubiquitination activity on CycE is regulated by a serine/threonine kinase, Pink1 (Ejma et al., 2020). While Parkin is involved in mitophagy and ubiquitin-dependent degradation of CycE, the connection between them remains unclear. Since recent work has observed some CycE co-localization with the mitochondria in both flies and mammals (Parker et al., 2015; Spurlock et al., 2020), we tested the hypothesis that Pink1-activated Parkin will be localized to mitochondria, where it initiates CycE degradation and mitophagy, resulting in quiescence.

In *Drosophila*, unirradiated GSCs show striking co-localization of CycE with the anteriorly localized, fused mitochondrial network (Figure 6A; Lieber et al., 2019; Cox and Spradling, 2003). However, at 1 dpi, the GSCs show a dramatic loss of quantity and anterior localization of both CycE and mitochondria, while at 2 dpi, CycE is once again observed co-localizing with the mitochondria, which are fused and anteriorly localized (Figures 6A and S3P). These data confirm that the mitochondria likely harbor a pool of CycE in GSCs and support the hypothesis that the mitochondrial content dictates cell-cycle progression after insult by regulating the CycE availability for G1-S transition.

To further understand whether the mTOR-regulated, p21 independent, irradiation-induced *Drosophila* GSC

Figure 6. Pool of cyclin E is observed on mitochondria in GSCs and hiPSCs

(A) Representative confocal microscopy images of *Drosophila* GSCs from unirradiated, 1 dpi, and 2 dpi germaria stained with ATPsynβ (mitochondria, green), cyclin E (red), and DAPI (blue) with cartoon schematic above (scale bars, 5 μm).

(B) Model shows the experimental design. Representative confocal microscopy images of WT hiPSCs treated with either vehicle control (DMSO) or rapamycin (2 μM) and pulse-chase stained with ATPsynβ (mitochondria, green), cyclin E (red), and DAPI (blue) (scale bars, 10 μm). hiPSCs shows reduction in both mitochondrial and cyclin E density compared with vehicle control after 7 h of 2 μM rapamycin treatment. Both mitochondria and cyclin E densities increase after 24 and 48 h post rapamycin treatment. Gray dashes represent the boundary of the cells of interest.

(C) Representative 3D-reconstructed OMX super-resolution microscopy images of WTC cells treated with vehicle control (DMSO) or 2 μM rapamycin for 24 h and stained with ATPsynβ (green), cyclin E (red), and DAPI (blue). The white dotted line was used to quantify the intensity profile in (E) (scale bars, 0.5 μm).

(D) A super-resolution 3D reconstruction of a mitochondrion in Tom20-GFP-expressing WTC, stained for ATPsynβ (red), GFP (green), and cyclin E (magenta). On the right, the two sections z = 0 and z = 3 show the mid-section and bottom of the mitochondrion (scale bars, 0.5 μm).

(E) Intensity profile of ATPsynβ (green) and CycE (red) in DMSO (solid line) and rapamycin (dotted line). The images in (C) were used to quantify the plot profile.

(F) Fold change of CycE analyzed in qPCR after knocking down CycE with siRNA for 72 h.

(G) Percentage of cells with reduced mitochondria in control or CycE siRNA for 72 h, n = 3, number of cells analyzed per condition >500.

(H) Percentage of cells positive for PH3 in control or CycE siRNA for 72 h, n = 3, number of cells analyzed per condition >500.

(I) Representative confocal microscopy images of wild-type hiPSCs transfected with either control or cyclin E siRNA for 72 h. DAPI is blue, PH3 is red (scale bars, 10 μm).

(J) Percentage of cells positive for PH3 in DMSO, 2 μM rapamycin, EPZ-6438, and rapamycin + EPZ-6438 for 24 h, n = 3, number of cells analyzed per condition >1,000.

(K) Percentage of cells with reduced mitochondria in DMSO, 2 μM rapamycin, EPZ-6438, and rapamycin + EPZ-6438 for 24 h, n = 3, number of cells analyzed per condition >500.

(L) Representative confocal images of cells treated with DMSO, 2 μM rapamycin, EPZ-6438, and rapamycin + EPZ-6438 for 24 h. Cells were stained for DAPI (blue), PH3 (red, scale bar, 50 μm), ATPsynβ (green, scale bar, 10 μm), and H3K27Me3 (red,scale bars, 50 μm). (B–E) wild type, WTC. (F–L) control, WTC-Tom20. *$p \leq 0.05$, **$p \leq 0.01$, ***$p \leq 0.001$, and ****$p \leq 0.0001$.

quiescence is conserved in mammalian stem cells, we irradiated hiPSCs and analyzed if the process resulted in mTOR inhibition (Qi et al., 2009; Zhan et al., 2019). Previous work has shown that phosphorylation and nuclear exit of the transcription factor TFE3 indicate mTOR activity in hiPSCs (Figure S4G; Mathieu et al., 2019). We therefore analyzed TFE3 nuclear localization in hiPSCs before and after irradiation (0.52 Gy of gamma-irradiation). As expected, TFE3 was mainly observed in the cytoplasm in control hiPSCs (Figures S4B and S4C). However, irradiation induced a 6-fold increase in nuclear TFE3 localization (1 dpi), suggesting that irradiation inhibits mTOR (Figure S4B). Concomitantly, irradiation induced a significant reduction in mitochondria in 80% of the hiPSCs (Figure S4A). These data suggest that irradiation inactivates mTOR and induces mitophagy also in hiPSC. Rapamycin treatment (mTOR inhibition) has previously been shown to induce quiescence (a diapause-like state) in pluripotent stem cells (Hussein et al., 2020). We, therefore, treated the hiPSCs with rapamycin as a surrogate of irradiation-induced mTORC1 inhibition and analyzed the effect of mitochondrial dynamics on CycE levels in quiescent hiPSC. We confirmed that rapamycin inhibits mTORC1 in hiPSCs (reduced pS6 within 7 h of treatment; Figure S4E). We found a stronger, but reversible inhibition of mTORC1 if rapamycin treatment was extended to 24 h (pS6 signal returns in 3 days after mTORC1 reactivation (Figures S4E and S4F). Twenty-four hours of rapamycin treatment also promoted significant nuclear localization of TFE3, confirming rapamycin efficiency in our experimental setup (Figure S4G). Importantly, rapamycin treatment resulted in a reversible reduction of epigenetic H4K16Ac marks (Figures S4E and S4F), indicating reduced cellular transcription previously seen in ESC diapause/quiescence (Hussein et al., 2020). These data suggest that hiPSCs can enter reversible quiescence after mTOR inhibition by rapamycin. Importantly, we observed a dramatic co-localization of CycE and mitochondria also in hiPSCs (Figures S4D, S4H, and S4I). The rapamycin-dependent co-localization of CycE and mitochondria was also reversible: while 7 h rapamycin treatment drastically decreased the patterns, after 24–48 h recovery, the mitochondria and CycE co-localization was similar to that of the vehicle controls (Figure 6B). These data confirm that mTORC1 inhibition in hiPSCs degrades both mitochondria and CycE, in a reversible manner.

Using super-resolution microscopy, we observed distinct co-localization of CycE with an outer mitochondrial membrane import receptor subunit, Tom20 (in the same mitochondrial region), but not with ATPsynβ, suggesting that the mitochondrial pool of CycE resides on the outer mitochondrial membrane (OMM) (Figure 6D). Upon rapamycin treatment (2 μM, 24 h) the mitochondria degraded, with a concomitant reduction in OMM CycE levels (Figures 6C and 6D). The reduction of mitochondrial CycE is proteasome dependent, since it can be reversed by treatment with a proteasome inhibitor (MG132; however, this does not rule out lysosomal degradation) (Figure S4M). To further test if CycE is critical for quiescence in iPSCs, we knocked down CycE (72 h; Figure 6F) and observed a significant decrease in PH3-positive cells, without altering mitochondrial reduction (Figures 6G–6I). This further implies that a reduced level of CycE is directly associated with stem cell entry into quiescence in hiPSCs.

Epigenetic control of hiPSC quiescence

When we treated hiPSCs with rapamycin to inhibit mTOR, and EPZ-6438 to inhibit EZH2, a component of the PRC2 complex, we confirmed PRC2 inhibition by a reduction in H3K27me3 (Figures 6L and S4K). In rapamycin treatment we observed a significant reduction in the number of $PH3^+$ cells, showing that rapamycin can induce a quiescence-like cell-cycle block in hiPSCs (Figures 6J and 6L). Further, we found that co-treatment of rapamycin with EPZ-6438 (Figure S4J) prevented hiPSCs from entering mTOR inhibition-dependent quiescence (Figures 6J and 6L). These data suggest that PRC2-mediated gene repression is essential for quiescence in hiPSCs and acts downstream of mTOR regulation. The control hiPSCs showed a significant increase in mitochondrial reduction in rapamycin-treated samples, while co-treatment of rapamycin and EPZ-6438 showed a lack of mitochondrial reduction (Figures 6K and 6L). This suggests that the state of quiescence induced through mTORC1 inhibition in hiPSCs is mediated by repressive histone marks, H3K27me3, which are upstream of mitochondrial degradation/fragmentation.

PINK1-mediated mitophagy dictates quiescence through cyclin E in hiPSCs

To further investigate the role of mitophagy in regulating the stability of CycE, we generated hiPSC lines with CRISPR-Cas9-induced mutations in the mitochondrial serine/threonine protein kinase PINK1. PINK1 contains an N-terminal mitochondrial localization sequence, a transmembrane sequence, a Ser/Thr kinase domain, and a C-terminal regulatory domain (Schubert et al., 2017) (Figure 7A). We engineered the CRISPR-Cas9 system to induce mutations prior to the kinase domain, thereby eliminating the PINK1 kinase activity (Figure 7A). We generated CRISPR mutants in two genetic backgrounds in which 90% of the cells generated had indels near the PAM region, resulting in dramatically reduced levels of PINK1 protein (Figures S5A and S5B). In addition to the KD, we generated two homozygous knockout lines with one base deletion that caused a frameshift and led to an early stop codon (Figures 7A, 7B, and S5A). The PINK1 knockout lines

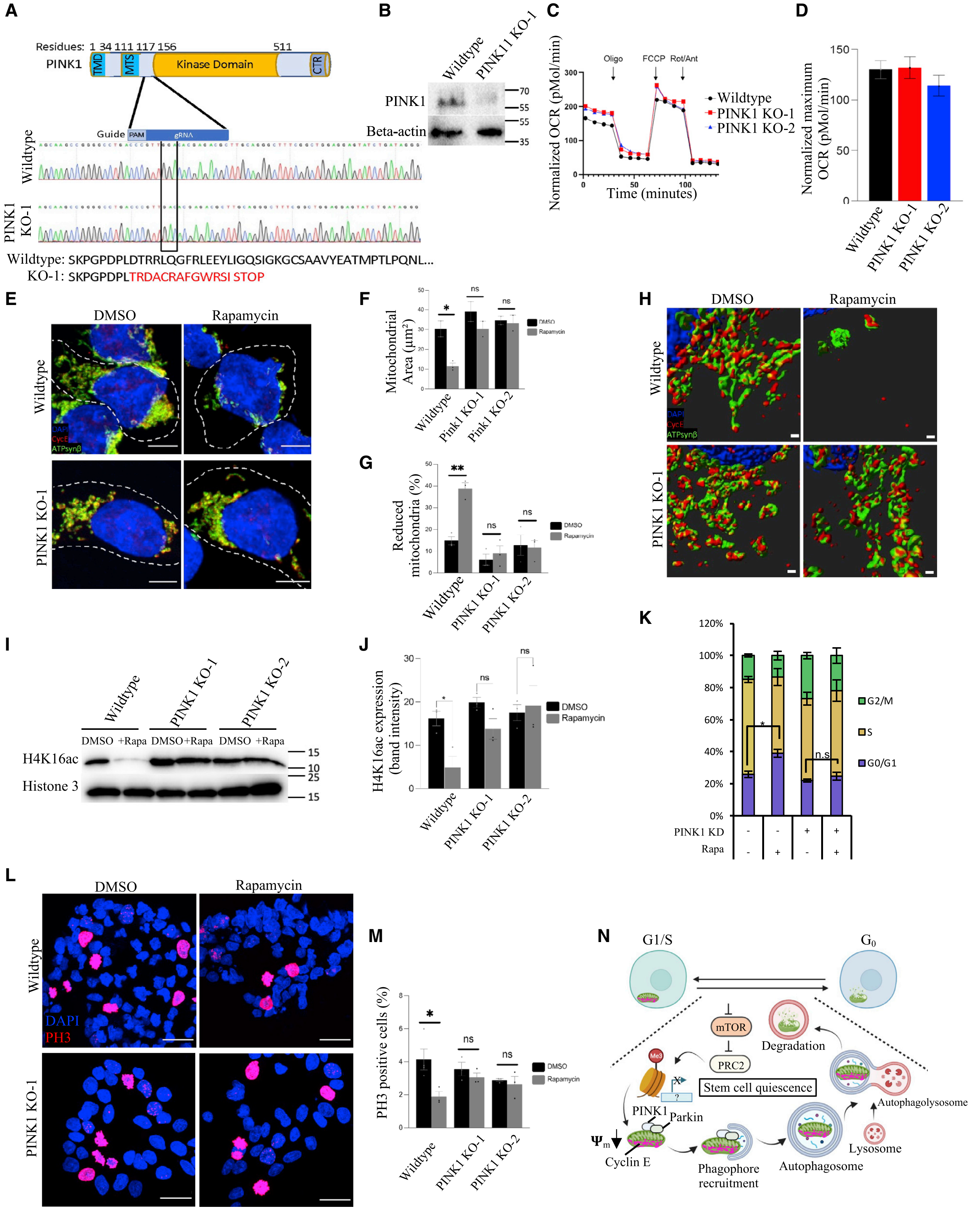

(legend on next page)

remain pluripotent and have the capacity to initiate differentiation into all three lineages similar to the WT iPSCs (Figures S5C–S5E). To characterize the metabolic profile of PINK1 knockout hiPSCs, we performed a mitostress assay to measure oxygen consumption rate (OCR) to determine the level of mitochondrial respiration using a Seahorse flux analyzer (Hussein et al., 2020; Sperber et al., 2015; Zhou et al., 2012; Miklas et al., 2019). The OCRs in response to FCCP (an uncoupling agent of mitochondrial oxidative phosphorylation) between WT and PINK1 knockout cells were indistinguishable (Figures 7C and 7D). We conclude that PINK1 KO cells are pluripotent and have a characteristic hiPSC metabolism.

We treated both control hiPSCs and PINK1 KD hiPSCs, as well as the two homozygous knockout hiPSCs, with rapamycin or vehicle for 24 h. As before (Figures S6B–S6D), control hiPSCs showed dense, fused mitochondrial networks, with only ~10% of cells exhibiting reduced mitochondria and CycE, but after 24 h of treatment with 2 μM rapamycin, mitochondrial and CycE levels were reduced significantly (Figures 7E–7H and S6A–S6G). In contrast, PINK1 mutant hiPSCs showed little to no mitochondrial or CycE reduction, regardless of whether they were treated with DMSO or rapamycin, suggesting that PINK1/Parkin-mediated mitophagy is required for OMM CycE degradation (Figures 7E–7H and S6A–S6I).

We show that rapamycin treatment downregulates H4K16Ac levels in WT, but not in PINK1 knockout cells (Figures 7I and 7J), indicating that PINK-dependent mitophagy is required for a transcriptionally silent iPSC quiescent stage. Moreover, using the $PH3^+$ mark to assess hiPSC proliferation, we found that rapamycin reduces cell division by up to 50% in control, but not in PINK1 mutant, hiPSCs (Figures 7L, 7M, S7A–S7D, and S7F). We also show that hiPSCs treated with vehicle (DMSO) for 24 h show a portion of cells in G0/G1 (25.5%), a large portion in S (58.4%), and the remainder in G2/M (14.7%) (Figures 7K and S7G). Conversely, after 24 h of rapamycin treatment, hiPSCs show significant G0/G1 arrest (38.7%), consistent with the idea that rapamycin and mTOR inhibition induces a diapause-like state of cellular quiescence in human hiPSCs. However, rapamycin-treated PINK1 mutant hiPSCs show no significant difference compared with vehicle-treated PINK1 mutant hiPSCs (Figures 7K and S7G). The inability of PINK1 mutant hiPSCs to arrest in G0 is consistent with mitophagy reducing CycE and therefore G1-S transition. These data indicate that hiPSCs, like *Drosophila* GSCs, utilize a non-canonical method of regulating the available reservoir of CycE via PINK1/PARKIN-mediated mitophagy. Together, these findings across stem cell types suggest that diverse stem cells may rely on mitochondrial count

Figure 7. Mitophagy in hiPSCs controls both mitochondrial cyclin E and cell cycle
(A) Pink1 structure with guide RNA location indicated and DNA sequencing chromatogram comparing wild-type Pink1 to mutant Pink1, showing a mixed pool of mutants and a loss of the wild-type sequence.
(B) Western blot showing Pink1 protein knocked out.
(C and D) Representative trace of OCR changes in response to oligomycin, FCCP, and rotenone/antimycin is shown under a MitoStress protocol (C). Pink1 KO cells show similar mitochondrial respiration pattern compared with wild-type Pink1 (D).
(E) Representative confocal microscopy images of wild-type or Pink1 mutant cells treated with either vehicle control (DMSO) or rapamycin (2 μM) and stained with DAPI (blue), CycE (red), and ATPsynβ (mitochondria, green) (scale bars, 5 μm).
(F and G) Quantification of (F) area of mitochondria and (G) mitochondrial degradation in wild type vs. Pink1 mutant treated with either vehicle control (DMSO) or rapamycin (2 μM). Following are the total numbers of cells quantified (n) for wild-type and Pink1-KO cells for DMSO- and rapamycin-treated cells, respectively: wild type (without cyclin E stain), 618, 518; wild type (with cyclin E stain), 512, 414; Pink1-KO cells (without cyclin E stain), 616, 701; wild type (with cyclin E stain), 502, 569.
(H) Representative 3D reconstructed OMX super-resolution microscopy images of wild-type or Pink1 mutant cells stained with DAPI (blue), CycE (red), and ATPsynβ (mitochondria, green) (scale bars, 0.2 μm).
(I) Representative western blot analysis of wild type, Pink1 KO-1, and Pink1 KO-2 treated with DMSO or 2 μM rapamycin for 24 h and blotted with H4K16Ac and histone 3 as a loading control.
(J) H4K16Ac quantification of the western blot in (I).
(K) Quantification of fluorescence-activated cell sorting (FACS) analysis of cell cycle by propidium iodide staining in wild type or Pink1KD treated with DMSO or rapamycin (2 μM).
(L) Representative confocal microscopy images of wild-type or Pink1 mutant cells treated with either vehicle control (DMSO) or rapamycin (2 μM) and stained with DAPI (blue) and PH3 (proliferating cell nuclei, red) (scale bars, 15 μm).
(M) Quantification of PH3 incidence in wild type vs. Pink1 mutant treated with either vehicle control (DMSO) or rapamycin (2 μM). Following are the total numbers of cells quantified (n) for wild type and Pink1-KO for DMSO- and rapamycin-treated cells, respectively: wild type, 3439, 2760; Pink1-KO, 3162, 3184.
(N) Model of proposed mechanism in which mitochondria can normally stabilize CycE and promote G1-S transition, while mitophagy induction will reduce CycE—perhaps through direct ubiquitination by Parkin—and keep the stem cells in quiescence/G0. (A–J, M–L) Wild type, WTC-Tom20; (K) wild type, WTC. $^*p \leq 0.05$ and $^{**}p \leq 0.01$.

to regulate available CycE and, consequently, stem cell quiescence.

DISCUSSION

In this study, we show that the commencement of quiescence in irradiated *Drosophila* GSCs and hiPSCs relies on the quantity of mitochondria (Figure 7N). We show that a failure in mitochondrial degradation abolishes quiescence, allowing a continuous cell cycle, whereas a failure in mitochondrial biogenesis or fusion impairs the exit from quiescence. Our data further suggest that mitochondrial quantity is controlled by the metabolic sensor mTORC1-dependent epigenetic regulation by PRC1/2. Predominantly, our research suggests that mitochondria mechanistically regulate the cell cycle by serving as a harbor for OMM CycE.

We found that the mTOR-responsive transcription factor Mitf and its downstream targets regulating autophagy are required for quiescence. Although autophagy has been shown to be necessary for stem cell quiescence in some contexts (García-Prat et al., 2016; Ho et al., 2017; Wang et al., 2018) and for proliferation in other contexts (Nagy et al., 2018; Salemi et al., 2012), our data unequivocally show that, in female *Drosophila* GSCs, autophagy is required for quiescence. Further, we report that the mitophagy effectors Pink1 and Parkin, as well as the mitofission protein Drp1, are each necessary for quiescence. Conversely, we found that the mitochondrial biogenesis transcription factor PGC1α and mitofusion protein Mfn2 are required to properly exit from quiescence. In conclusion, the mitochondrial mass tightly regulates reversible quiescence. Given the cross talk between mitophagy and mitochondrial dynamics (Lieber et al., 2019; Deng et al., 2008a, 2008b; Rana et al., 2017; Gong et al., 2015), it will be interesting to dissect whether mitophagy and mitochondrial fission are independent during quiescence, or if they always function in concert. Importantly, in human hiPSCs, we found that rapamycin-induced quiescence requires mitophagy and its regulator PINK1. In contrast, mTORC1 inhibition in mouse embryonic fibroblasts shows increased mitofusion (Morita et al., 2017). Hence, we conclude that mTORC-inhibition-dependent mitophagy might be a unique character of stem cells.

We show that mitophagy reduced mitochondrial CycE and increased G0 cell-cycle arrest in both GSCs and hiPSCs. Our finding that CycE decorates the OMM in hiPSCs raises the question of how CycE is trafficked between the OMM and the nucleus. We have identified a distinct mechanism whereby mitophagy destabilizes the critical G1/S cell cycle regulator, CycE, halting the cell cycle. While the CycE canonical regulator Dacapo/p21 can control DNA-damage-induced p53-dependent checkpoint in G0, p21/Dacapo does not appear to play a role in the GSC insult-induced quiescence (Artoni et al., 2017; Yu et al., 2009). We therefore argue that in stem cells, unexpectedly, the mitochondria quantity may be the primary regulator for CycE and act as a checkpoint for entering and exiting quiescence. It will be important to interrogate which stem cell types can utilize this alternative method of G1/S control, and whether this phenomenon can be leveraged for therapeutic purposes. perhaps utilizing AI-based protein design approach (Cao et al., 2022; Anishchenko et al., 2021).

In satellite stem cells, PcG promotes stemness and self-renewal (Margueron and Reinberg, 2011; Caretti et al., 2004; Bracken et al., 2006; Bianchi et al., 2020). In hematopoietic stem and progenitor cells, the PRC1 component Ring1 is required for self-renewal (Eskeland et al., 2010). Here, we show a novel role for the PcG proteins Pc/CBX8 and Sce/Ring1 of PRC1 as regulators of reversible, injury-induced GSC cell-cycle block. In line with our data, the Pc homolog Cbx7 in African killifish has been recently shown to be required for maintaining diapause (Hu et al., 2020). Furthermore, recent findings suggest that cancer-related irradiation and chemotherapy may lead a class of cancer cells (cancer stem cells, CSCs) to enter a protective, reversible diapause-like state (Dhimolea et al., 2021; Rehman et al., 2021; Hussein et al., 2022). Hence, targeting the PRC1/2 complex (Levy et al., 2022) might force cells to exit diapause-like quiescence in the normal, as well as the cancer, state, guiding CSCs to a proliferative stage amenable to conventional chemotherapies. Future work will be needed to examine whether insult-induced quiescence in GSCs requires Jarid2 via a Polycomb repressive element (PRE)-dependent mechanism like traditional *Drosophila* PcG proteins or via a PRE-independent mechanism like the default mammalian method (DeLuca et al., 2020; Herz et al., 2012; Landeira et al., 2010).

The cornerstone of stem cell quiescence in this study is PINK1/PARKIN-mediated mitophagy, which acts downstream of mTOR-dependent PRC2 complex activity (Figure 7N). It is plausible that the CycE pool associated with mitochondria gets directly ubiquitinated by Parkin, leading to an overall decrease in Cdk2/CycE activity essential for G1/S transition, resulting in a reversible G0 cell-cycle halt (Figure 7N). Future research will reveal whether manipulating mTORC1 signaling and mitochondria quantity could restore regenerative capacity to non-regenerative adult tissues like the heart (Miklas et al., 2022). Furthermore, it will be important to dissect in detail if and how mitochondrial dynamics play a role in GSC age-related senescence, as mitochondria have been shown to play a critical role in senescence in many cell types (Rana et al., 2017; Davalli et al., 2016; Scheckhuber et al., 2007; Chaudhari and Kipreos, 2017; Byrne et al., 2019; Pauklin and

Vallier, 2014; Tai et al., 2017). It will be important to investigate if similar epigenetic regulation of mitochondrial dynamics controls insult-induced quiescence in other stem cell types and whether senescence in aging stem cells can be reversed by altering mitochondrial dynamics.

EXPERIMENTAL PROCEDURES

Resource availability

Corresponding author

Further information and requests for resources and reagents should be directed to and will be fulfilled by the corresponding author, Hannele Ruohola-Baker (hannele@uw.edu).

Materials availability

All unique/stable reagents generated in this study are available from the corresponding author without restriction.

Data and code availability

Description of sample size and raw data are provided in the supplemental information. For further information, please contact the corresponding author.

Fly stocks and culture conditions

Flies were cultured at 25°C on cornmeal-yeast-agar medium supplemented with wet yeast (Artoni et al., 2017; Xing et al., 2015). Information regarding fly stocks is given in the supplemental experimental procedures.

hiPSC culture conditions

The hiPSCs were cultured on Matrigel growth factor-reduced basement membrane matrix (Corning) in mTeSR medium (STEMCELL Technologies). The cells were treated with rapamycin (200 nM–2 μM, Fisher Scientific) or DMSO for indicated periods and thereafter tested for reversion in the absence of rapamycin. To analyze the proteasome function, hiPSCs were treated with 100 nM MG132 (proteasomal inhibitor, Thermo Scientific; cat. no. 508339) for 24 h. Information regarding cell lines is given in the supplemental experimental procedures.

Ionizing radiation treatment in *Drosophila* and hiPSCs

Prior to gamma-irradiation exposure, 2- to 4-day-old flies (5–6 M:15–18 F) were kept on cornmeal-yeast-agar medium augmented with wet yeast for 48 h at 25°C. On the day of irradiation, two-thirds of the females and all males were transferred to empty plastic vials and treated with 50 Gy of gamma-irradiation. More details are provided in the supplemental experimental procedures. For irradiation in hiPSCs, we plated 5×10^4 hiPSCs in Matrigel-coated coverslip in 35 mm culture dishes. After 48 h, the dishes were treated with 0.52 Gy of gamma-irradiation. One day post-insult, the cells were fixed in paraformaldehyde and stained.

OCR measurement using Seahorse cellular flux assay

The Seahorse XF96 extracellular flux analyzer was used to assess mitochondrial function as described (Sperber et al., 2015; Zhou et al., 2012; Miklas et al., 2019). Details are given in the supplemental experimental procedures.

Drosophila and hiPSC immunofluorescence analysis

Fly samples and iPSCs were fixed and stained with different antibodies. Details regrading the immunofluorescence is given in the supplemental experimental procedures.

hiPSC gene knockout and knockdown

Guide RNAs (gRNAs) targeting exon 2 of PINK1 (Table S2) were designed using CRISPOR.org (Concordet and Haeussler, 2018) and inserted into a lentiCRISPR v.2 plasmid containing two expression cassettes, hSpCas9 and the chimeric guide RNA (Shalem et al., 2014; Sanjana et al., 2014) (a gift from Feng Zhang; Addgene plasmid 52961), as done before (Arena et al., 2021). The vector was digested using *BsmB*I, and a pair of annealed oligos were cloned into the single guide RNA scaffold. Sequences for the gRNAs and primers used for sequencing can be found in Tables S2 and S3, respectively.

For CycE KD, a million hiPSCs cells were seeded on a Matrigel-coated dish with mTeSR medium with a mixture of CycE siRNA (Dharmacon ON-target Plus SMARTpool siRNA) (Table S4) and RNAi Max (Invitrogen; 13778-075) prepared in Opti-MEM (Gibco; 51985).

hiPSC transduction and selection

hiPSCs were transduced with lentiCRISPR v.2/gRNA lentiviral particles targeting PINK1 with 4 μg/mL polybrene (Sigma Aldrich). Details are provided in the supplemental experimental procedures.

Trilineage differentiation

We differentiated the WT and PINK1 KO hiPSC lines using a STEMdiff Trilineage Differentiation Kit (STEMCELL Technologies; 05231, 05232, 05233). We performed qPCR for lineage-specific markers using a Human Pluripotent Stem Cell Trilineage Differentiation qPCR array kit (STEMCELL Technologies; 07515) and analyzed their differentiation efficiency using the software www.stemcell.com/qPCRanalysis.

Data and resources availability

Description of sample size, raw data, and additional information on experimental procedures are provided in the supplemental information. For further information, please contact the corresponding author.

SUPPLEMENTAL INFORMATION

Supplemental information can be found online at https://doi.org/10.1016/j.stemcr.2022.11.004.

AUTHOR CONTRIBUTIONS

Conceptualization, T.H.T., A.M.H., R.K., J.R.I., J.M., and H.R-B.; data curation, formal analysis, validation, and investigation, T.H.T., A.M.H., R.K., J.R.I., T.C.C., B.N.N., S. Liu, D.B., S.H., S. Lyons, B.G., J.D., N.B., S.J., D.D.C., and J.M.; methodology, T.H.T., A.M.H., R.K., J.R.I., and T.C.C.; visualization, T.H.T., A.M.H., R.K., J.R.I., S. Lyons, S.H., J.M., N.B., and S.J.; writing – original draft, T.H.T., J.R.I., and H.RB., writing – review & editing, T.H.T., A.M.H., R.K., J.R.I., S. Liu, J.M., and H.R-B.; resources, J.M.

and D.D.C.; funding acquisition, H.R.B.; project administration, H.R.B.

ACKNOWLEDGMENTS

We thank Prof. Helena Richardson for kindly providing *Drosophila* CycE antibodies. We thank Filippo Artoni, Arianne Caudal, Ondina Palmeira, Ellen Ward, Xiaosheng Yang, Blair Zhao, and other members of the Ruohola-Baker lab for their stimulating discussion and valuable comments. We thank Jennifer Hesson and Christopher Cavanaugh for their help. We thank the Lynn & Mike Garvey Imaging Core at the University of Washington and the Cell Analysis Facility Flow Cytometry Shared Resource Lab in the Department of Immunology at the University of Washington. This research used the ISCRM Ellison Stem Cell core facility expanded cell line made possible through the Allen Cell Collection, available from Coriell Institute for Medical Research. This work is supported by the Biological Mechanisms of Healthy Aging Training Program (NIH T32AG066574 to A.M.H.) and the Hahn Family and partly by grants from the American Heart Association (19IPL0134760143), Department of Defense (W81XWH2110007) and National Institutes of Health (R01GM097372, R01GM083867, 1P01GM081619, U01HL099997, and UO1HL099993 to H.R.-B.).

CONFLICT OF INTERESTS

The authors declare no competing interests.

Received: April 26, 2022
Revised: November 2, 2022
Accepted: November 7, 2022
Published: December 8, 2022

REFERENCES

Anishchenko, I., Pellock, S.J., Chidyausiku, T.M., Ramelot, T.A., Ovchinnikov, S., Hao, J., Bafna, K., Norn, C., Kang, A., Bera, A.K., et al. (2021). De novo protein design by deep network hallucination. Nature *600*, 547–552.

Arena, R., Bisogno, S., Gąsior, Ł., Rudnicka, J., Bernhardt, L., Haaf, T., Zacchini, F., Bochenek, M., Fic, K., Bik, E., et al. (2021). Lipid droplets in mammalian eggs are utilized during embryonic diapause. Proc. Natl. Acad. Sci. USA *118*. e2018362118.

Artoni, F., Kreipke, R.E., Palmeira, O., Dixon, C., Goldberg, Z., and Ruohola-Baker, H. (2017). Loss of foxo rescues stem cell aging in Drosophila germ line. Elife *6*, e27842.

Aubrey, B.J., Kelly, G.L., Janic, A., Herold, M.J., and Strasser, A. (2018). How does p53 induce apoptosis and how does this relate to p53-mediated tumour suppression? Cell Death Differ. *25*, 104–113.

Bianchi, A., Mozzetta, C., Pegoli, G., Lucini, F., Valsoni, S., Rosti, V., Petrini, C., Cortesi, A., Gregoretti, F., Antonelli, L., et al. (2020). Dysfunctional polycomb transcriptional repression contributes to lamin A/C-dependent muscular dystrophy. J Clin Invest *130*, 2408–2421.

Bouché, V., Espinosa, A.P., Leone, L., Sardiello, M., Ballabio, A., and Botas, J. (2016). Drosophila Mitf regulates the V-ATPase and the lysosomal-autophagic pathway. Autophagy *12*, 484–498.

Bracken, A.P., Dietrich, N., Pasini, D., Hansen, K.H., and Helin, K. (2006). Genome-wide mapping of Polycomb target genes unravels their roles in cell fate transitions. Genes Dev. *20*, 1123–1136.

Bulut-Karslioglu, A., Biechele, S., Jin, H., Macrae, T.A., Hejna, M., Gertsenstein, M., Song, J.S., and Ramalho-Santos, M. (2016). Inhibition of mTOR induces a paused pluripotent state. Nature *540*, 119–123.

Byrne, J.J., Soh, M.S., Chandhok, G., Vijayaraghavan, T., Teoh, J.S., Crawford, S., Cobham, A.E., Yapa, N.M.B., Mirth, C.K., and Neumann, B. (2019). Disruption of mitochondrial dynamics affects behaviour and lifespan in Caenorhabditis elegans. Cell. Mol. Life Sci. *76*, 1967–1985.

Cao, L., Coventry, B., Goreshnik, I., Huang, B., Sheffler, W., Park, J.S.W., and Jude, K.M. (2022). Design of protein-binding proteins from the target structure alone. Nature *605*, 551–560.

Caretti, G., Di Padova, M., Micales, B., Lyons, G.E., and Sartorelli, V. (2004). The Polycomb Ezh2 methyltransferase regulates muscle gene expression and skeletal muscle differentiation. Genes Dev. *18*, 2627–2638.

Chakravarti, A., Thirimanne, H.N., Brown, S., and Calvi, B.R. (2022). Isoforms have overlapping and distinct functions in germline genome integrity and oocyte quality control. Elife *11*, e61389.

Chaudhari, S.N., and Kipreos, E.T. (2017). Increased mitochondrial fusion allows the survival of older animals in diverse C. elegans longevity pathways. Nat. Commun. *8*, 182.

Cho, I.J., Lui, P.P., Obajdin, J., Riccio, F., Stroukov, W., Willis, T.L., Spagnoli, F., and Watt, F.M. (2019). Mechanisms, hallmarks, and implications of stem cell quiescence. Stem Cell Rep. *12*, 1190–1200.

Concordet, J.P., and Haeussler, M. (2018). CRISPOR: intuitive guide selection for CRISPR/Cas9 genome editing experiments and screens. Nucleic Acids Res. *46*, W242–W245.

Cox, R.T., and Spradling, A.C. (2003). A Balbiani body and the fusome mediate mitochondrial inheritance during Drosophila oogenesis. Development *130*, 1579–1590.

Davalli, P., Mitic, T., Caporali, A., Lauriola, A., and D'Arca, D. (2016). ROS, cell senescence, and novel molecular mechanisms in aging and age-related diseases. Oxid. Med. Cell. Longev. *2016*, 3565127.

DeLuca, S.Z., Ghildiyal, M., Pang, L.Y., and Spradling, A.C. (2020). Differentiating Drosophila female germ cells initiate Polycomb silencing by regulating PRC2-interacting proteins. Elife *9*, e56922.

Deng, H., Bao, X., Cai, W., Blacketer, M.J., Belmont, A.S., Girton, J., Johansen, J., and Johansen, K.M. (2008a). Ectopic histone H3S10 phosphorylation causes chromatin structure remodeling in Drosophila. Development *135*, 699–705.

de la Cruz López, K.G., Toledo Guzmán, M.E., Sánchez, E.O., and García Carrancá, A. (2019). mTORC1 as a regulator of mitochondrial functions and a therapeutic target in cancer. Front. Oncol. *9*, 1373.

Deng, H., Dodson, M.W., Huang, H., and Guo, M. (2008b). The Parkinson's disease genes pink1 and parkin promote mitochondrial fission and/or inhibit fusion in Drosophila. Proc. Natl. Acad. Sci. USA *105*, 14503–14508.

Dhimolea, E., de Matos Simoes, R., Kansara, D., Al'Khafaji, A., Bouyssou, J., Weng, X., Sharma, S., Raja, J., Awate, P., Shirasaki, R., et al. (2021). An embryonic diapause-like adaptation with suppressed myc activity enables tumor treatment persistence. Cancer Cell *39*, 240–256.e11.

Ejma, M., Madetko, N., Brzecka, A., Guranski, K., Alster, P., Misiuk-Hojło, M., Somasundaram, S.G., Kirkland, C.E., and Aliev, G. (2020). The links between Parkinson's disease and cancer. Biomedicines *8*, E416.

Eskeland, R., Leeb, M., Grimes, G.R., Kress, C., Boyle, S., Sproul, D., Gilbert, N., Fan, Y., Skoultchi, A.I., Wutz, A., and Bickmore, W.A. (2010). Ring1B compacts chromatin structure and represses gene expression independent of histone ubiquitination. Mol. Cell *38*, 452–464.

García-Prat, L., Martínez-Vicente, M., Perdiguero, E., Ortet, L., Rodríguez-Ubreva, J., Rebollo, E., Ruiz-Bonilla, V., Gutarra, S., Ballestar, E., Serrano, A.L., et al. (2016). Autophagy maintains stemness by preventing senescence. Nature *529*, 37–42.

Goding, C.R., and Arnheiter, H. (2019). MITF-the first 25 years. Genes Dev. *33*, 983–1007.

Gong, G., Song, M., Csordas, G., Kelly, D.P., Matkovich, S.J., and Dorn, G.W. (2015). Parkin-mediated mitophagy directs perinatal cardiac metabolic maturation in mice. Science *350*, aad2459.

Hallson, G., Hollebakken, R.E., Li, T., Syrzycka, M., Kim, I., Cotsworth, S., Fitzpatrick, K.A., Sinclair, D.A.R., and Honda, B.M. (2012). dSet1 is the main H3K4 di- and tri-methyltransferase throughout Drosophila development. Genetics *190*, 91–100.

He, F., Borcherds, W., Song, T., Wei, X., Das, M., Chen, L., Daughdrill, G.W., and Chen, J. (2019). Interaction between p53 N terminus and core domain regulates specific and nonspecific DNA binding. Proc. Natl. Acad. Sci. USA *116*, 8859–8868.

Herz, H.M., Mohan, M., Garrett, A.S., Miller, C., Casto, D., Zhang, Y., Seidel, C., Haug, J.S., Florens, L., Washburn, M.P., et al. (2012). Polycomb repressive complex 2-dependent and -independent functions of Jarid2 in transcriptional regulation in Drosophila. Mol. Cell Biol. *32*, 1683–1693.

Ho, T.T., Warr, M.R., Adelman, E.R., Lansinger, O.M., Flach, J., Verovskaya, E.V., Figueroa, M.E., and Passegué, E. (2017). Autophagy maintains the metabolism and function of young and old stem cells. Nature *543*, 205–210.

Hu, Z., Li, H., Jiang, H., Ren, Y., Yu, X., Qiu, J., Stablewski, A.B., Zhang, B., Buck, M.J., and Feng, J. (2020). Transient inhibition of mTOR in human pluripotent stem cells enables robust formation of mouse-human chimeric embryos. Sci. Adv. *6*, eaaz0298.

Hussein, A.M., Wang, Y., Mathieu, J., Margaretha, L., Song, C., Jones, D.C., Cavanaugh, C., Miklas, J.W., Mahen, E., Showalter, M.R., et al. (2020). Metabolic control over mTOR-dependent diapause-like state. Dev. Cell *52*, 236–250.e7.

Hussein, A.M., Balachandar, N., Mathieu, J., and Ruohola-Baker, H. (2022). Molecular regulators of embryonic diapause and cancer diapause-like state. Cells *11*, 2929.

Ishibashi, J.R., Keshri, R., Taslim, T.H., Brewer, D.K., Chan, T.C., Lyons, S., McManamen, A.M., Chen, A., Del Castillo, D., and Ruohola-Baker, H. (2021). Chemical genetic screen in *Drosophila* germline uncovers small molecule drugs that sensitize stem cells to insult-induced apoptosis. Cells *10*, 2771.

Ishibashi, J.R., Taslim, T.H., and Ruohola-Baker, H. (2020). Germline stem cell aging in the Drosophila ovary. Curr. Opin. Insect Sci. *37*, 57–62.

Kim, J., and Guan, K.L. (2019). mTOR as a central hub of nutrient signalling and cell growth. Nat. Cell Biol. *21*, 63–71.

Klattenhoff, C., Xi, H., Li, C., Lee, S., Xu, J., Khurana, J.S., Zhang, F., Schultz, N., Koppetsch, B.S., Nowosielska, A., et al. (2009). The Drosophila HP1 homolog Rhino is required for transposon silencing and piRNA production by dual-strand clusters. Cell *138*, 1137–1149.

Landeira, D., Sauer, S., Poot, R., Dvorkina, M., Mazzarella, L., Jørgensen, H.F., Pereira, C.F., Leleu, M., Piccolo, F.M., Spivakov, M., et al. (2010). Jarid2 is a PRC2 component in embryonic stem cells required for multi-lineage differentiation and recruitment of PRC1 and RNA Polymerase II to developmental regulators. Nat. Cell Biol. *12*, 618–624.

Laporte, D., Gouleme, L., Jimenez, L., Khemiri, I., and Sagot, I. (2018). Mitochondria reorganization upon proliferation arrest predicts individual yeast cell fate. Elife *7*, e35685. https://doi.org/10.7554/eLife.35685.

Levy, S., Somasundaram, L., Raj, I.X., Ic-Mex, D., Phal, A., Schmidt, S., Ng, W.I., Mar, D., Decarreau, J., Moss, N., et al. (2022). dCas9 fusion to computer-designed PRC2 inhibitor reveals functional TATA box in distal promoter region. Cell Rep. *38*, 110457.

Liang, R., Arif, T., Kalmykova, S., Kasianov, A., Lin, M., Menon, V., Qiu, J., Bernitz, J.M., Moore, K., Lin, F., et al. (2020). Restraining lysosomal activity preserves hematopoietic stem cell quiescence and potency. Cell Stem Cell *26*, 359–376.e7.

Lieber, T., Jeedigunta, S.P., Palozzi, J.M., Lehmann, R., and Hurd, T.R. (2019). Mitochondrial fragmentation drives selective removal of deleterious mtDNA in the germline. Nature *570*, 380–384.

Ma, X., Han, Y., Song, X., Do, T., Yang, Z., Ni, J., and Xie, T. (2016). DNA damage-induced Lok/CHK2 activation compromises germline stem cell self-renewal and lineage differentiation. Development *143*, 4312–4323.

Margueron, R., and Reinberg, D. (2011). The Polycomb complex PRC2 and its mark in life. Nature *469*, 343–349.

Martínez-Reyes, I., and Chandel, N.S. (2020). Mitochondrial TCA cycle metabolites control physiology and disease. Nat. Commun. *11*, 102.

Mathieu, J., Detraux, D., Kuppers, D., Wang, Y., Cavanaugh, C., Sidhu, S., Levy, S., Robitaille, A.M., Ferreccio, A., Bottorff, T., et al. (2019). Folliculin regulates mTORC1/2 and WNT pathways in early human pluripotency. Nat. Commun. *10*, 632.

Matsuda, N., Sato, S., Shiba, K., Okatsu, K., Saisho, K., Gautier, C.A., Sou, Y.S., Saiki, S., Kawajiri, S., Sato, F., et al. (2010). PINK1 stabilized by mitochondrial depolarization recruits Parkin to damaged mitochondria and activates latent Parkin for mitophagy. J. Cell Biol. *189*, 211–221.

Mauvezin, C., Ayala, C., Braden, C.R., Kim, J., and Neufeld, T.P. (2014). Assays to monitor autophagy in Drosophila. Methods *68*, 134–139.

Meng, D., Frank, A.R., and Jewell, J.L. (2018). mTOR signaling in stem and progenitor cells. Development *145*, dev152595.

Miklas, J.W., Clark, E., Levy, S., Detraux, D., Leonard, A., Beussman, K., Showalter, M.R., Smith, A.T., Hofsteen, P., Yang, X., et al. (2019). TFPa/HADHA is required for fatty acid beta-oxidation and cardiolipin re-modeling in human cardiomyocytes. Nat. Commun. *10*, 4671.

Miklas, J.W., Levy, S., Hofsteen, P., Mex, D.I., Clark, E., Muster, J., Robitaille, A.M., Sivaram, G., Abell, L., Goodson, J.M., et al. (2022). Amino acid primed mTOR activity is essential for heart regeneration. iScience *25*, 103574.

Moody, J.D., Levy, S., Mathieu, J., Xing, Y., Kim, W., Dong, C., Tempel, W., Robitaille, A.M., Dang, L.T., Ferreccio, A., et al. (2017). First critical repressive H3K27me3 marks in embryonic stem cells identified using designed protein inhibitor. Proc. Natl. Acad. Sci. USA *114*, 10125–10130.

Morita, M., Prudent, J., Basu, K., Goyon, V., Katsumura, S., Hulea, L., Pearl, D., Siddiqui, N., Strack, S., McGuirk, S., et al. (2017). mTOR controls mitochondrial dynamics and cell survival via MTFP1. Mol. Cell *67*, 922–935.e5.

Nagy, P., Sándor, G.O., and Juhász, G. (2018). Autophagy maintains stem cells and intestinal homeostasis in Drosophila. Sci. Rep. *8*, 4644.

Narendra, D., Tanaka, A., Suen, D.F., and Youle, R.J. (2008). Parkin is recruited selectively to impaired mitochondria and promotes their autophagy. J. Cell Biol. *183*, 795–803.

Parker, D.J., Iyer, A., Shah, S., Moran, A., Hjelmeland, A.B., Basu, M.K., Liu, R., and Mitra, K. (2015). A new mitochondrial pool of cyclin E, regulated by Drp1, is linked to cell-density-dependent cell proliferation. J. Cell Sci. *128*, 4171–4182.

Pauklin, S., and Vallier, L. (2014). The cell-cycle state of stem cells determines cell fate propensity. Cell *156*, 1338.

Poole, A.C., Thomas, R.E., Yu, S., Vincow, E.S., and Pallanck, L. (2010). The mitochondrial fusion-promoting factor mitofusin is a substrate of the PINK1/parkin pathway. PLoS One *5*, e10054.

Qi, J., Yu, J.Y., Shcherbata, H.R., Mathieu, J., Wang, A.J., Seal, S., Zhou, W., Stadler, B.M., Bourgin, D., Wang, L., et al. (2009). microRNAs regulate human embryonic stem cell division. Cell Cycle *8*, 3729–3741.

Rana, A., Oliveira, M.P., Khamoui, A.V., Aparicio, R., Rera, M., Rossiter, H.B., and Walker, D.W. (2017). Promoting Drp1-mediated mitochondrial fission in midlife prolongs healthy lifespan of Drosophila melanogaster. Nat. Commun. *8*, 448.

Rehman, S.K., Haynes, J., Collignon, E., Brown, K.R., Wang, Y., Nixon, A.M.L., Bruce, J.P., Wintersinger, J.A., Singh Mer, A., Lo, E.B.L., et al. (2021). Colorectal cancer cells enter a diapause-like DTP state to survive chemotherapy. Cell *184*, 226–242.e21.

Salemi, S., Yousefi, S., Constantinescu, M.A., Fey, M.F., and Simon, H.U. (2012). Autophagy is required for self-renewal and differentiation of adult human stem cells. Cell Res. *22*, 432–435.

Sanjana, N.E., Shalem, O., and Zhang, F. (2014). Improved vectors and genome-wide libraries for CRISPR screening. Nat. Methods *11*, 783–784.

Scheckhuber, C.Q., Erjavec, N., Tinazli, A., Hamann, A., Nyström, T., and Osiewacz, H.D. (2007). Reducing mitochondrial fission results in increased life span and fitness of two fungal ageing models. Nat. Cell Biol. *9*, 99–105.

Settembre, C., Di Malta, C., Polito, V.A., Garcia Arencibia, M., Vetrini, F., Erdin, S., Erdin, S.U., Huynh, T., Medina, D., Colella, P., et al. (2011). TFEB links autophagy to lysosomal biogenesis. Science *332*, 1429–1433.

Shalem, O., Sanjana, N.E., Hartenian, E., Shi, X., Scott, D.A., Mikkelson, T., Heckl, D., Ebert, B.L., Root, D.E., Doench, J.G., and Zhang, F. (2014). Genome-scale CRISPR-Cas9 knockout screening in human cells. Science *343*, 84–87.

Somasundaram, L., Levy, S., Hussein, A.M., Ehnes, D.D., Mathieu, J., and Ruohola-Baker, H. (2020). Epigenetic metabolites license stem cell states. Curr. Top. Dev. Biol. *138*, 209–240.

Speidel, D. (2010). Transcription-independent p53 apoptosis: an alternative route to death. Trends Cell Biol. *20*, 14–24.

Sperber, H., Mathieu, J., Wang, Y., Ferreccio, A., Hesson, J., Xu, Z., Fischer, K.A., Devi, A., Detraux, D., Gu, H., et al. (2015). The metabolome regulates the epigenetic landscape during naive-to-primed human embryonic stem cell transition. Nat. Cell Biol. *17*, 1523–1535.

Spurlock, B., Tullet, J., Hartman, J.L., and Mitra, K. (2020). Interplay of mitochondrial fission-fusion with cell cycle regulation: possible impacts on stem cell and organismal aging. Exp. Gerontol. *135*, 110919.

Staropoli, J.F., McDermott, C., Martinat, C., Schulman, B., Demireva, E., and Abeliovich, A. (2003). Parkin is a component of an SCF-like ubiquitin ligase complex and protects postmitotic neurons from kainate excitotoxicity. Neuron *37*, 735–749.

Sun, N., Youle, R.J., and Finkel, T. (2016). The mitochondrial basis of aging. Mol. Cell *61*, 654–666.

Suvorova, I.I., Knyazeva, A.R., Petukhov, A.V., Aksenov, N.D., and Pospelov, V.A. (2019). Resveratrol enhances pluripotency of mouse embryonic stem cells by activating AMPK/Ulk1 pathway. Cell Death Discov. *5*, 61.

Tai, H., Wang, Z., Gong, H., Han, X., Zhou, J., Wang, X., Wei, X., Ding, Y., Huang, N., Qin, J., et al. (2017). Autophagy impairment with lysosomal and mitochondrial dysfunction is an important characteristic of oxidative stress-induced senescence. Autophagy *13*, 99–113.

Teixeira, F.K., Sanchez, C.G., Hurd, T.R., Seifert, J.R.K., Czech, B., Preall, J.B., Hannon, G.J., and Lehmann, R. (2015). ATP synthase promotes germ cell differentiation independent of oxidative phosphorylation. Nat. Cell Biol. *17*, 689–696.

Tie, F., Banerjee, R., Saiakhova, A.R., Howard, B., Monteith, K.E., Scacheri, P.C., Cosgrove, M.S., and Harte, P.J. (2014). Trithorax monomethylates histone H3K4 and interacts directly with CBP to promote H3K27 acetylation and antagonize Polycomb silencing. Development *141*, 1129–1139.

van Velthoven, C.T.J., and Rando, T.A. (2019). Stem cell quiescence: dynamism, restraint, and cellular idling. Cell Stem Cell *24*, 213–225.

Veeriah, S., Taylor, B.S., Meng, S., Fang, F., Yilmaz, E., Vivanco, I., Janakiraman, M., Schultz, N., Hanrahan, A.J., Pao, W., et al. (2010). Somatic mutations of the Parkinson's disease-associated

gene PARK2 in glioblastoma and other human malignancies. Nat. Genet. *42*, 77–82.

Villa-Fombuena, G., Lobo-Pecellín, M., Marín-Menguiano, M., Rojas-Ríos, P., and González-Reyes, A. (2021). Live imaging of the Drosophila ovarian niche shows spectrosome and centrosome dynamics during asymmetric germline stem cell division. Development *148*, dev199716. https://doi.org/10.1242/dev.199716.

Vives-Bauza, C., and Przedborski, S. (2010). PINK1 points Parkin to mitochondria. Autophagy *6*, 674–675.

Wang, L., Shang, Z., Zhou, Y., Hu, X., Chen, Y., Fan, Y., Wei, X., Wu, L., Liang, Q., Zhang, J., and Gao, Z. (2018). Autophagy mediates glucose starvation-induced glioblastoma cell quiescence and chemoresistance through coordinating cell metabolism, cell cycle, and survival. Cell Death Dis. *9*, 213.

Wang, Z.H., Clark, C., and Geisbrecht, E.R. (2016). Drosophila clueless is involved in Parkin-dependent mitophagy by promoting VCP-mediated Marf degradation. Hum. Mol. Genet. *25*, 1946–1964.

Wei, Y., Bettedi, L., Ting, C.Y., Kim, K., Zhang, Y., Cai, J., and Lilly, M.A. (2019). The GATOR complex regulates an essential response to meiotic double-stranded breaks in. Elife *8*, e42149. https://doi.org/10.7554/eLife.42149.

Wei, Y., Reveal, B., Reich, J., Laursen, W.J., Senger, S., Akbar, T., Iida-Jones, T., Cai, W., Jarnik, M., and Lilly, M.A. (2014). TORC1 regulators Iml1/GATOR1 and GATOR2 control meiotic entry and oocyte development in Drosophila. Proc. Natl. Acad. Sci. USA *111*, E5670–E5677. https://doi.org/10.1073/pnas.1419156112.

Xing, Y., Su, T.T., and Ruohola-Baker, H. (2015). Tie-mediated signal from apoptotic cells protects stem cells in Drosophila melanogaster. Nat. Commun. *6*, 7058. https://doi.org/10.1038/ncomms8058.

Yu, J.Y., Reynolds, S.H., Hatfield, S.D., Shcherbata, H.R., Fischer, K.A., Ward, E.J., Long, D., Ding, Y., and Ruohola-Baker, H. (2009). Dicer-1-dependent Dacapo suppression acts downstream of Insulin receptor in regulating cell division of Drosophila germline stem cells. Development *136*, 1497–1507. https://doi.org/10.1242/dev.025999.

Zhan, Z., Song, L., Zhang, W., Gu, H., Cheng, H., Zhang, Y., Yang, Y., Ji, G., Feng, H., Cheng, T., and Li, Y. (2019). Absence of cyclin-dependent kinase inhibitor p27 or p18 increases efficiency of iPSC generation without induction of iPSC genomic instability. Cell Death Dis. *10*, 271. https://doi.org/10.1038/s41419-019-1502-8.

Zhang, T., Zhou, Q., Ogmundsdottir, M.H., Möller, K., Siddaway, R., Larue, L., Hsing, M., Kong, S.W., Goding, C.R., Palsson, A., et al. (2015). Mitf is a master regulator of the v-ATPase, forming a control module for cellular homeostasis with v-ATPase and TORC1. J. Cell Sci. *128*, 2938–2950. https://doi.org/10.1242/jcs.173807.

Zhou, W., Choi, M., Margineantu, D., Margaretha, L., Hesson, J., Cavanaugh, C., Blau, C.A., Horwitz, M.S., Hockenbery, D., Ware, C., and Ruohola-Baker, H. (2012). HIF1α induced switch from bivalent to exclusively glycolytic metabolism during ESC-to-EpiSC/hESC transition. EMBO J. *31*, 2103–2116. https://doi.org/10.1038/emboj.2012.71.

Zielke, N., Korzelius, J., van Straaten, M., Bender, K., Schuhknecht, G.F.P., Dutta, D., Xiang, J., and Edgar, B.A. (2014). Fly-FUCCI: a versatile tool for studying cell proliferation in complex tissues. Cell Rep. *7*, 588–598. https://doi.org/10.1016/j.celrep.2014.03.020.

Stem Cell Reports, Volume *18*

Supplemental Information

Stress-induced reversible cell-cycle arrest requires PRC2/PRC1-mediated control of mitophagy in *Drosophila* germline stem cells and human iPSCs

Tommy H. Taslim, Abdiasis M. Hussein, Riya Keshri, Julien R. Ishibashi, Tung C. Chan, Bich N. Nguyen, Shuozhi Liu, Daniel Brewer, Stuart Harper, Scott Lyons, Ben Garver, Jimmy Dang, Nanditaa Balachandar, Samriddhi Jhajharia, Debra del Castillo, Julie Mathieu, and Hannele Ruohola-Baker

SUPPLEMENTAL EXPERIMENTAL PROCEDURES:

Fly stocks and culture conditions:

The following stocks were obtained from the Bloomington *Drosophila* Stock Center at Indiana University: w^{1118} (RRID:BDSC_3605), UAS-Dcr2, w^{1118}; nos-Gal4 (RRID:BDSC_25751), w*;;nos-Gal4 (RRID:BDSC_25394), w*; UAS-GFP (RRID:BDSC_6874), UAS-Tsc1$^{RNAi\ \#1}$ (RRID:BDSC_35144), UAS-Tsc1$^{RNAi\#2}$ (RRID:BDSC_54034), (RRID:BDSC_33914), UAS-Cnc^{RNAi} (RRID:BDSC_40854), UAS-Raptor$^{RNAi\#1}$ (RRID:BDSC_41912), UAS-Raptor$^{RNAi\#2}$ (RRID:BDSC_34814),UAS-Nprl3^{RNAi} (RRID:BDSC_55384), UAS-Mitf$^{RNAi\#1}$ (RRID:BDSC_44561), UAS-Mitf$^{RNAi\#2}$ (RRID:BDSC_43998), UAS-Nup44^{RNAi} (RRID:BDSC_39357), UAS-Mio^{RNAi} (RRID:BDSC_57745), UAS-Rictor^{RNAi} (RRID:BDSC_36699), UAS-Rictor^{RNAi} (RRID:BDSC_36584), UAS-Nprl2^{RNAi} RRID:BDSC_57538), UASp-mCherry-Atg8a (RRID:BDSC_37750), UASp-GFP-mCherry-Atg8a (RRID:BDSC_37749), UAS-Atg3^{RNAi} RRID:BDSC_34359), UAS-Atg7^{RNAi} (RRID:BDSC_34369), UAS-Atg5^{RNAi} (RRID:BDSC_34899), UAS-Atg12^{RNAi} (RRID:BDSC_34675), UAS-Atg18a^{RNAi} (RRID:BDSC_34714), UAS-Atg14^{RNAi} (RRID:BDSC_40858), UAS-Atg13^{RNAi} (RRID:BDSC_40861), UAS-Atg1^{RNAi} (RRID:BDSC_44034), UAS-Atg16$^{RNAi\#1}$ (RRID:BDSC_58244), UAS-Atg1 (OE-1) (RRID:BDSC_51654), UAS-Atg1B (OE-2) (RRID:BDSC_51655), UAS-ref(2)P^{RNAi} (RRID:BDSC_36111), UAS-RUBCN^{RNAi} (RRID:BDSC_43276), UAS-Atg16$^{RNAi\#2}$ (RRID:BDSC_34358), UAS-Pink1^{RNAi} (RRID:BDSC_38262), UAS-Park^{RNAi} (RRID:BDSC_37509), UAS-Drp1^{RNAi} (RRID:BDSC_51483), UAS-Marf^{RNAi} (RRID:BDSC_55189), UAS-Srl^{RNAi} (RRID:BDSC_33914), UAS-Su(z)12^{RNAi} (RRID:BDSC_33402), UAS-Kdm2^{RNAi} (RRID:BDSC_33699), UAS-trx^{RNAi} (RRID:BDSC_33703), UAS-Pc^{RNAi} (RRID:BDSC_33964), UAS-Gcn5^{RNAi} (RRID:BDSC_33981), UAS-rhi^{RNAi} (RRID:BDSC_34071), UAS-Utx^{RNAi} (RRID:BDSC_34076), UAS-HDAC1^{RNAi} (RRID:BDSC_34846), UAS-Set1^{RNAi} (RRID:BDSC_40931), UAS-mei-41^{RNAi} (RRID:BDSC_41934), UAS-Mt2^{RNAi} (RRID:BDSC_42906), UAS-gpp^{RNAi} (RRID:BDSC_42919), UAS-JIL-1^{RNAi} (RRID:BDSC_57293), UAS-mof^{RNAi} (RRID:BDSC_58281), UAS-Jarid2^{RNAi} (RRID:BDSC_40855), UAS-Jing^{RNAi} (RRID:BDSC_35750), UAS-E(z)^{RNAi} (RRID:BDSC_36068), UAS-Sce^{RNAi} (RRID:BDSC_67924), w^{1118}; UAS-GFP-E2F1$_{1\text{-}230}$ UAS-mRFP1-CycB$_{1\text{-}266}$ (RRID:BDSC_55110) and w^{1118};UAS-GFP-E2F1$_{1\text{-}230}$ UAS-mRFP-NLS-CycB$_{1\text{-}266}$ (RRID:BDSC_55111).

hiPSC culture conditions

The human induced pluripotent stem cells (hiPSC) line WTC-11 (Coriell Institute, GM25256), was derived in the Conklin laboratory (Kreitzer et al., 2013), the EGFP-TOM20 WTC hiPSC line was generated by the Allen Institute for Cell Science (Coriell Institute, AICS-0011) and expanded in the Ellison Stem Cell Core at the University of Washington (UW-AICS-0011).

Ionizing radiation treatment in *Drosophila* and hiPSCs:

A Cs-137 Mark I Irradiator was used to administer the proper irradiation dosage, according to instructed dosage chart. The remaining 1/3 of the females were not irradiated and were dissected within 1h of the others receiving irradiation treatment. After irradiation, the flies (with fresh, unirradiated males) were flipped onto a new vial of Standard Diet augmented with wet yeast at 25°C. ½ of the remaining females were dissected at 1-day post-insult (1 dpi). The remaining females were dissected at 2-days post-insult (2 dpi) (Figure 1A).

Drosophila and hiPSCs immunofluroscence analysis:

Germarium in the Drosophila ovary (Figure-1B) consist of Terminal filament (TF) cells (magenta, cuboidal) comprise the anterior tip of the ovary and connect to the Cap cells (CpCs) (magenta, planar);

Germline stem cells (GSCs) contain anterior spectrosomes (red, circular) which elongate (red, linear) during division; undifferentiated cystoblasts (CBs) contain spectrosomes (red, circular) or early fusomes (red, linear) but have no junctions to the CpCs; germ cell cysts (GCCs) are identified by presence of a branched fusome (red, branched); all GSCs and progeny contain nuclei (blue, circular); follicle stem cells (FSCs) (cerulean) can be identified by their distinct triangular morphology; mature GCCs are encapsulated by the prefollicle cells (PFCs) (lime green, planar) and proceed through oogenesis. Upon irradiation GSCs enter quiescence, shown by the absence of elongated spectrosomes and GSCs progenitor cells die. Later, GSCs exit quiescence and resume division, shown by the return of elongated spectrosomes and recovers the lost progenies.

Fly samples were dissected in cold PBS and fixed in 4% paraformaldehyde for 15 min at room temperature within 30 min of dissection. Samples were then rinsed in PBT (PBS containing 0.2% Triton X-100), blocked in PBTB (PBT containing 0.2% BSA, 5% normal goat serum) for at least one hour at room temperature. Samples were stored for up to 72 hours at 4℃ in PBTB. Samples were incubated with primary antibodies for 24 hours at 4℃. After PBT washes, fluorophore-conjugated secondary antibodies were utilized for 1.5–2 hours at room temperature in the dark. Samples were then washed with DAPI, diluted with PBT to 2 μg/ml, for 15 minutes to visualize nuclei, followed by two PBT washes. The samples were mounted in mounting medium (21 mL of Glycerol, 2.4ml of 10x PBS and 0.468g of N-Propyl Gallate) and analyzed on Leica SPE5 confocal and Leica SP8 confocal laser-scanning microscope. Primary and secondary antibodies are listed in the supplemental experimental procedures.

For hiPSCs, cells were washed with PBS (twice, 5 min each) and fixed in 4% paraformaldehyde in PBS for 15 min and blocked for 1 h in 3% BSA+0.1% Triton X-100. The cells were then incubated in primary antibody overnight at 4 °C, washed with PBS (thrice, 5 min each), incubated with the secondary antibody in 3% BSA+0.1% TritonX100 for 2 h at room temperature, washed in PBS (thrice,10 min each) and stained with 2 μg/ml DAPI diluted with 1X PBS for 15 min. Mounting media was composed of 21 ml of Glycerol, 2.4ml of 10X PBS and 0.468g of N-Propyl Gallate. Analysis was done on Leica SPE5 Confocal microscope using a ×63 objective and Leica Software. Antibodies used are listed in supplemental experimental procedures.

**DROSOPHILA ANTIBODIES**	**DILUTION**	**MANUFACTURER**
mouse anti-adducin	1:20	RRID:AB_528070
mouse anti-Lamin C	1:20	RRID:AB_528339
mouse anti-ATPsynβ	1:500	RRID:AB_301438
mouse anti-Fasciclin III	1:50	RRID:AB_528238
rat anti-cyclin E	1:200	A gift from Helena Richardson
rabbit anti-GFP antibody	1:500	RRID:AB_221569
mouse anti-mCherry antibody	1:500	RRID:AB_11133266
rabbit anti-mCherry antibody	1:250	RRID:AB_2650480
rat anti-Vasa antibody	1:20	RRID:AB_760351
rabbit anti-Dcp-1	1:100	RRID:AB_2721060
rabbit anti-DsRed	1:500	RRID:AB_10013483
chicken anti-GFP	1:2000	RRID:AB_300798
mouse anti-1B1	1:20	RRID:AB_528070
rabbit anti-H3K27me3	1:250	RRID:AB_ 2561020
anti-rabbit Alexa 488	1:250	RRID:AB_221544
anti-rabbit Alexa 568	1:250	RRID:AB_143157
anti-mouse Alexa 488	1:250	RRID:AB_2534069
anti-mouse Alexa 568	1:250	RRID:AB_2535773
anti-rat Alexa 568	1:250	RRID:AB_2534121
anti-chicken Alexa 488	1:250	RRID:AB_2762843
anti-mouse Alexa 647	1:250	RRID:AB_2535805
anti-rabbit Alexa 647	1:250	RRID:AB_2535812

hiPSC ANTIBODIES	DILUTION	MANUFACTURER
Anti-Oct-4	1:100	Santa Cruz
Anti-ATP synthase β	1:500	Abcam, Ab14730
Anti-cyclin E human	1:100	Santa Cruz, sc-481
Anti-TFE3	1:400	Sigma Prestige HPA023881
anti-HADHA	1:250	Abcam, ab54477
anti-Phospho-Histone 3 (Ser10)	1:250	Millipore Sigma, 06-570
GFP-Booster Alexa Fluor 488	1:300	ChromoTek, gb2AF488
Alexa Fluor 568 Phalloidin	1:300	Invitrogen, Cat: A12380
Alexa 488 or 568 or 647-conjugated secondary antibody	1:250	Molecular Probes

Categorization of reduced mitochondria

hiPSCs are considered containing reduced mitochondria when (1) few interconnected network of mitochondria tubules, but small independent mitochondria, is observed, (2) small mitochondria fragments scattered around the periphery of the nuclei, and (3) have significantly lower mitochondria: nuclei area compared to hiPSCs with undegraded mitochondria. hiPSCs are considered containing normal mitochondria when (1) extensive interconnected network of mitochondria tubules is observed, (2) network of mitochondria tubules form elongated polarized clusters extended from the nuclei, and (3) have significantly higher mitochondria: nuclei area compared to hiPSCs with the fragmented mitochondria.

GSCs are considered containing reduced mitochondria when (1) few interconnected network of mitochondria tubules, but small independent mitochondria, is observed, (2) small mitochondria fragments scattered around the periphery of the nuclei, and (3) have significantly lower mitochondria: nuclei area compared to GSCs with non- reduced mitochondria. GSCs are considered containing non-reduced mitochondria when (1) extensive interconnected network of mitochondria tubules is observed, (2) network of mitochondria tubules form elongated polarized clusters extended from the nuclei towards the anterior end of the germarium, and (3) have significantly higher mitochondria: nuclei area compared to GSCs with the fragmented mitochondria.

Mitochondria Area Analysis

Fixed number of confocal image slices showing only the mitochondria in the middle section of the cell were compressed using ImageJ Z-project feature. Area of interest (containing only the mitochondria of one cell) was selected using crop function. With an additional ImageJ plugin, Versatile Wand Tool, and using a fixed value for Value of Tolerance for all quantification, area of mitochondria was selected and measured.

H3K27me3 fluorescence intensity quantification

The mean pixel intensity of the H3K27me3 immunofluorescence staining was measured using ImageJ analysis software. Germline Stem Cells were identified by characteristic Adducin staining adjacent to cap cells in each germarium. Regions of Interest (ROI) were drawn around non-dividing GSC nuclei and mean pixel intensity in each ROI was measured inside each ROI. Mean pixel intensity was compared across control, 1 dpi and 2 dpi GSCs.

Image Deconvolution and 3D Imaging

Images taken from SPE5 confocal laser-scanning microscope were deconvoluted using Leica LIGHTNING software. 3D hiPSCs images were taken using GE DeltaVision OMX SR super-resolution microscope. Deconvoluted confocal images and OMX images were analyzed with Imaris (Bitplane)

program to construct respective 3D models in Maximum Intensity Projection (MIP) mode for *Drosophila* GSCs, and Normal Shading for hiPSCs.

Viral production

HEK 293FT cells were plated one day before transfection. On the day of transfection, the lentiCRISPRv2 plasmid containing the gRNA was combined with packaging vectors psPAX2 (a gift from Didier Trono, Addgene plasmid # 12260) and pMD2.G (a gift from Didier Trono Addgene plasmid # 12259) in the presence of 1 µg/µl of polyethylenimine (PEI) per 1 µg of DNA. Medium was changed 24 hours later, and the lentiviruses were harvested 48 and 72 h after transfection. Viral particles were concentrated using PEG-it (System Biosciences, Inc).

hiPSCs transduction and selection:

After hiPSCs were transduced with lentiCRISPR-v2/gRNA lentiviral particles, the media was changed the next day. 48h after infection, cells were selected with puromycin (1µg/ml) for two days and genomic DNA was extracted using DNAzol reagent (Invitrogen) and quantified using Nanodrop ND-1000. Genomic regions flanking the CRISPR target sites were PCR amplified with designed primers (Supplemental Table 3) using GoTaq DNA polymerase (Promega) and sent for Sanger sequencing to determine the insertion and deletion errors generated by CRISPR-Cas9 system in exon 2 PINK1 gene. The editing efficiency and KO score were determined using the Synthego ICE analysis tool. The mutant lines were further analyzed.

Western Blot Analysis

$5x10^4$ hiPSCs were plated on 35mm Matrigel coated plates and lysed in lysis buffer containing 20 mM Tris-HCl pH 7.5, 150 mM NaCl, 15% glycerol, 1% Triton x-100, 1 M ß-glycerolphosphate, 0.5 M NaF, 0.1 M sodium pyrophosphate, Sodium orthovanadate, PMSF, and 2% sodium dodecyl sulfate (SDS). Twenty-five units of Benzonase® Nuclease (EMD Chemicals, Gibbstown, NJ) was added to the lysis buffer right before use. The protein samples were combined with the 4× Laemmli sample buffer, heated (95°C, 5 min), run on SDS-PAGE (protean TGX pre-casted 4–20% gradient gel; Bio-rad) and transferred to nitrocellulose membrane (Bio-Rad) by semi-dry transfer (Bio-Rad). Membranes were blocked for 1 h with 5% milk or BSA and incubated in primary antibodies overnight at 4°C.The membranes were then incubated with secondary antibodies (1:10000, goat anti-rabbit or goat anti-mouse IgG HRP conjugate (Bio-Rad) for 1 hour and detected using the Immobilon-luminol reagent assay (EMP Millipore).

PRIMARY ANTIBODY	DILUTION	CATALOGUE NO.	MANUFACTURER
Beta-Actin	1:10000	4970	Cell Signalling
p-mTOR(Ser 2448)	1:1000	5536	Cell Signalling
mTOR	1:1000	2972	Cell SIgnalling
pS6	1:1000	2215	Cell Signalling
S6	1:1000	2117	Cell Signalling
Histone 4 (lys16) Acetylation (H4K16Ac)	1:1000	07-329	Millipore Sigma
PINK1 D8G3	1:1000	6946	Cell signalling

OCR Measurement Using Seahorse Cellular Flux Assay

The plates were pre-treated with 1:30 diluted Matrigel reduced growth factor (Corning). hiPSCs (WT and PINK1 KO) were seeded onto 96-well Seahorse plates at $3X10^4$ cells/well. Cells were cultured in DMEM with glucose overnight. Culture media were exchanged for base media (unbuffered DMEM

(Sigma D5030) supplemented with sodium pyruvate (Gibco, 1 mM) and with 25 mM glucose (for Mitostress assay) 1 hour prior to the assay. Substrates and selective inhibitors were injected during the measurements to achieve final concentrations of 4-(tri-fluoromethoxy) phenylhydrazone (FCCP, 1 μM), oligomycin (2.5 μM), antimycin (2.5 μM) and rotenone (2.5 μM). The OCR values were normalized to the number of cells present in each well, quantified by the hoechst staining (HO33342; Sigma-Aldrich) as measured using fluorescence at 355 nm excitation and 460 nm emission. Maximal OCR is defined as the change in OCR in response to FCCP compared to OCR after the addition of oligomycin.

Cell cycle Analysis via flow cytometry

hiPSCs, either WT, or PINK1 mutants, were seeded in 35 mm dishes at a density of $1.0x10^5$ cells. After 48 h of culture, cells were treated with 2 μM rapamycin or DMSO and 24 hours later, cells were harvested, and cell suspensions were pelleted and washed twice with PBS at 300 xg for 5 minutes. Cells were resuspended in 2% FBS in PBS and cells were fixed with 5 mL cold 70% ethanol for at least 24 hours (-20°C) and then centrifuged 10 minutes at 500 x g at 4°C. Cells were washed twice with 3 ml PBS at 400 xg for 5 minutes at 4°C and 500 ul of PI staining buffer was added (1X PBS, 50 μg/ml PI (Calbiochem), 2μg/ml Rnase A (Sigma) and 0.1% Igepal (Sigma)) for 3 hours at 4°C. Cell cycle analysis was performed using the FACS Canto II flow-cytometer (BD Biosciences). Data analysis was performed using the FlowJo software (Tree Star, Ashland, OR, USA).

Statistical analysis
All data are presented as the mean of $n \geq 3$ experiments with the standard error of the mean (SEM) indicated by error bars, unless otherwise indicated. Statistical significance was determined using chi-squared test (between timepoints) or Student's t-test (between conditions). Only p values of 0.05 or lower were considered statistically significant ($p > 0.05$ [ns, not significant], $p \leq 0.05$ [∗], $p \leq 0.01$ [∗∗], $p \leq 0.001$ [∗∗∗], $p \leq 0.0001$ [∗∗∗∗]). For analysis of quiescence, only fold changes of ≥1.6 were evaluated for significance. Data were compiled and analyzed with Excel for Mac (2020; Microsoft, Seattle, WA, USA).

SUPPLEMENTARY FIGURE LEGENDS:

SUPPLEMENTAL FIGURE 1: Role of mTORC1 in regulating insult-induced quiescence in female GSCs
(A) Representative confocal microscopy images of Rictor or Nrf2 RNAi KD from unirradiated, 1dpi, and 2dpi germaria stained with 1B1 (red, spectrosomes/fusomes), LamC (red, Cpc and TF) and DAPI (blue, nuclei). Dotted circle represents GSC (Scale bar 5μm). **(B)** Representative confocal microscopy images of control, Jarid2 and Pink1 RNAi KD from unirradiated, 1dpi, and 2dpi germaria stained with 1B1 (green, spectrosomes/fusomes), LamC (green, Cpc and TF), PH3 (red, proliferating cell nuclei) and DAPI (blue, nuclei). Dotted circle represents GSC (Scale bar 5μm). (**C-D**) Quantification of PH3 positive GSCs of control (**C**) Jarid2 RNAi KD (**D**) and Pink1 RNAi KD (**E**) from the images in S1**B**. (**F**) Quantification of GSCs with reduced mitochondria. Injury-induced mitochondrial fragmentation is observed in the G_0 stage of GSC cell cycle (n=203 for each).

SUPPLEMENTAL FIGURE 2: Autophagy-defective germline stem cells display impaired quiescence
(A) Schematic diagram of how UASp-EGFP-mCherry-Atg8a tandem fusion can be used to study autophagic flux. **(B)** Representative confocal microscropy images of nos>mCherry-Atg8a from unirradiated, 1 dpi, and 2 dpi germaria stained with mCherry (red, autophagosome/autolysosome), VASA (cyan) and DAPI (blue, nuclei). Dotted circle represents GSC (Scale bar 5 μm). **(C)** Bar graph depicting the proportion of punctae+ GSCs/total GSCs in nos>GFP-mCherry-Atg8a. **(D)** Immunofluorescence images of GSCs with respective core autophagy component RNAi KD. Stained with 1B1 (red, spectrosomes/fusomes), LamC (red, Cpc and TF) and DAPI (blue, nuclei).

Dotted circle represents GSC (Scale bar 5 μm). **(E)** Percentage of dividing GSC of control and Atg1 OE from unirradiated, 1 dpi, and 2 dpi germaria.

SUPPLEMENTAL FIGURE 3: Epigenetic proteins regulate GSC quiescence

(A-I) Representative confocal microscopy images of epigenetic genes RNAi KD from unirradiated, 1dpi, and 2dpi germaria stained with 1B1 (red, spectrosomes/fusomes), LamC (red, Cpc and TF) and DAPI (blue, nuclei). Dotted circle represents GSC (Scale bar 5μm). **(J)** A graph quantification of GSC division. Control GSCs divide at baseline level when unirradiated (28%), which sharply decreases at 1dpi (11%), and recovers to near baseline at 2dpi (23%). Epigenetic regulators, JIL1, Mof/KAT8, Mei-41/ ATR, Set1, Gpp/DOT1L are required for entry into quiescence. (n=3 for control. n=2 for JIL-1, mof, and mei-41/ATR). Following is the total number of GSCs quantified (n) for each condition in the order from unirradiated, 1dpi and to 2dpi time point: Control: 261, 119,90. JIL-1 RNAi: 135,128,103. Mof RNAi: 140,151,140. Mei-41 RNAi: 184,196,105. **(K)** Representative confocal microscopy images of GSCs with respective RNAi KD from unirradiated, 1dpi, and 2dpi germaria, which were used for fragmentation quantification on Fig. **5F**. stained with DAPI (blue, nuclei) and ATPsynβ (green, mitochondria) throughout the experimental timepoints (Scale bar 5μm). **(L-M)** Representative 3D reconstructed confocal microscopy images of (**L**) Tsc1-KD and (**M**) Atg3-KD GSC lines from unirradiated, 1dpi, and 2dpi time points stained with DAPI (blue, nuclei) and ATPsynβ (green, mitochondria). ('A' denotes anterior side, and 'P' denotes the posterior side of the GSC). Arrow points to the area of interest, where mitochondria are typically clustered (Scale bar 1μm). **(N)** Bar graph depicting the proportion of punctae+ GSCs/total GSCs in nos>-mCherry-Atg8a Pink1 RNAi and Jarid2 RNAi (left panel) and their respective representative confocal microscopy images (right panel). (Scale bar 5μm) (**O**) A graph quantification of the percentage of dividing GSCs of E(z) RNAi KD unirradiated, 1dpi, and 2dpi germaria. Following is the total number of GSCs quantified (n) for each condition in the order from unirradiated, 1dpi and to 2dpi time point: 165,27,38. **(P)** Representative confocal microscopy images of control GSCs from unirradiated, 1dpi, and 2dpi germaria stained with Dapi, cyclin E and ATPsynβ. **(Q)** Left panel: Top - GSC with reduced mitochondria; Bottom - GSC with non-reduced mitochondria. Right panel: Quantification of GSC reduced mitochondria area. (n=2 for both). Following is the the total number of GSCs quantified (n) for each condition. n= 51 for GSCs with reduced mitochondria, n=87 for GSCs without reduced mitochondria. **(R)** Summary model of Drosophila GSC showing an initial pules of FOXO activating Tsc1 which inhibits mTORC1, which in turn derepresses fission, mitophagy, and PRC1. Ultimately, mitochondrial fragmentation and degradation seem to constitute a "checkpoint" of sorts before entering quiescence. Then, in order to exit quiescence, H3K27me3 demethylase needs to reactivate specific genes, likely those involved in mitochondrial fusion and biogenesis.

SUPPLEMENTAL FIGURE 4: Mitochondrial degradation is required for iPSCs to enter quiescence

(A-C) Quantification of (**A**) mitochondrial reduction (**B**) and nuclear TFE3 in unirradiated and irradiated wildtype hiPSCs and **(C)** their respective representative confocal microscopy images. Scale bar- $50\mu m$. **(D)** WTCT cells treated with 2μM rapamycin for 3 hours, 7 hours or 24 hours, stained with ATP synβ (mitochondria, red), Cyclin E (green) and DAPI (blue) (Scale bar 5μm). **(E-F)** Western blot analysis for pulse chase experiment of wildtype hiPSC treated with either vehicle control (DMSO) or rapamycin (2 μM) for 7 hours **(E)** or 24 hours **(F)** followed by reversion (normal hiPSCs growth media) for another 96 hours. pmTOR, pS6 and H4K16Ac level is seen to be low by 7 or 24 hours post 2μM rapamycin treatment and their levels increase by 96 hours post reversion. **(G)** representative confocal images of hiPSCs treated with DMSO and Rapamycin(2μM) for 24 hours; stained for DAPI (blue) and TFE3 (red) (Scale bar 10 μm); a graph showing rapamycin treatment promotes significant nuclear localisation of TFE3 in hiPSCs. **(H)** Raw images of Figure 6C representative 3D-reconstructed OMX super resolution microscopy images of WTC cells treated with vehicle control (DMSO) or 2μM rapamycin for 24hrs. Stained with ATPsynβ (green), cyclin E (red) and DAPI (blue) and the white dotted line is used to quantify intensity profile in (D) (Scale bar 1 μm). **(I)** Representative high magnification 3D reconstructed OMX super resolution microscopy images of wildtype cells treated with DMSO vehicle or 2μM rapamycin for 24 hours, stained with ATPsynβ (mitochondria, green), Cyclin E (red) and DAPI (blue) (Scale bar 0.1μm). **(J)** Representative western blot analysis of Wildtype

treated with DMSO, 2μM rapamycin, EPZ-6438 and rapamycin+ EPZ-6438 for 24hrs and blotted with pmOTR and Beta-actin as a loading control. **(K)** Quantification of H3K27me3 intensity fold change in DMSO, 2μM rapamycin, EPZ-6438 and rapamycin + EPZ-6438 (images from Fig 6L were used for this quantification) for 24hrs, n=3. **(L)** Relative fluorescence of CycE and ATP synβ of Wildtype cells treated with vehicle (DMSO) or rapamycin for 7 hours (quantified from Fig. 6B). **(M)** Representative confocal microscopy images of CycE staining in wildtype hiPSC shows accumulation of CycE in DMSO, which is reduced upon rapamycin treatment and not rapamycin with MG132 (100nM, proteasomeme inhibitor). Scale bar =10μm.; a graph showing cyclin E intensity over DAPI area in DMSO, rapamycin and rapa with MG132 treated condition.Fig. A-F,H,L: Wildtype/control= WTC-Tom20. Fig. G: Wildtype= WTC**.**

Supplemental Figure 5: Mutant pool in PINK1 CRISPR/Cas9 knockout cells
(**A**) Pink1 structure with guide RNA location indicated and DNA sequencing chromatogram comparing wildtype (WT) PINK1 to a PINK1 knockout clone, showing the deletion in guanine, which creates an early stop codon, and another mutant PINK1 which shows a mixed pool of mutants and a loss of the wildtype sequence. **(B)** Western Blot showing lysates from Control wildtype WTC, PINK1 mutant WTC and PINK1 mutant WTC Tom-20, showing PINK1 protein knocked down in both mutant pools when compared to control. **(C)** Representative confocal microscopy images of wildtype and PINK1 knockout cells stained with DAPI and the pluripotent marker, Oct4. **(D-E)** Box plot data of 384-well qPCR array using wildtype and PINK1 knockout cells differentiated into all three lineages. Both wildtype (**D**) and PINK1 knockout cells (**E**) show their differentiation capacity into all three germ layers (endoderm, mesoderm and ectoderm). Fig. A-D: wildtype =WTC-Tom20.

SUPPLEMENTAL FIGURE 6: iPSCs require mitophagy to regulate Cyclin E
(**A-B, D, G-H**) Representative confocal microscopy images of control and/or Pink1-KD iPSCs, treated with either DMSO or 2μM rapamycin for 24 hours. (**A-B**) Deconvoluted images taken from SP8 confocal microscope. (**A**) Wildtype and Pink1-KD WTC cells stained with ATPsynβ (mitochondria, green), Cyclin E (red) and DAPI (blue) (Scale bar 10μm). (**B**) High magnification of Wildtype and Pink1-KD WTC cells stained with ATPsynβ (mitochondria, green), Cyclin E (red) and DAPI (blue) (Scale bar 5μm). (**C**) Quantification of cells without a dense cluster of mitochondria in wildtype vs Pink1-KD WTC treated with control DMSO or 2μM rapamycin for 24 hours, based on just the mitochondria (left panel) or mitochondria that co-localizes with cyclin E (right panel). Each group shows wildtype WTC increase in cells without dense cluster of mitochondria when treated with rapamycin vs DMSO, whereas Pink1-KD WTC cells maintains their dense cluster of mitochondria when treated with rapamycin. Black bar shows quantification for DMSO treated cells and grey bar for 2μM rapamycin treated cells. Data is from duplicate experiments. Following are the total number of cells quantified (n) for WT and Pink1-KO WTCT for DMSO and Rapamycin treated cells respectively: WT (without Cyclin E stain):1090,1022. WT (with cyclin E stain): 566, 533. Pink1-KO WTCT (without Cyclin E stain):725,754. WT (with cyclin E stain): 605,741. (**D**) Low magnification images (left panel) of control and Pink1-KD WTCT cells stained with ATPsynβ (mitochondria, green), Cyclin E (red) and DAPI (blue) (Scale bar 15μm). High magnification images (right manel) of wildtype WTCT or Pink1-KD mutant WTCT treated with either vehicle control (DMSO) or rapamycin (2μM) stained with DAPI (blue), CycE (red) and ATPsynβ (mitochondria, green) (Scale bar 5μm). (**E**) Representative 3D reconstructed OMX super resolution microscopy images of wildtype WTC or Pink1 mutant WTC stained with DAPI (blue), CycE (red), and ATPsynβ (mitochondria, green) (Scale bar 0.2μm). (**F**) Quantification of mitochondrial degradation in WTCT vs Pink1-KD mutant treated with either vehicle control (DMSO) or rapamycin (2μM), based on just mitochondria (left panel) or mitochondria that co-localizes with cyclin E (right panel), suggesting that Pink1 mutants can't degrade their mitochondria in response to rapamycin. Following are the total number of cells quantified (n) for WT and Pink1-KO WTCT for DMSO and Rapamycin treated cells respectively: WT (without Cyclin E stain):618,518. WT (with cyclin E stain): 512,414. Pink1-KO WTCT (without Cyclin E stain):616,701. WT (with cyclin E stain): 502,569. (**G-H**) Images of wildtype and Pink1 homozygous null mutant clone 1 and 2 treated with either vehicle control (DMSO) or rapamycin (2μM) stained with DAPI (blue), CycE (red) and ATPsynβ (mitochondria, green) (Scale bar 5μm). The image with merged color ATPsynβ and CycE is

used in the main figure **7C**. Grey dashes represents the boundary of the cell of interest. (**I**) Representative 3D reconstructed OMX super resolution microscopy images of Pink1 homozygous null mutant clone 2 stained with DAPI (blue), CycE (red), and ATPsynβ (mitochondria, green) (Scale bar 0.3µm). Fig. A-C,E: wildtype =WTC. Fig. C-D,F-G: wildtype= WTC-Tom20.

SUPPLEMENTAL FIGURE 7: iPSCs require mitophagy to regulate cell cycle halt.
(**A**) Wildtype and PINK1-KD WTC cells stained with PH3 (mitosis marker, red), and DAPI (blue) (Scale bar 15µm). (**B**) Quantification of PH3 positive cells in wildtype vs PINK1-KD WTC treated with DMSO control vs 2µM rapamycin for 24 hours. Wildtype WTC shows decrease in positive PH3 cells by half when treated with rapamycin compared to DMSO control, whereas PINK1-KD WTC shows slightly higher PH3 positive cells when treated with rapamycin compared to wildtype WTC cells. Data is from triplicate experiments. Following are the total number of cells quantified (n) for WT and PINK1-KO WTCT for DMSO and Rapamycin treated cells respectively: Control WTCT: 3994, 3849. PINK1-KO WTCT: 3957, 3981. (**C**) Representative confocal microscopy images of wildtype WTCT or PINK1-KD mutant WTCT treated with either vehicle control (DMSO) or rapamycin (2µM) stained with DAPI (blue), PH3 (proliferating cell nuclei, red) (Scale bar 15µm). (**D**) Quantification of PH3 incidence in wildtype WTCT vs PINK1-KD mutant treated with either vehicle control (DMSO) or rapamycin (2µM), suggesting that PINK1 mutants fail to halt cell cycle progression as efficiently as wildtype. Following are the total number of cells quantified (n) for WT and PINK1-KO WTCT for DMSO and Rapamycin treated cells respectively: Control WTCT:3439,2760. PINK1-KO WTCT: 3162,3184. (**E**) Schematic of wildtype hiPSCs with or without reduced mitochondria (left panel) and its quantification (right panel). (**F**) Representative confocal microscopy images of PINK1 homozygous null mutant clone 2 treated with either vehicle control (DMSO) or rapamycin (2µM) stained with DAPI (blue), PH3 (proliferating cell nuclei, red) (Scale bar 25µm). (**G**) Representative FACS cell cycle analysis traces from wildtype WTC or PINK1 mutant WTC treated with vehicle (DMSO) or rapamycin (2µM) for 24 hours. Fig. A-B,G: wildtype =WTC. Fig. C-E: wildtype= WTC-Tom20.

SUPPLEMENTAL TABLES AND LEGENDS

SUPPLEMENTAL TABLE 1: Excel file compiling all IR paradigm source data relevant to the experiments.
Data are organized into sheets that they represent in the main and supplemental figures, followed by genotype, time points and replicate number.

SUPPLEMENTAL TABLE 2: Guide RNAs for PINK1

Gene Name	Guide RNA	Exon	Forward Oligo	Reverse Oligo
PINK1	GCAAGCGTCTC GTGTCCAAC	2	CACC**GCAAGCGTCTC GTGTCCAAC**	AAAC**GTTGGACACGAGACGC TTGC**

SUPPLEMENTAL TABLE 3: Primers for PINK1 gene

Oligo Name	Primer Sequence (Forward)	Primer Sequence (Reverse)
PINK1	GGCTGAGCAGTAGAACCTGG	CAGGCACCTTTCCTGTGGAT

SUPPLEMENTAL TABLE 4: siRNA for Cyclin E

Cyclin E siRNA	Cyclin E siRNA Pool Sequence
siRNA # 1	GUACUGAGCUGGGCAAAUA
siRNA # 2	UGUCCUGGCUGAAUGUAUA
siRNA # 3	GGACAAUAAUGCAGUCUGU
siRNA # 4	GGAGGUGUGUGAAGUCUAU

Details of number of GSCs analysed in main figures(1-5):

FIGURE 1: (G) Following are the total number of GSCs quantified (n) for each condition in order of Unirradiated, 1dpi and 2dpi time point: Control: 271, 276, 187. Raptor RNAi: 135,66, 60. Rictor RNAi 342,281,220. Tsc1RNAi:120,95,67), Nprl3 RNAi: 156, 152, 128. Mitf RNAi: 353, 328, 265.

FIGURE 2: (D) . Following are the total number of GSCs quantified (n) for each condition in order of Unirradiated, 1dpi and 2dpi time point: Control: 215,202,228. Atg1 OE 270,119,156. Atg14 RNAi: 274,214,235. Atg18a RNAi: 302,169,197. Atg3 RNAi: 196,164,221. Atg12 RNAi: 363,216,302. Atg16: 505,253,281.

FIGURE 3: (D) . Following are the total number of GSCs quantified (n) for each condition in order of Unirradiated, 1dpi and 2dpi time point: Control: 265,297,225. Pink1 RNAi: 379,214,202. Park RNAi: 394,202,264. Drp1 RNAi:235,205,157. Marf/Mfn2 RNAi: 203,193,186. Srl/PGC1a RNAi: 246,203,152.

FIGURE 4: (B) Following is the total number of GSCs quantified (n) for each condition in the order from unirradiated, 1dpi and to 2dpi time point: Control: 264, 264,238. Pc RNAi: 304,192,197. Sce RNAi: 193,191,129. Jarid2 RNAi: 135,128,103. Trx RNAi: 291,275,221. Utx RNAi: 277,252,232

FIGURE 5: (F) Following are the total number of GSCs quantified (n) for each condition in the order from unirradiated, 1dpi and to 2dpi time point: Control: 63,59,40. Tsc1 RNAi: 63, 56, 50. Pink1 RNAi: 68,63,47. Sce RNAi: 76,69,48. Jarid2 RNAi: 70,100,106. Srl/PGC1a RNAi: 64,59,51. Utx RNAi: 67,64,52.
